# Seagrass sediment organic carbon burial rates are globally significant

**DOI:** 10.1038/s41467-026-79168-3

**Published:** 2026-11-04

**Authors:** Ariane Arias-Ortiz, Anna Lafratta, Phil Colarusso, James W. Fourqurean, Chuancheng Fu, Hilary Kennedy, Johannes R. Krause, Paul S. Lavery, Carmen Leiva-Dueñas, Nuria Marbà, Miguel A. Mateo, Inés Mazarrasa, Dimitris Poursanidis, Cristian Salinas, Oscar Serrano, Milica Stankovic, Nerea Piñeiro-Juncal, Axel Werner, Marlene Wesselmann, Carlos M. Duarte, Jordi Garcia-Orellana, Pere Masqué

**Affiliations:** 1 Departament de Física, Universitat Autonoma de Barcelona, Bellaterra, Barcelona, Spain; 2 School of Science & Centre for Marine Ecosystems Research, Edith Cowan University, Joondalup, WA Australia; 3 United States Environmental Protection Agency, Boston, Massachusetts, USA; 4 Institute of Environment, Florida International University, 11200 SW 8th St, Miami, FL USA; 5 Department of Biological Sciences, Florida International University, 11200 SW 8th St, Miami, FL USA; 6 Marine Science Program, Biological and Environmental Science and Engineering Division (BESE), King Abdullah University of Science and Technology (KAUST), Thuwal, Saudi Arabia; 7 School of Ocean Sciences, Bangor University, Menai Bridge, Wales UK; 8 Department of Ecoscience, Aarhus University, C.F. Møllers Allé, building 1131, Aarhus, Denmark; 9 Global Change Research Group, IMEDEA (CSIC-UIB) Institut Mediterrani d’Estudis Avançats, Miquel Marqués 21, Esporles, Spain; 10 Centre for Advanced Studies of Blanes, Spanish Council for Scientific Research, Blanes, Spain; 11 IHCantabria-Instituto de Hidráulica Ambiental de la Universidad de Cantabria, Santander, Cantabria Spain; 12 Foundation for Research and Technology—Hellas (FORTH), Institute of Applied and Computational Mathematics, N. Plastira 100, Vassilika Vouton, Heraklion, Greece; 13 Excellenece Center for Biodiversity of Peninsular Thailand, Faculty of Science, Prince of Songkla University, Hat Yai, Songkhla, Thailand; 14 Dugong and Seagrass Research Station, Faculty of Science,Prince of Songkla University, Hat Yai, Songkhla, Thailand; 15 Centro de Investigacions Mariñas, Departamento de Xeocencias Mariñas e Ordenación do Territorio, Universidad de Vigo, Vigo, Spain

**Keywords:** Carbon cycle, Climate-change mitigation, Marine biology

## Abstract

Crucial for coastal carbon sequestration, global seagrass organic carbon (OC) burial rates remain uncertain as early estimates were derived from mixed methodologies across geographically and taxonomically biased datasets. Here, we present a data-driven reassessment from a synthesis of 332 sediment cores spanning the last century. Results show that seagrasses bury OC at 25 ± 3 g OC m^−2^ yr^−1^ (geometric mean), with an area-weighted global average of 33 ± 9 g OC m^−2^ yr^−1^. Rates reflect a balance between sediment OC content and sediment accumulation, which share a negative exponential relationship driven by mineral dilution. Based on a revised mapped seagrass extent (247,700–366,400 km^2^), global burial scales to 6–15 Tg C yr^−1^. Although one-fourth of earlier benchmarks, seagrasses account for 3–4% to 3–6% of total oceanic OC burial despite covering <0.1% of the seafloor. Combined with mangroves and tidal marshes, vegetated coastal ecosystems contribute 8–13% of oceanic OC burial.

## Abstract (web summary)

Reassessing global seagrass carbon burial via 332 sediment cores reveals an area-weighted average of 33 ± 9 g OC m^-2^ yr^-1^ (6-15 Tg C yr^-1^ globally). Despite covering <0.1% of the seafloor, seagrasses drive 3-6% of total oceanic organic carbon burial.

## Introduction

The documented global extent of seagrass ecosystems is less than 0.1% of the area of the coastal ocean, yet they have been estimated to contribute 10–18% of its total organic carbon (OC) burial^1^. Earlier assessments, mainly indirect, estimated that seagrasses bury OC at an average rate of 138 ± 38 g OC m^−2^ yr^−1^ ^2^, comparable to other blue carbon ecosystems^3,4^ and roughly 30 times higher than rates in terrestrial forest soils^2^. Their global extent and their capacity to capture and bury OC over the long term have positioned seagrasses as a supportive element of nature-based climate solutions, with protection and large-scale restoration estimated to avoid up to ~ 1% of annual global greenhouse gas emissions^5^. This potential could be larger in countries that have extensive seagrass coverage and relatively low anthropogenic emissions^6^. Although interest in including seagrass management in Nationally Determined Contributions (NDCs) is growing, only 21 countries reference seagrasses^7^, and just a few (e.g., Australia^8^, Japan^9^) report them in their United Nations Framework Convention on Climate Change (UNFCCC) Greenhouse Gas inventory (GHGI), despite IPCC guidelines enabling such reporting^10^.

Climate change mitigation opportunities based on seagrass management require better understanding of their global distribution, loss and recovery trajectories^5,11^, and reference rates of OC burial in sediments^5,11^. Previous global estimates^2,10^ were limited by the scarcity of direct burial rate measurements and the use of heterogeneous methodological approaches that quantified different components of OC burial. The most commonly used global value of 138 ± 38 g OC m^−2^ yr^−1^^2^ was derived from a small number of direct radiocarbon-dated *Posidonia oceanica* mattes (*n* = 7)^1,12^ and a range of indirect approaches, including mass balance^12^, biomass accretion^13^, or sediment traps^14^ along with seagrass net community production (NCP)^15^ and burial of allochthonous OC^16^, integrating processes over timescales spanning days to millennia. To reduce methodological heterogeneity for national greenhouse gas reporting, the IPCC adopted a Tier 1 default value (43 g OC m^−2^ yr^−1^)^10^ based on six directly measured radiocarbon-based *P. oceanica* burial rates, excluding indirect or process-inferred estimates, thereby ensuring internal consistency but at the expense of strong taxonomic and regional bias.

The sparse abundance of direct estimates of OC burial rates in seagrass sediments contrasts with the greater number of estimates on sediment OC stock size available ( > 2700)^17^. Depth-integrated sedimentary OC stocks, paired with models or assumptions of OC lability, can inform about the amount of OC that could be remineralized and released to the atmosphere if seagrass meadows were degraded^18,19^. However, they do not capture the carbon burial efficiency of seagrass ecosystems, or their climate mitigation potential over the coming decades (50–100 years). The time required for seagrass sediments to accumulate a given OC stock can vary by more than an order of magnitude^20,21^, challenging the commonly held assumption that large OC stocks indicate high OC burial efficiency^22^. Indeed, criticism has arisen over the practice of using OC stocks to infer sequestration^23,24^, highlighting the need to consolidate direct estimates of OC burial rates in seagrass sediments.

Global assessments of OC burial rates for other coastal blue carbon ecosystems have been revisited and refined in recent years^3,4^. In tidal marsh and mangrove ecosystems, global OC burial rates have been reassessed through synthesis of studies that combined sediment OC content with sedimentation rates determined using radiometric dating, primarily ^210^Pb and ^137^Cs, and to a lesser extent, using marker and event horizons of known dates^4,25,26^. Beyond enabling the calculation of OC burial rates, these methods have allowed the identification of settings with no net sediment accumulation^27^, reinforcing the need to assess burial rates, rather than rely solely on stock data. Radiometric dating of seagrass sediments to derive OC burial rates has several challenges^28^. Primarily, sediment mixing, which is common in seagrass sediments, may overestimate rates or, in some cases, preclude their determination altogether. Secondly, the predominantly mineral and sandy nature of seagrass sediments^29^ can dilute radionuclide concentrations, limiting the depth and timescale over which reliable age models can be developed, potentially leading to a substantial bias in published OC burial rates toward muddier depositional environments.

The rise of blue carbon strategies has greatly stimulated research into OC burial by seagrasses, resulting in numerous site-specific studies across species and geographic regions. These studies have generally reported burial rates 3–7 times lower than previously estimated globally^30^. However, a comprehensive and updated synthesis of OC burial in seagrass ecosystems is still lacking, and available estimates remain scattered throughout the literature. Given the importance of seagrass meadows to the oceanic carbon budget^1^, we synthesized OC burial rates in seagrass sediments over a century timescale to provide a harmonized global estimate and assess global patterns. Our analysis integrates downcore sediment profiles of OC content, their δ^13^C signatures, and ^210^Pb specific activities along with ancillary variables and relevant metadata, resulting in a comprehensive, open-source dataset^31^. These data were integrated with global classification schemes such as seagrass bioregions^32^ and nearshore coastal geomorphological typologies^33^, alongside seagrass genus, to understand its variability. Consolidating knowledge on seagrass OC burial rates contributes to refining the global ocean carbon budget, validates estimates of seagrass OC fluxes, and supports the revision of the global default (Tier 1) value^10^ for use in national GHGI reported to the UNFCCC.

## Results and discussion

The dataset comprised 332 sediment profiles from seagrass ecosystems across a latitudinal range of 64°N to 35°S, including vegetated (89%) and unvegetated (11%) sediments. Unvegetated cores represented adjacent non-vegetated areas generally paired with nearby seagrass cores, separated by distances ranging from 6 m to 3 km. Overall, a total of 220 records from our primary dataset reported quantifiable OC burial rates (208 from vegetated and 12 from unvegetated sediments). In the remaining 112 cores, rates could not be resolved equally due to intense sediment mixing extending well below the surface and the absence of net sediment accumulation. The latter was inferred from the absence of excess ^210^Pb, the unsupported fraction of ^210^Pb derived from atmospheric fallout, which accumulates on the seafloor over time and serves as a tracer for recent sediment deposition^34^.

**Fig. 1:**
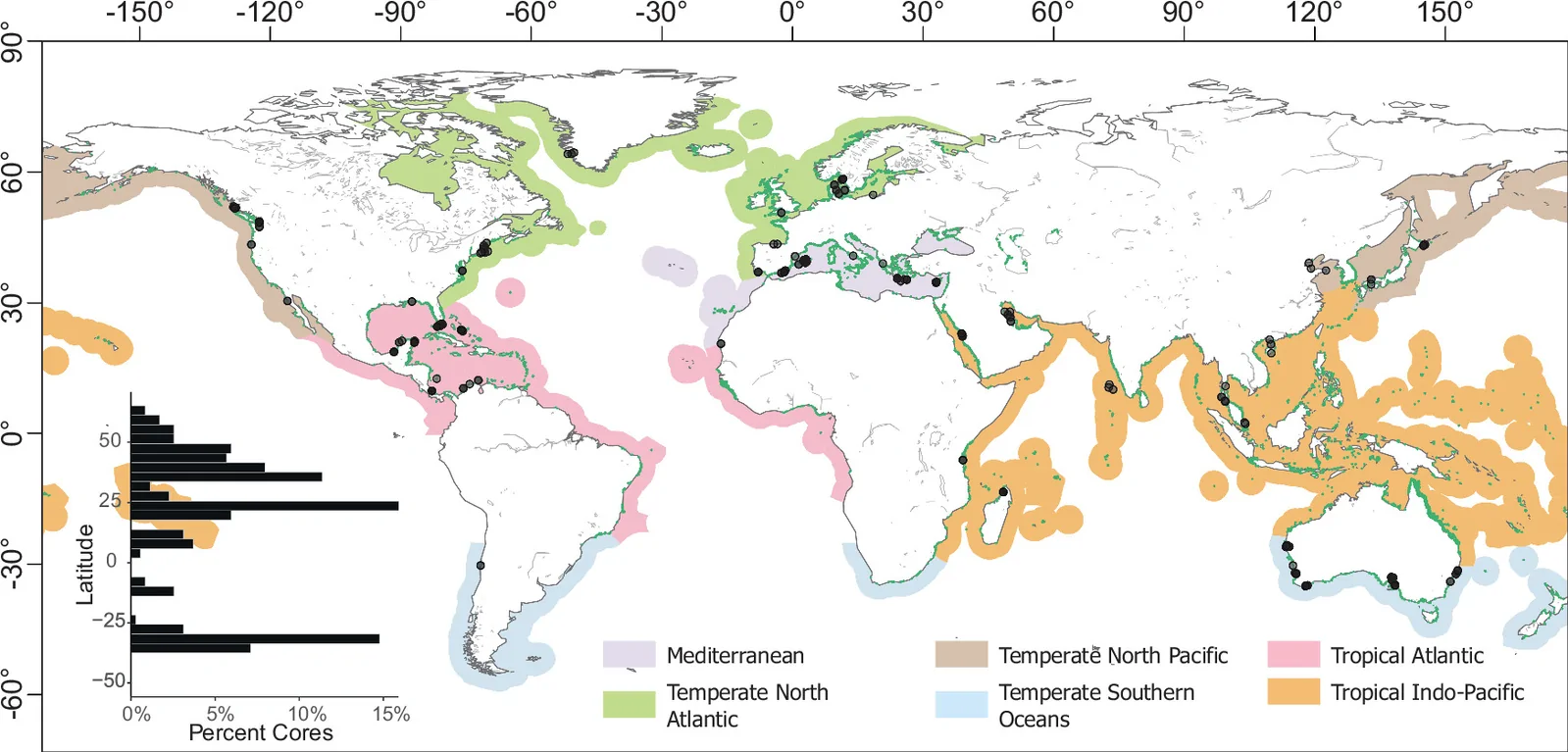
Location of sites included in the synthesis superimposed on global seagrass bioregions. Black dots represent sediment cores, with point opacity reflecting data density (overlapping or proximate core locations). Dark green coastline indicates the global seagrass distribution^79^. The inset bar graph shows the percentage of cores by 5° latitudinal bands.

Consistent with reported OC stock assessments^17,18,35^, data were geographically concentrated in Australia, North America, and the Mediterranean Sea, while data were sparse for South America, Africa, South and Southeast Asia, despite the extensive seagrass distribution in the latter. By bioregion, 16% of the estimates originated from the Mediterranean, while 20 and 26% came from the Tropical Atlantic and Southern Oceans (primarily from the tropical Western Atlantic, and Australia, respectively), with the remaining regions contributing 12-13% each (Fig. 1).

### Organic carbon burial rates in seagrass sediments

**Fig. 2:**
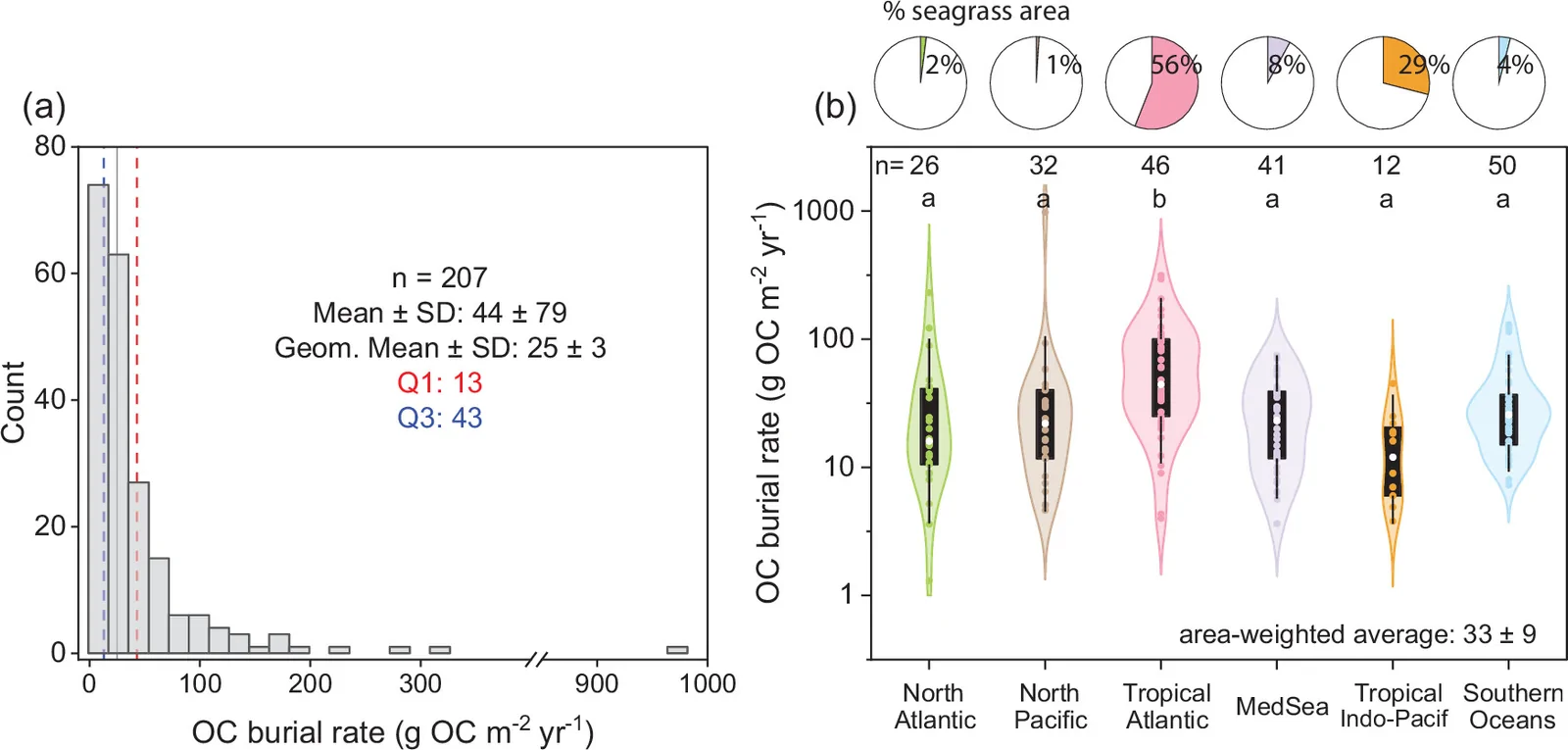
Organic carbon (OC) burial rates in seagrass sediments with net positive accumulation. **a** Binned frequency of all OC burial rates in vegetated sediments. **b** OC burial rates by bioregion. Q1 and Q3 in panel (**a**) represent the first and third quartiles, indicating burial rates for the bottom 25% and 75% of the dataset, respectively. Pie charts in panel (**b**) show the percentage contribution of each bioregion to the total seagrass area (Supplementary Data 1). The area-weighted average was calculated using mapped seagrass distribution as weights, applied to regional geometric means (Supplementary Table S2). In panel (b), different lower-case letters represent significant differences between bioregions as a result of a Kruskal–Wallis Dunn’s multiple comparison test with *p* < 0.05 adjusted using the Bonferroni procedure (*p *< α/2). Numbers above letters denote the number of cores per bioregion.

Estimates of OC burial rates within depositional vegetated sediments spanned three orders of magnitude, ranging from 1.3 ± 0.5 g OC m^−2^ yr^−1^ (Ameralik, Greenland^36^) to 976 ± 132 g OC m^−2^ yr^−1^ (Swan Lagoon, Shandong, China^20^), the latter being a high-end outlier in the global distribution (Fig. 2a). Omitting rates from sediments exhibiting intense downcore mixing, which are subject to greater uncertainty, did not reduce the range substantially, which still spanned two orders of magnitude (3.6–314 g OC m^−2^ yr^−1^; Supplementary Table S1). Regional and local ranges were similarly pronounced within bioregions, exhibiting over two orders of magnitude variability within the North Atlantic and North Pacific, and an order of magnitude in the Mediterranean, the Tropical-Indo Pacific, Tropical Atlantic, and the Australian Southern Ocean (Fig. 2b and Supplementary Table S2).

**Fig. 3:**
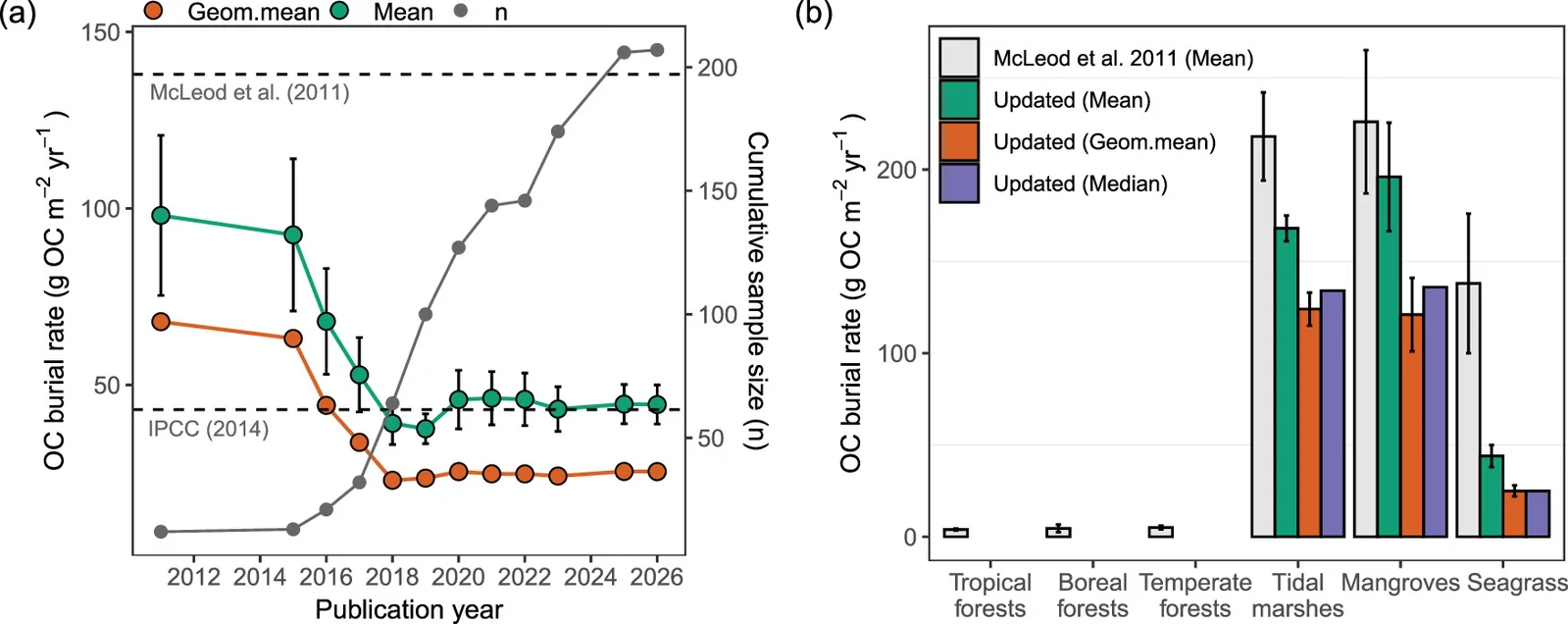
Summary statistics of contemporary organic carbon (OC) burial rates in seagrass sediments. **a** Cumulative summary statistics of OC burial rates by publication year, calculated from 2010 onward, for seagrass cores exhibiting net positive accumulation (**b**) comparison with OC burial rates in soils of terrestrial forests, sediments of other coastal vegetated ecosystems and previously compiled estimates. In panel (**a**), median estimates are not shown because they overlap with geometric mean values. Equivalent statistics for both depositional and non-depositional seagrass sediments are presented in Supplementary Fig. S3. Horizontal dashed lines indicate the IPCC Tier 1 estimate and the mean OC burial rate reported by McLeod et al.^2^. In panel (**b**), results from previous and updated assessments are shown, with revised estimates from Breithaupt and Steinmuller^4^ for mangroves, Wang et al.^3^ for tidal marshes, and this study for seagrasses. Error bars represent the standard error of the mean and the distance from the central tendency to the bounds of the 95% confidence interval for the updated geometric mean estimates.

Organic carbon burial rates in depositional seagrass sediments followed a log-normal distribution, strongly skewed toward low values (Fig. 2a). Under these conditions, the geometric mean was used to best reflect the central tendency. Considering all depositional seagrass sediments, the global geometric mean OC burial rate was 25 ± 3 g OC m^−2^ yr^−1^ (95% CI: 22–29; *n* = 207), a value that remained similar even when excluding records affected by intense sediment mixing (27 ± 2 g OC m^−2^ yr^−1^; 95% CI: 23–31; *n *= 127). Remarkably, this pooled, global estimate has stabilized with the rapid addition of new data since 2018, resulting in low sensitivity to additional measurements and reduced standard errors (Fig. 3a and Supplementary Fig. S1). However, it remains influenced by the uneven geographical distribution of observations, with OC burial rates from Australia, the USA, and Spain disproportionally contributing to the dataset (Supplementary Fig. S2). Furthermore, 16% of the studied vegetated sediments had undetectable excess ^210^Pb, suggesting negligible net OC burial at decadal-centennial time scales. Including these non-depositional sites yielded a lower global estimate of 20 g OC m^−2^ yr^−1^ (95% CI: 15–23; *n* = 255).

Organic carbon burial rates in seagrass sediments from the Tropical Atlantic were the highest (Kruskal–Wallis test, H = 28.5, df = 5, *p* < *0.05*), whereas no significant differences were observed among the other five bioregions (*p* > *0.05*) (Fig. 2b). Considering the different seagrass extent across bioregions, we calculated an area-weighted average OC burial rate of 33 ± 9 g OC m^−2^ yr^−1^ (95% CI: 25–41), which was comparable to the geometric mean estimate (Fig. 2a). While this area-weighted estimate is less influenced by sampling density, it is driven by bioregions with the largest seagrass coverage. Consequently, it remains sensitive to uncertainties in mapped seagrass area and to the limited data available from the Tropical Indo-Pacific, where regional means are still dependent on sample size (Supplementary Fig. S1). Nevertheless, the two values are statistically similar, despite the different sources of uncertainty, and provide robust and complementary estimates of global OC burial rates in seagrass sediments.

Using the same metrics, our revised global estimates of OC burial rates were consistently lower than those reported in earlier syntheses. The arithmetic mean for vegetated sites with net positive accumulation (44 ± 79 g OC m^−2^ yr^−1^) was 68% lower than the widely cited value report by McLeod et al.^2^ (138 ± 38 g OC m^−2^ yr^−1^). When compared to the IPCC Tier 1 global default value (43 g OC m^−2^ yr^−1^, 95% CI: 20–70)^10^, which is based on a geometric mean, the geometric mean of our dataset (25 ± 3 g OC m^−2^ yr^−1^) was ~40% lower, while still falling within the IPCC confidence interval. A similar pattern was observed for the area-weighted average, which also fell below the IPCC central estimate but within its 95% confidence interval (Fig. 3). Including cores with negligible accumulation would have yielded a global average OC burial rate ~ 75% lower than the global arithmetic mean reported by McLeod et al.^2^, while the global median would have been ~ 50% lower than the IPCC geometric mean.

The differences in sediment OC burial rates relative to previous syntheses stem from the inclusion of ~ 50 times more direct estimates than previous global assessments, a broader geographic coverage, and the systematic use of ^210^Pb geochronology, which integrates OC burial over a consistent decadal-centennial timescale. The largest differences were observed relative to McLeod et al.^2^, which were based on limited direct measurements and relied on indirect approaches representing short-term sequestration processes. Such methods can often result in higher values due to their focus on rapidly cycling carbon pools with a greater contribution of labile organic matter. In contrast, our analysis captures the mean in situ accumulation of OC in seagrass sediments over the last century, excluding export and burial beyond the meadows, sequestration in biomass, and short-term net ecosystem uptake. While our estimates include surface sediment OC that may not have undergone complete decomposition, this contribution was small, although variable. Over a centennial timescale, 51% of vegetated sediment profiles had stable OC concentrations with depth, 39% decreased, and 10% increased, yielding a median bottom-to-surface OC concentration ratio of 0.70.

Global OC burial rates for mangroves and tidal marshes have been reassessed based on estimates primarily derived from ^210^Pb-based approaches, allowing cross-ecosystem comparison. On a per-area basis, the geometric mean and median OC burial in seagrass sediments were about one-fifth those of tidal marshes (geometric mean: 124 ± 9 g OC m^−2^ yr^−1^; median: 133 g OC m^−2^ yr^−1^)^3^ and mangroves (geometric mean: 121 ± 19 g OC m^−2^ yr^−1^; median: 136 g OC m^−2^ yr^−1^)^4^. While this challenges previous assertions that seagrasses bury OC at rates comparable to mangroves and tidal marshes, it confirms that seagrass sediment OC burial per unit area is still approximately six to eight times higher than that of terrestrial forest soils (Fig. 3b).

### Interplay between organic carbon content and sedimentation rates

The nearly three orders of magnitude range in OC burial rates across seagrass sediments arises from the combined variability of its two primary components, OC content and sediment mass accumulation rates (MAR), each spanning roughly two orders of magnitude (Table 1). In principle, this could produce up to a four order of magnitude range in burial rates, but such extremes were not observed because high MAR and high OC content did not co-occur in the same sediments (Table 1)

**Table 1:** Summary of organic carbon (OC) burial rates, sediment mass accumulation rates (MAR), and OC content in seagrass sediments dated to the last century

Parameter		*n*	Range	Mean	SD	Median	Geom. Mean	Use Value
OC Burial rate (g C m^-2^ yr^-1^)	Global	207	1.3–976	44	79	25	25	Geom. Mean: 25 95% C.I.: 22–29
	w/ negligible accumulation	255	0–976	35	73	20	-	Median: 20 95% C.I.: 15–23
MAR (g cm^−2^ yr^−1^)	Global	207	0.02–2.8	0.32	0.36	0.21	0.21	Geom. Mean: 0.21 95% C.I.: 0.180.23
	w/ negligible accumulation	255	0–2.8	0.26	0.35	0.16	-	Median: 0.16 95% C.I.: 0.14–0.19
OC (%)	Global	207	0.09−11	1.9	1.8	1.4	1.3	Geom. Mean: 1.3 95% C.I.: 1.1–1.4
	w/ negligible accumulation	255	0.05–11	1.7	1.7	1.05	1.04	Geom. Mean: 1.04 95% C.I.: 0.9–1.2

The ‘use value’ represents measures of central tendency with 95% confidence intervals. The geometric mean was used for variables with log-normal distributions, while the median was used for variables that did not follow a normal distribution, even after log transformation. SD stands for standard deviation. The category w/ negligible accumulation indicates summary statistics that include cores exhibiting negligible sediment accumulation over the last century. Summary statistics by bioregion can be found in Supplementary Table S2.

Mean OC content in seagrass sediments accumulated over the past century ranged from 0.09% to over 10%, with a geometric mean of 1.3% of dry weight and a global mean of 1.9% (Table 1), roughly 1.5 times higher than the median (0.7%) and mean (1.3%) values reported by Krause et al.^17^ for seagrass sediments globally. The difference between our OC content estimates and those reported globally may be partially explained by the inclusion in earlier studies of downcore values without consistent depth or age standardization, whereas our estimates were standardized to a common timescale. Sedimentary OC exhibited δ¹³C values ranging from − 10.5 to − 26.9‰, which, relative to seagrass isotopic signatures (− 5.2 to − 15.3‰), resulted in seagrass-sediment isotopic offsets ranging from 0.02 to 23.0‰ (median: + 6.1 ± 1.0‰). The median isotopic offset was similar to the global offset of + 6.4 ± 3.4‰ by Kennedy et al.^16^, who inferred that seagrass-derived organic matter contributed a median of 51% to sediment OC, with remaining contributions from phytoplankton and terrestrial organic matter.

Sediment MAR in vegetated records ranged from 0.02 to 2.8 g cm^−2^ yr^−1^, with a geometric mean of 0.21 g cm^−2^ yr^−1^ (95% C.I.: 0.18−0.23, *n* = 207) (Table 1). This corresponded to a vertical accretion rate of 2.3 mm yr^−1^ (2.0–2.6, 95% C.I.), calculated using a median dry bulk density of 0.90 g cm^−3^ estimated from seagrass sediments accumulated over the last century. However, if records showing no net sediment accumulation were included, the median estimate decreased to 0.16 g cm^−2^ yr^−1^ (95% CI: 0.14–0.19), corresponding to sediment accretion rates of 1.5–2.1 mm yr⁻¹.

**Fig. 4:**
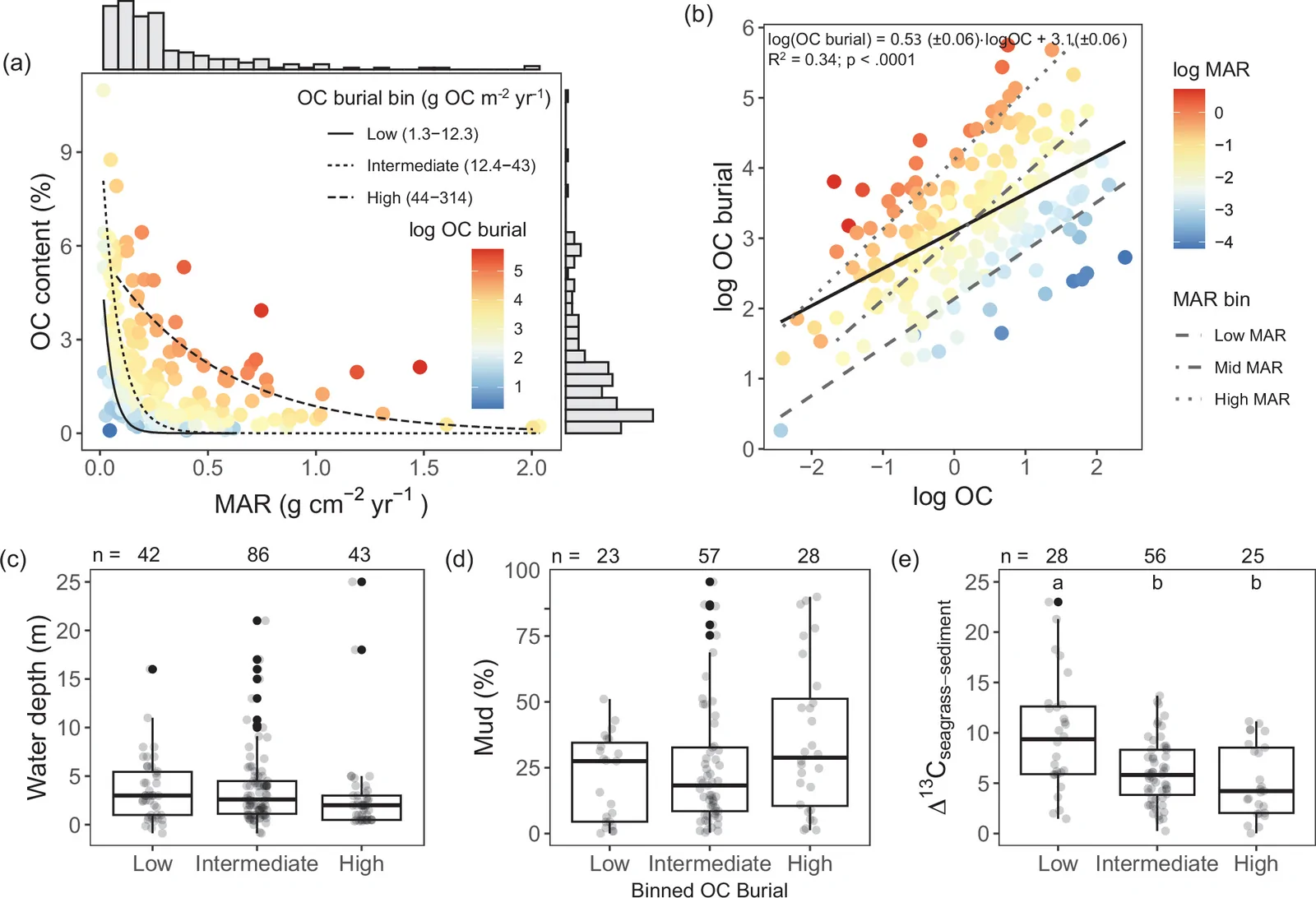
Environmental and geochemical factors associated with organic carbon (OC) burial rates in seagrass sediments. **a** Relationship between sediment OC content and mean mass accumulation rate (MAR) at vegetated seagrass cores. The color gradient represents OC burial rates on a log scale, with exponential fits showing varying slopes of the relationship for binned burial rates, grouped by percentiles: low (0–25%), intermediate (25–75%), and high (75–100%), with corresponding rate ranges shown in parentheses and in Supplementary Table S3. Regression statistics are summarized in Supplementary Table S4. Histograms on the top and right axes show marginal distributions of MAR and OC, respectively. **b** Log-log relationship of OC burial and OC content in vegetated sediments. The color gradient represents MAR on a log scale, with dashed lines showing linear fits for each MAR percentile: low (0–25%), intermediate (25–75%), high (75–100%) and a solid line representing the overall mean linear fit across all points (equation shown; regression stats for each percentile in Supplementary Table S4). **c**–**e** Boxplots showing environmental and geochemical characteristics across OC burial bins (Low, Intermediate, High): (**c**) water depth, (**d**) mud content, and (**e**) isotopic difference between seagrass and sedimentary OC. Boxes show the interquartile range, horizontal lines indicate the median, and whiskers extend to 1.5 times the IQR, individual data points are overlaid and solid dark data points represent outliers. Mass accumulation rates larger than 3 g cm^−2^ yr^−1^ and OC burial larger than 500 g C m^−2^ yr^−1^ (*n* = 1) have been excluded as outliers. Significant differences in environmental or geochemical characteristics between OC burial bins in the boxplots are indicated with letters. Numbers above letters denote the number of cores per OC burial bin.

Sediment OC content and MAR followed a negative exponential relationship, with higher sedimentation rates diluting OC concentrations due to greater mineral input (Fig. 4). Rather than discrete thresholds, ranges of OC and MAR that produced low, intermediate, and high OC burial rates had substantial overlap (Supplementary Table S3), with OC content defining the potential for OC burial, and MAR determining the extent to which this potential is realized. High sediment OC burial rates ( > 43 g OC m^−2^ yr^−1^) generally occurred when OC content was above median values, and MAR was moderate ( > 0.18 g cm^−2^ yr^−1^), though peak rates could also occur at very low MAR ( ~ 0.08 g cm^−2^ yr^−1^) if OC content was near-maximum (8%). In contrast, low OC burial rates ( < 13 g OC m^−2^ yr^−1^) were observed at sites where either OC content and MAR were below median values, or where MAR was relatively high ( ~ 0.6 g cm^−2^ yr^−1^) but OC content was extremely low ( ~ 0.09%) (Supplementary Table S3). Notably, at MAR below 0.1 g cm^−2^ yr^−1^, OC content spanned the full range observed across the dataset (0.09–10%), highlighting the potential for slow-accumulating environments to exhibit variable OC content and stocks. (Fig. 4a).

Differences in the rate at which OC content declines with increasing MAR represent a continuum of seagrass depositional environments, each with its own balance of sedimentation and OC dynamics promoting OC burial (slopes in Fig. 4a). Meadows with high to intermediate burial rates typically occurred in settings characterized by high seagrass organic matter contribution to sediment OC at shallow depths (Fig. 4c–e), where moderately elevated MAR could promote rapid burial, limiting OC exposure to oxidation and enhancing long-term preservation^37^. Log–log regressions of OC burial against sediment OC content revealed steeper slopes at sites with high MAR (Fig. 4b and Supplementary Table S4), suggesting that OC may be more efficiently preserved in rapidly accumulating sediments. In contrast, low burial rates were associated with environments characterized by low sedimentation and a potentially low contribution of seagrass organic matter to sediment OC, reflected in a larger offset between seagrass isotopic signals and that of sediment OC compared to high-burial settings (Fig. 4c–e).

Sediments colonized by persistent seagrass species of the genera *Posidonia* and *Thalassia*, or by mixed meadows of persistent and opportunistic taxa (e.g., *Posidonia* with *Amphibolis*, or *Thalassia* with *Syringodium*), exhibited the highest sediment OC content (medians between 2.2%, 1.4% and 1.9%, respectively; *p* < 0.05; Supplementary Fig. S4a), consistent with previous findings that persistent species support the largest sediment OC stocks globally^17^. Mechanistically, persistent genera feature large belowground biomass investments and highly recalcitrant structural tissues that resist microbial mineralization^35,38^. However, while these genera exhibited high-to-intermediate OC burial rates (Supplementary Fig. S4c), burial was not strictly proportional to sedimentary OC content. Deviations from the mean OC burial-OC content relationship were structured by MAR (Fig. 4b). At sites with low MAR, OC burial was lower than expected based on OC content alone, whereas at sites with high MAR, burial exceeded values predicted by OC content.

This pattern was particularly evident in *Posidonia* colonized sediments, which, despite having the highest OC content, only achieved intermediate OC burial rates due to characteristically low sedimentation rates (Supplementary Figs. S4b, c and S5), reflecting their adaptation to thrive in environments with low sediment inputs and consequently, low turbidity and high light availability^39^. In contrast, *Thalassia*-colonized sediments combined moderate to high OC content with elevated MAR, resulting in the highest OC burial efficiency (Supplementary Fig. S4c, S5). These observations were dominated by *Thalassia* beds in the tropical W. Atlantic, particularly in the lime-mud deposits of Florida Bay banks^40^ and the Bahamas^41^.

Sediments colonized by *Halophila* spp. in China^20^, Australia^42^, and the eastern Medtirerranean^43^, showed some of the highest MAR, yet yielded comparatively low OC burial rates due to low sediment OC content. This might be related with the low belowground biomass and rapid tissue turnover characteristic of colonizing taxa^35^. *Zostera* meadows displayed the widest range of both MAR and OC content among all genera (Supplementary Fig. S4), which may reflect the broad habitat envelope occupied by this genus relative to other seagrasses^44^ and the possibility that *Zostera* and associated burial rates might not be well described using a single genus^45^.

Coastal geomorphology is an important factor shaping global patterns of blue carbon stocks, particularly in mangroves^46^, and new evidence suggests similar trends in seagrasses^17,35^. However, when assessing OC burial rates, global coastal geomorphology did not explain relevant differences in OC burial. Seagrass meadows in karstic systems exhibited the highest sediment OC burial rates, with these environments also showing the highest MARs (Supplementary Fig. S6). This pattern could be attributed to the limited representation of karst systems in other regions, as cores in this category were predominantly associated with karstic settings in the Tropical W. Atlantic. Indeed, when the karst geomorphic category was excluded, or similarly, when cores from Florida Bay and the Bahamas were removed, coastal geomorphology had no significant effect on global OC burial rates.

### Organic carbon burial in vegetated and adjacent unvegetated sediments

About 18% of the cores in this study, including both vegetated and bare sediments, were collected in non-depositional environments, as indicated by negligible excess ^210^Pb specific activities. Notably, the proportion of non-depositional records was twice as high in unvegetated sediments (30%) compared to vegetated ones (16%), highlighting the role of seagrass cover in stabilizing sediments and OC stocks beneath their meadows^47^. However, whether depositional environments have a greater influence on OC burial than seagrass traits or the reverse depends on the geomorphic and temporal context.

Seagrasses contribute to sediment stabilization by influencing local hydrodynamics, reducing flow speed, and ameliorating turbulence^48^, facilitating the deposition of fine particles, including both seagrass and non-seagrass derived OC^14,16,49^. Their network of roots and rhizomes further anchors sediments, mitigating resuspension and erosion^50^ that could otherwise enhance OC remineralization. This stabilizing effect is likely most significant in settings where seagrass can reduce bottom shear stress below the critical threshold for sediment resuspension^51^. In higher-energy environments, or those already depositional, hydrodynamic conditions may independently govern sediment and OC burial regardless of vegetation cover^52^.

**Fig. 5:**
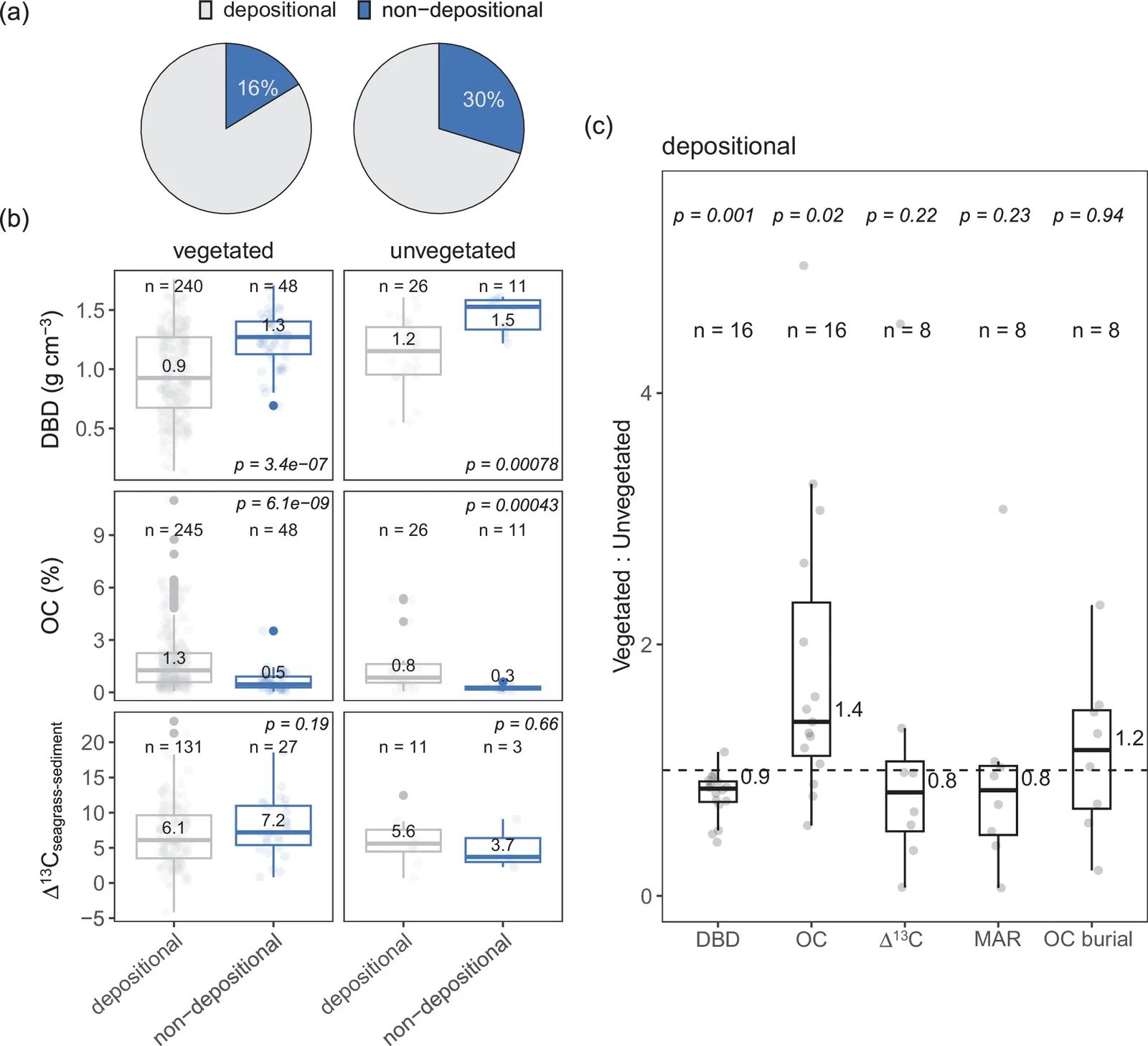
Comparison of depositional and non-depositional sites across seagrass-vegetated and adjacent bare sediments. **a** Proportion of depositional and non-depositional records within vegetated and unvegetated sediments, where gray represents depositional and blue non-depositional records. **b** Comparison of sediment properties and contributions of seagrass-derived organic carbon (OC) between depositional and non-depositional sediments in vegetated and unvegetated habitats. **c** Boxplots of vegetated-to-unvegetated sediment ratios of dry bulk density (DBD), OC content, Δ^13^C_seagrass-sediment_, mass accumulation rate (MAR), and OC burial rates across paired sampling sites. The horizontal dashed line represents a ratio of 1. Δ^13^C_seagrass-sediment_ reflects the difference between the δ^13^C isotopic signal of seagrass plant material and that of the sediment. In panel (**c**), significant differences between paired samples were analyzed using a paired t-test (DBD and Δ^13^C_seagrass-sediment_) and a Wilcoxon signed-rank test (OC content, MAR, OC burial) (*p *< 0.05). Paired comparisons in panel (**c**) included 16 sites for DBD and OC content and 8 observations for Δ^13^C_seagrass-sediment_, MAR and OC burial.

Across the dataset, sediments in non-depositional environments had significantly higher sediment dry bulk densities (DBD: 1.3–1.5 g cm^−3^) and lower OC content (0.30–0.50%), even in areas colonized by seagrasses. In contrast, depositional environments, both vegetated and unvegetated, exhibited lower DBD and higher OC content (DBD: ~ 0.9–1.2 g cm^−3^; OC: 0.8-1.3%) (Fig. 5b). Despite differences in sediment characteristics between depositional and non-depositional habitats, the isotopic offset between seagrass and sediment OC (Δ^13^C_seagrass-sediment_) was similar across both habitats (Fig. 5b), pointing to a shared influence of seagrass-derived material on sedimentary OC, even if their capacity to accumulate and retain OC differs.

At the local scale where seagrass meadows and adjacent bare sediments were sampled within the same sites (separated by distances ranging from 6 m to 3 km), OC content and dry bulk density differed between habitats, whereas isotopic similarity to seagrass-derived organic matter, sediment accumulation and OC burial rates did not (Fig. 5c). Although limited in scope, these findings highlight the spillover effect of seagrass meadows, suggesting that seagrasses influence burial at the site level by contributing organic matter to nearby unvegetated sediments, as evidenced by their similar isotopic signatures of seagrass-derived OC^16^ (Fig. 5c). This is consistent with findings from the Bahamas^41^ or Bermuda^53^, where seagrass-derived OC is redistributed across the depositional seascape regardless of seagrass abundance at a given location. In Bermuda, seagrass loss has been shown to reduce the regional pool of seagrass-derived OC across both vegetated and unvegetated areas^53^. (Fig. 5a)

Local sedimentation dynamics, together with seagrass presence and lateral redistribution of seagrass OC across vegetated–unvegetated boundaries^16,54^, can therefore result in similar OC burial rates between seagrass and adjacent bare sediments^40^. Together, these findings support a proposed hierarchy of controls on OC burial, with proximity to seagrass-OC sources as primary, hydrodynamic exposure as secondary, and vegetation traits as tertiary.

### Global magnitude of organic carbon burial in seagrass sediments

A first-order global OC burial rate in seagrass sediments over contemporary ( ~ 100-year) timescales can be estimated by scaling the area-weighted average from this analysis to the global seagrass extent. However, estimates of global seagrass distribution vary widely, ranging from 148,506 to 600,000 km^2^^44,55,56^, while model-based projections suggest the potential extent could be an order of magnitude higher^57^. While the lower bound is likely to be conservative^56,58^, McKenzie et al.^55^ synthesized expert-held occurrence and spatial polygon data to establish a revised mapped seagrass extent of 160,387–266,562 km^2^, with high confidence in the lower bound and low confidence in the upper bound. We updated this compilation by reviewing country-level coverage and integrating newly published distribution data since McKenzie et al.^55^ (Supplementary Data 1). Based on these country- and regional-level surveys, which capture meadows over a broader ecological domain, we present an alternative global mapped area ranging between 247,700 and 366,400 km^2^. This updated estimate narrows the range of uncertainty while acknowledging gaps in coverage, particularly in parts of South and East Asia (which in turn support extensive meadows), the Arabian Peninsula, the Horn of Africa, North and West Africa, and the Baltic region. Except for the Arabian Peninsula, these gaps also extend to sediment OC burial rates.

Using these upper and lower area values and the area-weighted average OC burial rate, we estimate that global OC burial in seagrass sediments could range between 6 to 10 Tg OC yr^−1^ (assuming minimal extent) and 9–15 Tg OC yr^−1^ (assuming maximal extent). When sites with negligible sediment accumulation over the past century were included, the mean global estimate declined further to 4–6 Tg C yr^−1^ for the small area range, and to 5–8 Tg C yr^-1^ for the larger range. Previous global assessments used a broader seagrass area range of 300,000–600,000 km^2^^1,2,16^, which would yield a mean global OC burial rate of 10–20 Tg OC yr^−1^, still significantly lower than earlier estimates of 48–112 Tg OC yr^−1^ ^2,16^.

Earlier estimates of global OC burial rates^2^ incorporated net community production (NCP) estimates, a measure of ecosystem-level carbon balance, under the assumption that excess production was largely buried, or adjusted net primary production values using the average fraction of OC storage derived from available budgets^12^. Seagrass ecosystems tend to be net autotrophic, generating a large OC surplus, with an estimated NCP of about 119 ± 50 g OC m^−2^ yr^−1^ (36–71 Tg OC yr^−1^ globally)^15^, which is not used by heterotrophs within the meadow but is either buried locally or exported away^12^. Assuming that roughly half of the total OC buried in seagrass sediments is from seagrass material^16^, the corresponding burial rate of seagrass-derived OC would be 17 ± 4 g OC m^−2^ yr^−1^, suggesting that ~ 15% of the NCP is retained and buried in situ within meadows, while the remaining ~ 85% may be redistributed to adjacent shorelines, unvegetated sediments, and offshore areas^54,59–61^, where it could be buried, remain dissolved, or return to the atmosphere as CO_2_.

Duarte and Krause-Jensen^54^ synthesized existing estimates of earlier OC burial rates and seagrass ecosystem OC balance to show that about 24 Tg yr^−1^ of exported seagrass OC was sequestered beyond the meadow in unvegetated sediments or the deep sea. This exported fraction represents a substantial portion of the NCP not buried in situ and suggests that about 60% of this unburied OC could ultimately be sequesterd beyond meadows. While earlier indirect carbon mass balance assessments by Duarte and Cebrián^12^ already suggested a modest burial and significant export of seagrass production, our analysis reinforces and refines this view with updated burial estimates, further supporting the contention that the magnitude of exported and redistributed seagrass NCP is larger than previously recognized.

Seagrasses are the most widespread among blue carbon ecosystems^54,61,62^, thus their contribution to the total OC burial in vegetated coastal sediments is large and accounts for ~30%, globally (Supplementary Table S5). The total marine OC burial was revised by Duarte et al.^1^ by adding the yet unaccounted contribution of seagrasses, mangroves, and tidal marshes to the long-standing estimates of marine OC burial^62,63^. We revisited this estimate with updated values of OC burial rates in seagrasses, mangroves^4^ and tidal marshes^3^, as well as in the continental margin^64^ and deep sea^65^. Based on this approximation, we estimate that the OC burial within vegetated coastal sediments, including mangroves, tidal marshes, and seagrasses, accounts for ~ 8–13% of the total oceanic OC burial, with burial in seagrass sediments representing between 3-4% using the mapped seagrass area, or 3–6% assuming a 300–600·10^3 ^km^2^ area range; a sizeable but more modest contribution than previously assessed ( ~ 10–18%)^1^ based on limited data more than a decade ago.

### Constraints on interpreting OC burial rates

Challenges and pitfalls of utilizing OC burial rate estimates in blue carbon ecosystems for climate change mitigation have been described elsewhere^23,66^, with long-term permanence and the attribution of OC source, particularly the treatment of allochthonous inputs, as dominant topics of debate^67–69^. These discussions highlight the necessity of clarifying the interpretation and appropriate application of the global estimates presented here. The rates reported in this study represent data-constrained estimates of total OC burial in seagrass sediments integrated over a centennial timescale. They do not provide a direct measure of climate mitigation; rather, they are reference values describing contemporary OC burial within seagrass meadows. As such, they can serve as baseline conditions prior to habitat degradation, or as target conditions following restoration when site-specific measurements are unavailable. Consequently, these estimates are best suited for global carbon-cycle studies and IPCC Tier 1 default factors, rather than for representing processes at local scales, where the large spatial variability in OC burial rates remains insufficiently constrained (Supplementary Fig. S7). Patterns across bioregions and geomorphic settings remain poorly resolved even within well sampled regions (Fig. 2), with site-level variability, expressed as the coefficient of variation, averaging ~ 50% of the mean (Supplementary Fig. S7).

If OC burial rates are used in the context of climate mitigation, the primary consideration is the timescale of interest. Within the contemporary carbon cycle, the climate-relevant metric is the net change in burial fluxes resulting from ecosystem degradation, conservation, or restoration, rather than the baseline absolute OC burial rates reported here. If the objective is to quantify long-term carbon preservation over centuries to millennia, accounting for the remineralization of labile surface OC and long-term sediment accumulation rates become more relevant. These long-term dynamics can be constrained by assessing stabilized OC concentrations at depth^70^ alongside with longer-term chronologies (e.g., ^14^C). However, such approaches raise additional questions regarding historical environmental conditions, including whether seagrass ecosystems were present during that period.

Sediment OC source attribution is commonly based on stable isotope mixing models, as seagrass δ¹³C is typically enriched relative to terrestrial, mangrove, salt marsh, and plankton organic matter sources^71,72^. However, inferred contributions are highly dependent on endmember selection, and emerging approaches such as compound-specific isotopes and eDNA offer improved source resolution^73^. It is important to clarify that the distinction between autochthonous and allochthonous OC refers to within-ecosystem production versus external inputs, rather than a simple seagrass versus non-seagrass dichotomy. Accordingly, both seagrass and other in situ primary producers (e.g., micro- and macroalgae) are autochthonous, whereas terrestrial, mangrove, and salt-marsh inputs are allochthonous. The OC burial rates reported here represent total sediment OC burial, integrating all sources and representing the net outcome of OC gains and losses over a century scale. The partitioning of autochthonous versus allochthonous OC remains debated but is not required under major accounting frameworks. For instance, IPCC greenhouse gas inventory guidelines and wetland restoration assessments typically rely on total OC burial without requiring source partitioning. Consequently, under accounting frameworks based on net additional storage, resolution of source attribution and long-term OC cycling processes may be secondary to quantifying the net change in OC burial resulting from management actions. Ultimately, the relevance of these OC burial rates depends on the specific biogeochemical, carbon cycle, or policy context in which they are applied.

### Outlook

Our results support a substantial downward revision of OC burial rates in seagrass sediments to 33 ± 9 g OC m^−2^ yr^−1^ (or 6–15 Tg OC yr^−1^ globally, based on mapped area), providing a more accurate baseline of the OC burial capacity of seagrasses over a centennial time scale. While this OC burial rate accounts for 3–4% of total marine OC burial, the significance of seagrass ecosystems as global carbon sinks may be larger. We estimate that seagrass meadows export roughly three times more OC than what is buried locally, highlighting the importance of quantifying and constraining the fate of this exported fraction to resolve global estimates of seagrass contributions to oceanic OC storage. Moreover, since the total seagrass area may be larger than that quantified here and there has been extensive historical losses of seagrass meadows^74^, it is likely that past contributions to global OC burial would have been higher, pointing to a potential for climate change mitigation by avoiding further losses and restoring seagrass ecosystems to their past extent^74,75^^.^

The relationship between sediment accumulation rates and sedimentary OC content can help delineate burial patterns, identifying sites with enhanced OC burial potential. Overall, the presence of seagrass is associated with more frequent depositional conditions conducive to OC burial, whereas non-depositional settings are more commonly observed in bare, unvegetated sediments. Within depositional settings, both vegetated and bare sediments could exhibit comparable OC burial dynamics. However, seagrass meadows remain critical for sustaining regional OC pools over time. Their loss could not only limit local production but also compromise landscape-scale sedimentary OC storage and redistribution of organic matter, ultimately diminishing long-term coastal and oceanic carbon sequestration.

## Methods

### Data extraction and processing

We compiled data on direct estimates of OC burial rates over the past century based on ^210^Pb, in seagrass-vegetated and adjacent unvegetated sediments, drawing from published articles, gray literature, and unpublished contributions collected over several years. We excluded studies relying on timescales outside the century-scale ^210^Pb resolution, such as long-term radiocarbon (^14^C) or short-term (months to years; based on surface elevation tables or marker horizons) measurements. Studies were also excluded if they lacked ^210^Pb specific activity profile data. Additionally, we excluded any sites identified as degraded, disturbed or restored in the primary literature, as their OC burial rates might be affected by a shifting baseline or the time since planting, which varies between sites and does not align with the temporal resolution over which OC burial rates were assessed in the rest of the dataset’s cores. The complete list of included studies and references is available in the 'associated_publications' bibliography file within the dataset repository^31^.

We compiled quantitative parameters such as ^210^Pb specific activity profiles, derived mass accumulation rates (MAR; g cm^−2^ yr^−1^) or sedimentation rates (SAR; cm yr^−1^), sedimentary organic matter (OM) and OC content (%), and sediment dry bulk density (DBD). An effort was made to record core mass depths (g cm^−2^) at each segmented interval alongside DBD and other measured variables; otherwise, mean values were recorded instead. Where available, ancillary data were recorded, including site or core coordinates, seagrass species and genera, δ^13^C signatures of seagrass and sediments, and sediment grain size. Compaction of sediments may occur during coring and affect depth, DBD and vertical accretion rates (SAR). Where reported, we also recorded evidence of compaction and noted whether sedimentation rates had been corrected accordingly. Core locations were categorized into coastal geomorphological types based on their latitude and longitude using the global classification scheme by Dürr et al.^33^. Each core was assigned to one of the following categories: small deltas (I), tidal systems (II), lagoons (III), fjords (IV), karstic coasts (VI), or arheic (dry) coasts (VII). Glaciated coasts (0) and large rivers (V) were not represented in the dataset.

In addition to vegetated seagrass cores, we also included unvegetated sediments (*n* = 37 cores) sampled alongside seagrass meadows in the reviewed studies. These were treated separately but classified and analyzed using the same criteria and methods. At sites where paired data were available (16 sites, of which 8 exhibited net positive sediment accumulation over the past century), we conducted paired comparisons on sedimentary variables and sediment and OC burial rates between vegetated and unvegetated cores.

The full dataset comprised 332 cores from 65 studies, including 47 published articles, 3 theses and 3 technical reports, and 12 unpublished datasets^31^. In contrast to recent reassessments of OC burial rates in tidal marshes^3,25^ and mangroves^4,26^, we also considered sites where intense downcore mixing or negligible excess ^210^Pb accumulation precluded the determination of contemporary OC burial rates.

### Data curation

We assigned ^210^Pb profile quality flags to all cores in the database for quality control purposes, classifying each profile as (1) ideal, (2) exhibiting surface mixing, (3) pervasive downcore mixing (affecting > ½ of the datable part of the profile), or (4) negligible excess ^210^Pb following Arias-Ortiz et al.^28^. Cores exhibiting intense downcore mixing (quality flag 3) for which OC burial rates were reported (26% of cores) were included in the main analyses. However, global burial rates were computed with and without these values to assess the potential influence of ^210^Pb profile quality on OC burial estimates, particularly the extent to which downcore mixing may bias rates high. Sites exhibiting intense mixing where OC burial rates could not be resolved were retained in the dataset, but rates were recorded as NA.

When available, we used published OC burial rates. For studies where rates were reported for specific depth intervals, dates predating the excess ^210^Pb horizon, or where only radiometric dates (but not accumulation rates) were provided, we recalculated them to represent the mean OC burial rate over the past century using the full-dated profile. To minimize the effects of possible core compaction, we recalculated OC burial rates as the product of the fraction of OC and the MAR Eq. (1).

1 $${\rm{OC}}\; {\rm{burial}}\; {\rm{rate}}={\rm{OC}}_{\rm{t}}{\rm{\cdot }}{\rm{MAR}}_{\rm{t}}$$

where ${{\rm{OC}}}_{{\rm{t}}}$ is the mean OC concentration of the sediments accumulated within the excess ^210^Pb-dated interval (i.e., from the sediment surface to the depth where excess ^210^Pb reaches background levels), and MARt is the mean mass accumulation rate over the same region.

We compared published OC burial rates with first-order recalculated estimates Eq. (1) and used standardized residuals ( > |2 | ) to identify unusually large discrepancies. Where these discrepancies could not be explained by methodological differences (e.g., time-specific versus centennial-scale estimates), we retained the recalculated rates to ensure methodological consistency across the dataset. At sites where total ^210^Pb specific activities reflected only the background (supported levels of ^210^Pb), suggesting no net sediment accumulation (18% of cores), we assumed that the net OC burial over the last century was negligible and not significantly different from zero. By factoring these sites into our analysis, we estimated a lower bound for areal OC burial in seagrass sediments that is less biased toward depositional environments. Published, calculated, and used OC burial rates can be found in the summary_results_table accompanying the dataset in Arias-Ortiz et al.^31^. Methods documentation such as ^210^Pb quality flags, compaction percentages, and OC analytical methods are provided in the 'depthseries' and 'methods' dataset tables^31^.

Ten studies (56 cores) estimated OC from organic matter (OM) content using local site-specific regressions, and four studies used the general equation developed by Fourqurean et al. Equation (2)^18^. In nine cores sampled from the banks of Florida Bay^40^, OC content was neither measured nor estimated, although OM content was provided. A site-specific equation Eq. (3) published for Florida Bay^76^ was applied to these sites.

2 $${\rm{OC}}\left(\%\right)=0.43.{\rm{\cdot OM}}\left(\%\right)-0.33$$

3 $${\rm{OC}}\left(\%\right)=0.54.{\rm{\cdot OM}}\left(\%\right)-0.99$$

Sediment δ^13^C values were used in conjunction with seagrass δ^13^C signatures to assess the influence of seagrass organic matter on sedimentary OC^16^. Seagrass δ^13^C signatures were assigned to each core using the most site-specific information available. Where species-specific δ^13^C values were reported at the site, these were matched to the seagrass species present at the core location, otherwise, site-level mean seagrass δ^13^C values were used, which could include multiple species. Mean site-level seagrass δ^13^C values were also assigned to unvegetated cores collected within the same site. Bare sediment cores that did not share a Site ID with vegetated cores were not assigned a seagrass δ^13^C endmember (13 out of 37). Because seagrass δ^13^C signatures are isotopically heavier than those of other coastal organic matter sources (e.g., terrestrial vegetation, mangroves, tidal marshes, and plankton)^71,72^, the difference between seagrass and sediment δ¹³C values (Δ^13^C = δ^13^C_seagrass_ − δ^13^C_sediment_) was used as a proxy for the presence of seagrass versus non-seagrass sediment OM. This approach was chosen because non-seagrass endmembers and their classification as internal or external sources to the ecosystem were not consistently reported across studies, and terrestrial allochthonous inputs were not always sampled, precluding cross-study comparisons and motivating the Δ^13^C harmonized approach.

Recent studies have suggested using stabilized OC concentrations at depth to minimize the effect of labile OC that has not undergone decomposition in burial rate estimates^23,70^. To evaluate this effect across the dataset, we examined sediment OC profiles from all seagrass cores for which OC burial rates could be derived. For each core, we fitted a linear regression between OC concentration and depth over the ^210^Pb-derived chronostratigraphic interval and classified profiles based on the sign of the slope and *p*-value (α < 0.05). As a result, 51% of profiles exhibited stable OC concentrations with depth, 39% showed decreasing OC concentrations, and 10% displayed increasing OC content with depth, suggesting that although some OC decomposition occurs on centennial timescales, the magnitude of the resulting decline varies strongly among seagrass meadows.

To quantify the magnitude of downcore OC change, we calculated the ratio between mean OC concentration in the bottom 3 cm and the uppermost 3 cm of each profile within the dated layer. Decreasing OC profiles exhibited a mean bottom-to-top OC ratio of 0.44. In contrast, increasing profiles exhibited a ratio of 1.82, while stable OC profiles showed a ratio close to unity (0.90). When all vegetated sediments were pooled together, the median bottom-to-top OC factor was 0.70. Because the observed downcore OC decline varied considerably among sites, we did not apply a remineralization factor to the global OC burial rate estimates. If applied, it would yield rates similar to our most conservative estimate, which includes sites with negligible recent accumulation (20 g OC m^−2^ yr^−1^; 95% CI: 15–23).

Based on the downcore OC profile distributions, the vast majority of compiled cores ( ~ 90%) displayed stable concentrations with depth or natural diagenetic decay rather than systematic seagrass disturbance. However, because establishing pre-industrial reference baselines requires longer-term historical records, we did not analyze the potential for a shifting baseline in OC burial rates driven by increased human impact during the last century. Future studies are needed to quantify global change impacts on OC burial and decipher the fate and permanence of seagrass sediment carbon.

### Global OC burial rate estimation and area scaling

Global OC burial rates per unit area were estimated using two approaches. First, we calculated the geometric mean of OC burial rates for vegetated sites with net positive sediment accumulation, and the median across all vegetated sites, including those with negligible accumulation, as these metrics best represent the central tendency. Second, an area-weighted average was calculated using the relative contribution of each seagrass bioregion to the total seagrass extent. For the latter calculation, estimates of OC burial rates were grouped by seagrass bioregions described in Short et al.^32^ (i.e., Temperate North Atlantic, Temperate North Pacific, Mediterranean, Tropical Atlantic, Tropical Indo-Pacific, and Temperate Southern Oceans). In our analysis, the Tropical Atlantic is represented primarily by the Tropical Western Atlantic, with only two cores available for the eastern side of this bioregion. Similarly, estimates for the Temperate Southern Oceans are largely derived from Australian sites, reflecting limited data availability from other parts of the Southern Hemisphere. Then, the geometric mean OC burial rates per unit area were estimated for each bioregion, and globally as an area-weighted average based on the relative contribution of each bioregion to the total global seagrass extent. The relative area contribution for each bioregion was calculated by dividing the reported spatial extent of that bioregion by the total global seagrass extent (Supplementary Data 1). Because global lower and upper seagrass area bounds were calculated by summing the respective lower and upper area estimates across all bioregions, these regional extent proportions remain similar and were applied as constant percentage weights in the global area-weighted average. Cores with negligible sediment accumulation were excluded from the weighted average estimate because such observations are not reported as consistently as those with net positive accumulation, often due to perceived ^210^Pb dating failures. Including these sites would disproportionately penalize bioregions where authors more frequently report lack of accumulation based on ^210^Pb dating. Moreover, previous global syntheses have not incorporated these sites, ensuring consistency for comparative purposes. Nevertheless, for global summary statistics across the full dataset, we consider it important to include sites with little-to-no sedimentation, as negligible excess ^210^Pb is itself a meaningful result that may reflect sediment dynamics rather than merely methodological limitations, and its reporting should be encouraged.

Over a 3-fold bracket exists in the global area reported for seagrass meadows (148,506 − 600,000 km^2^)^44,55,56^. The lower estimate is based on the recent pioneering work by Peng et al.^56^ using a deep-learning workflow across a Sentinel-2 observable domain in clear, shallow waters ( < 20–30 m) within 5 km of the shore. While this represents the first comprehensive, 10m-resolution global seagrass map, it remains a conservative estimate due to optical depth and turbidity constraints. For this study, we revised the mapped seagrass area in McKenzie et al.^55^ based on available polygon data and expert held occurrences, by incorporating recent country- and regional-level seagrass areas estimates derived from multiple survey and mapping approaches and intended to represent the broader ecological distribution of seagrasses (Supplementary Data 1). For countries where updated seagrass area estimates were available, we subtracted the previously assigned areas in McKenzie et al.^55^ and added the new figures. These updates resulted in a revised mapped seagrass area range of 247,700–366,400 km². A substantial portion of the increase from the earlier range reported by McKenzie et al.^55^ (160,387–266,562 km^2^) is attributable to the recent mapping of extensive ( > 57,000 km^2^) seagrass meadows in The Bahamas^77^, and in the Insular Caribbean^78^ among other additions. Previous assessments of OC burial rates in seagrass meadows commonly assumed a global extent of 300,000 − 600,000 km^2^^1,2,16^. To enable comparison with these studies, we also upscaled OC burial rates using this range, alongside our updated mapped seagrass extent.

### Statistical analysis

We analyzed the distribution, skewness, and kurtosis of the data. A Shapiro-Wilk test provided an indication of normality for the untransformed and transformed versions of the dataset. These results were used to assess which parameter (the arithmetic mean, median, or geometric mean) was best suited to represent the central tendency of our data. The arithmetic mean was used if data followed a normal distribution, the geometric mean if a skewed distribution was made symmetrical by a log transformation, and the median if either the untransformed or log-transformed dataset did not follow a normal distribution. Non-parametric tests were generally used for statistical comparisons, as most parameters did not follow a normal distribution. Paired two-group comparisons (e.g., between vegetated and adjacent unvegetated sites) were assessed using the paired-sample Wilcoxon signed-rank test, while differences across multiple groups (e.g., among bioregions, coastal typologies, or seagrass genera) were tested using the Kruskal–Wallis test followed by Dunn’s post hoc test with Bonferroni correction. All statistical analyses were done at a level of significance of α < 0.05. To examine controls on OC burial, we analyzed relationships between sediment OC content, MAR, and OC burial rates across vegetated sites. Burial rates were grouped into quantile intervals (low: 0–25%, intermediate: 25–75%, high: 75–100%) to identify thresholds associated with contrasting depositional settings. Similarly, MAR were grouped into the same quantile intervals. Within each interval, log–log regressions were used to assess how OC burial scales with OC content, and the resulting slopes were compared to interpret sediment OC preservation under varying sedimentation rates.

## Supplementary information

Supplementary Information

Description of Additional Supplementary File

Supplementary Dataset 1

Transparent Peer Review file

## Data Availability

All sediment profile data and synthesized organic carbon burial rates compiled in this study have been deposited in the Smithsonian’s Figshare data repository and are available in Arias-Ortiz et al.^31^
