# Supplementary material for: Seagrass sediment organic carbon burial rates are globally significant
Source: Nat Commun. 2026 Nov 4;17:11494. doi: 10.1038/s41467-026-79168-3 (PMC13743778; doi:10.1038/s41467-026-79168-3)
Supplement: Supplementary file 1 [file 41467_2026_79168_MOESM1_ESM.pdf]

Supplementary Information for:

Seagrass sediment organic carbon burial rates are globally significant

Ariane Arias-Ortiz[1*], Anna Lafratta[2], Phil Colarusso[3], James W. Fourqurean[4,5], Chuancheng Fu[6], Hilary Kennedy[7], Johannes R. Krause[4], Paul S. Lavery[2], Carmen Leiva-Dueñas[8], Nuria Marbà[9], Miguel A. Mateo[2,10], Inés Mazarrasa[11], Dimitris Poursanidis[12], Cristian Salinas[2], Oscar Serrano[10], Milica Stankovic[13, 14], Nerea Piñeiro-Juncal[15], Axel Werner[2], Marlene Wesselmann[2], Carlos M. Duarte[6], Jordi Garcia-Orellana[1], Pere Masqué[2]

[1]Department de Física, Universitat Autonoma de Barcelona, Bellaterra, 08193 Barcelona, Spain

[2]School of Science & Centre for Marine Ecosystems Research, Edith Cowan University, Joondalup WA 6027, Australia.

[3]United States Environmental Protection Agency, Boston, Massachusetts 02109, USA.

[4] Institute of Environment, Florida International University, 11200 SW 8th St, Miami, FL 33199, USA

[5] Department of Biological Sciences, Florida International University, 11200 SW 8th St, Miami, FL 33199, USA

[6] Marine Science Program, Biological and Environmental Science and Engineering Division (BESE), King Abdullah University of Science and Technology (KAUST), Thuwal 23955-6900, Saudi Arabia.

[7]School of Ocean Sciences, Bangor University, Menai Bridge, Wales, UK

[8]Department of Ecoscience, Aarhus University, C.F. Møllers Allé, building 1131, DK-8000 Aarhus, Denmark

[9]Global Change Research Group, IMEDEA (CSIC-UIB) Institut Mediterrani d'Estudis Avançats, Miquel Marqués 21, 07190 Esporles, (Spain)

[10]Centre for Advanced Studies of Blanes, Spanish Council for Scientific Research, 17300 Blanes, Spain.

[11]IH Cantabria, Instituto de Hidráulica Ambiental de la Universidad de Cantabria C/. Isabel Torres, 15, 39011 Santander, Cantabria

[12] Foundation for Research and Technology—Hellas (FORTH), Institute of Applied and Computational Mathematics, N. Plastira 100, Vassilika Vouton, 70013 Heraklion, Greece

[13]Excellenece Center for Biodiversity of Peninsular Thailand, Faculty of Science, Prince of Songkla University, Hat Yai, Songkhla 90110, Thailand

[14]Dugong and Seagrass Research Station, Faculty of Science,Prince of Songkla University, Hat Yai, Songkhla, 90110, Thailand

[15]Centro de Investigacions Mariñas, Departamento de Xeocencias Mariñas e Ordenación do Territorio, Universidad de Vigo, Vigo, Spain.

Correspondence: Ariane Arias-Ortiz (ariane.arias@uab.cat)

Figures

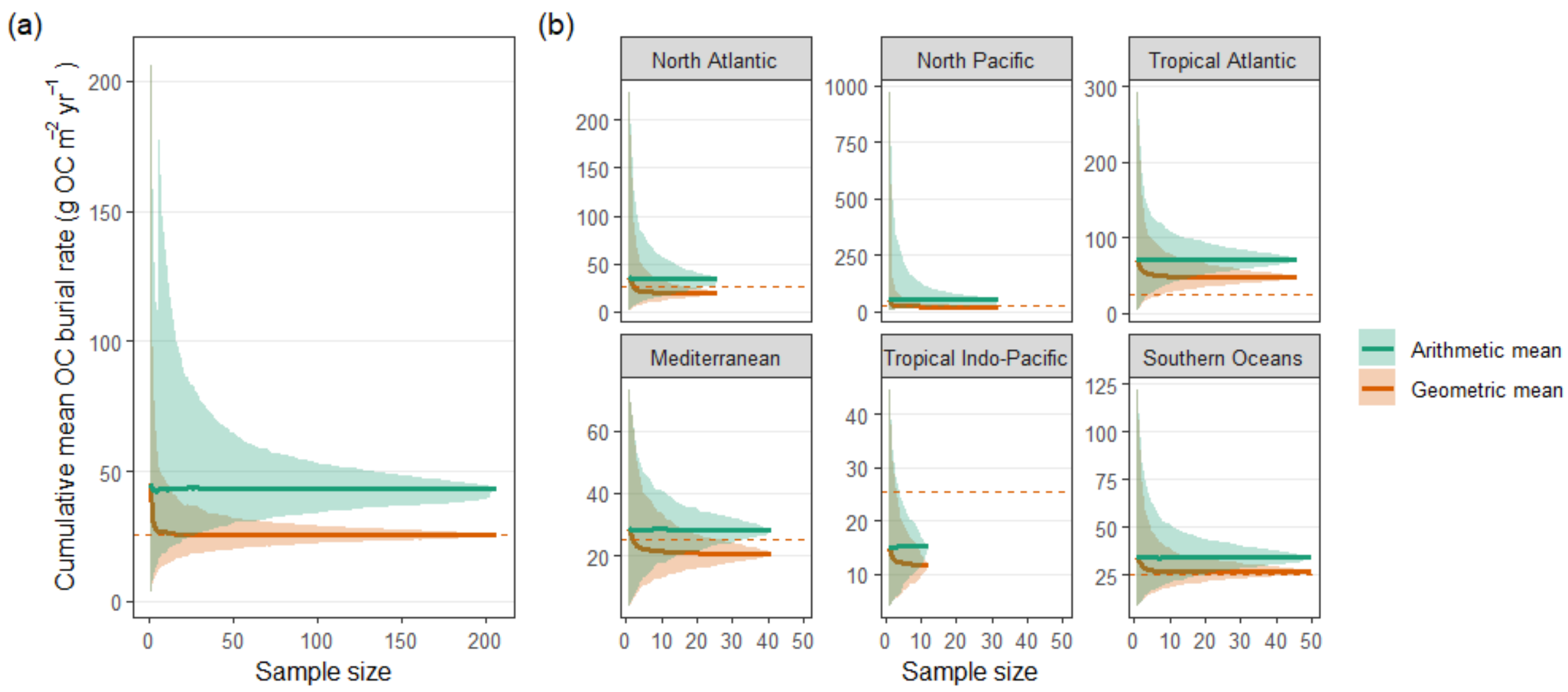

Figure S1. Stability of estimated organic carbon burial rates under increasing number of OC burial rate estimates. (a) Cumulative arithmetic and geometric means of OC burial rate as a function of sample size, based on 1,000 random permutations of all vegetated cores with net accumulation combined; (b) regional subsets. Shaded areas represent 95% confidence intervals. The dashed line indicates the geometric mean OC burial rate across vegetated sites (25 g OC m^{-2} yr^{-1}). Note change in y-axis across panels.

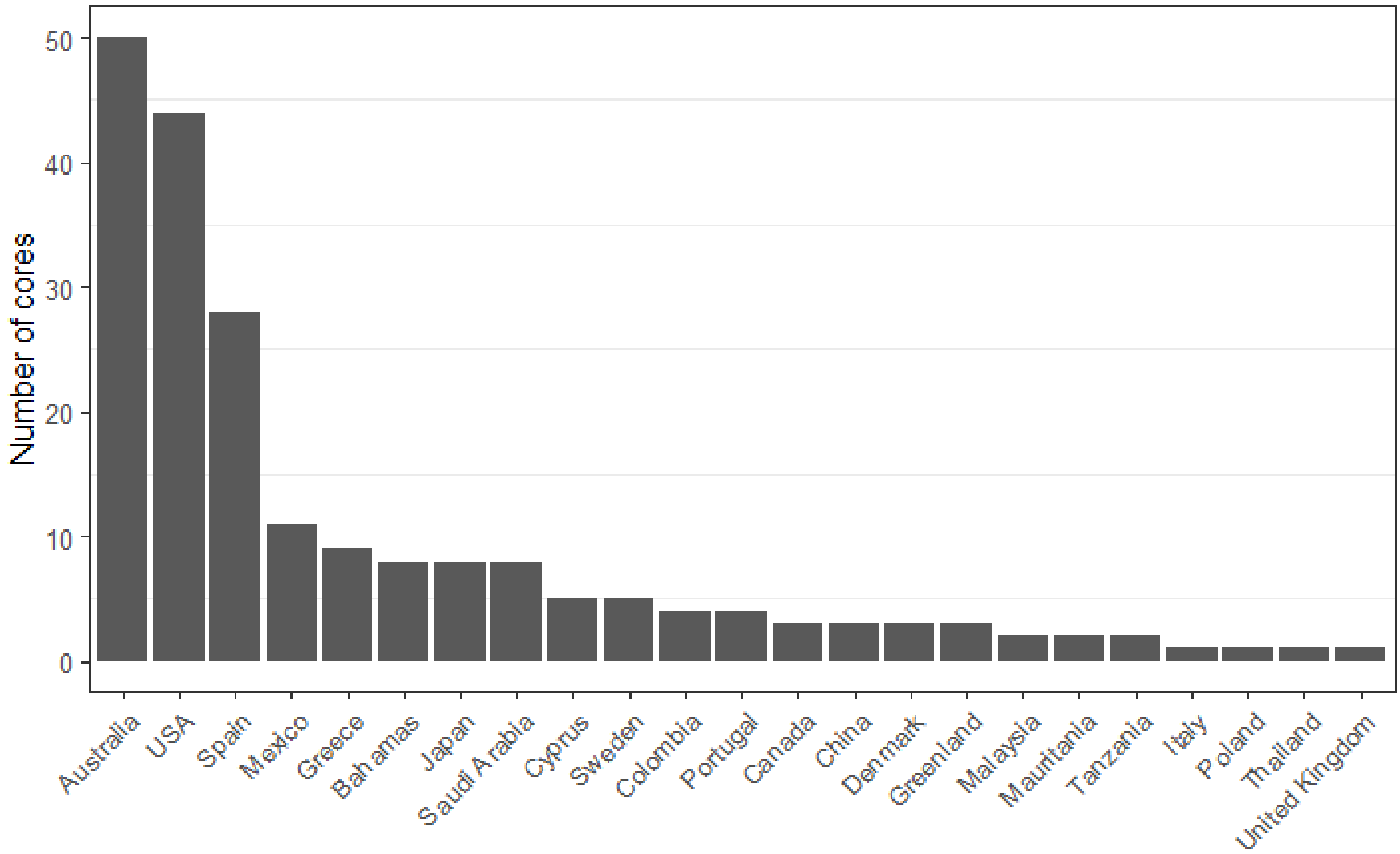

Figure S2. Number of cores per country in dataset.

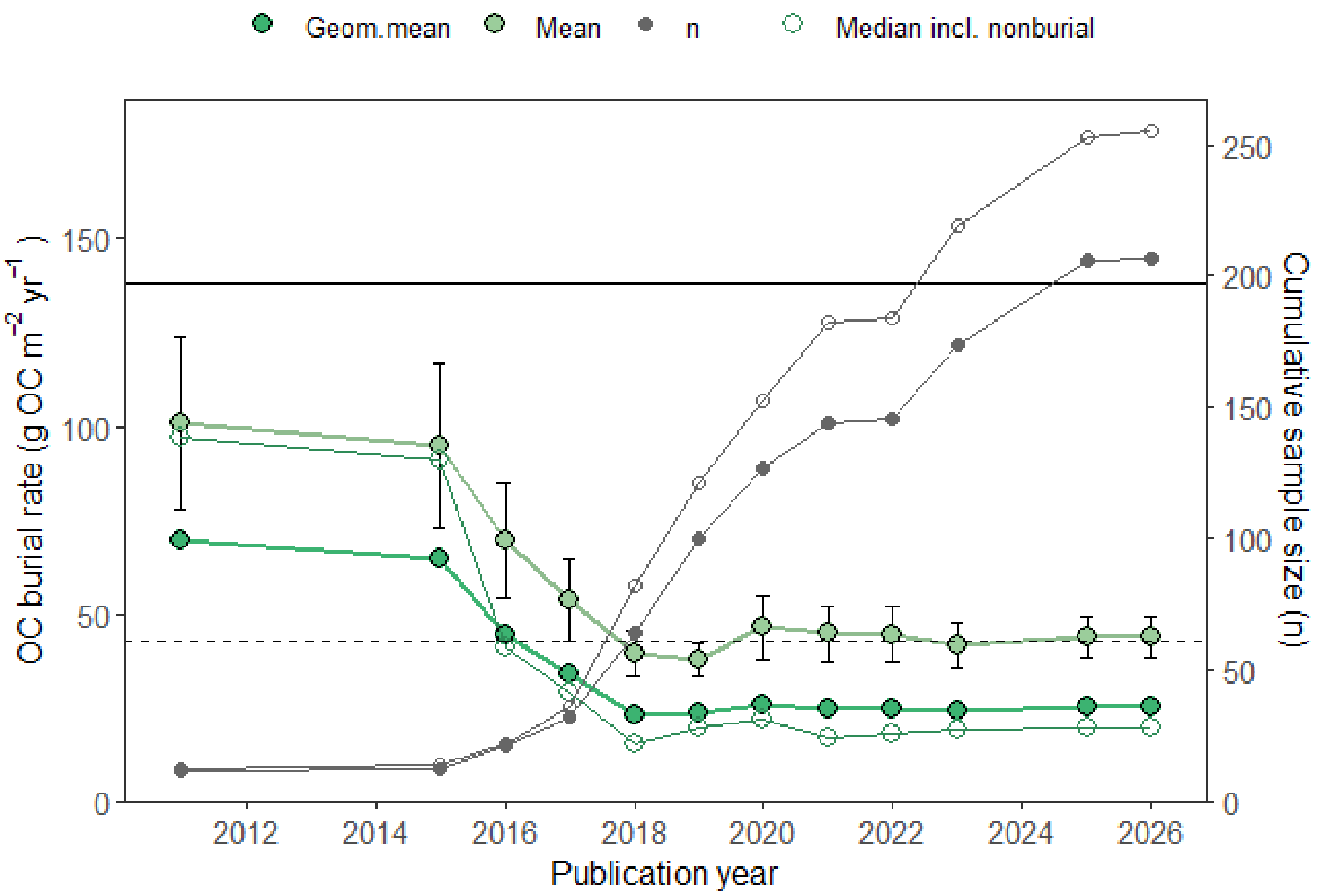

Figure S3. Summary statistics for organic carbon (OC) burial rates in seagrass sediments by publication year. Solid symbols represent statistics for seagrass cores showing net positive accumulation, while open symbols include the same statistics for both depositional and non-depositional seagrass sediments. Horizontal lines indicate the IPCC Tier 1 estimate (dashed) and the mean OC burial rate reported by McLeod et al. (2011) (solid).

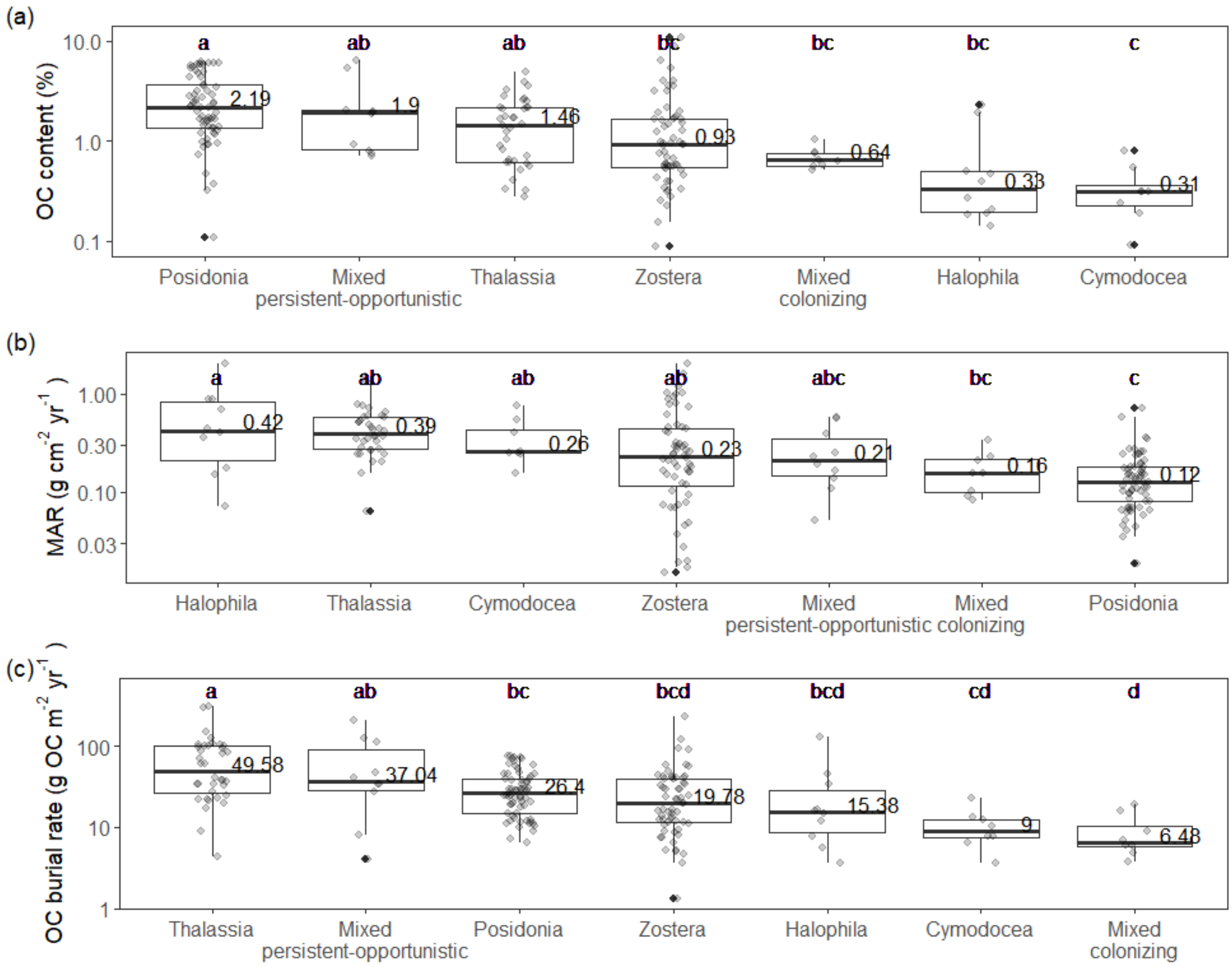

Figure S4. (a) Sediment organic carbon (OC) content in the dated sediment column, (b) mass accumulation rates (c) and OC burial rates grouped by seagrass genus. Genus containing less than 5 observations are not shown. Boxes show the interquartile range, horizontal lines indicate the median, and whiskers extend to 1.5 times the IQR, individual data points are overlaid. Solid dark data points represent outliers and values on the box plot represent median values. Mixed persistent-opportunistic records included combinations of species of the genera *Posidonia* and *Amphibolis, Thalassia* and *Syringodium*, or *Thalassia* and *Cymodocea*. Mixed colonizing records referred to cases where species from the genera *Halodule, Ruppia,* or *Halophila* were reported together. Different lower-case letters represent significant differences between coastal geomorphologies as a result of a Kruskal–Wallis Dunn's multiple comparison test with $p < 0.05$ adjusted using the Bonferroni procedure ($p < \alpha/2$).

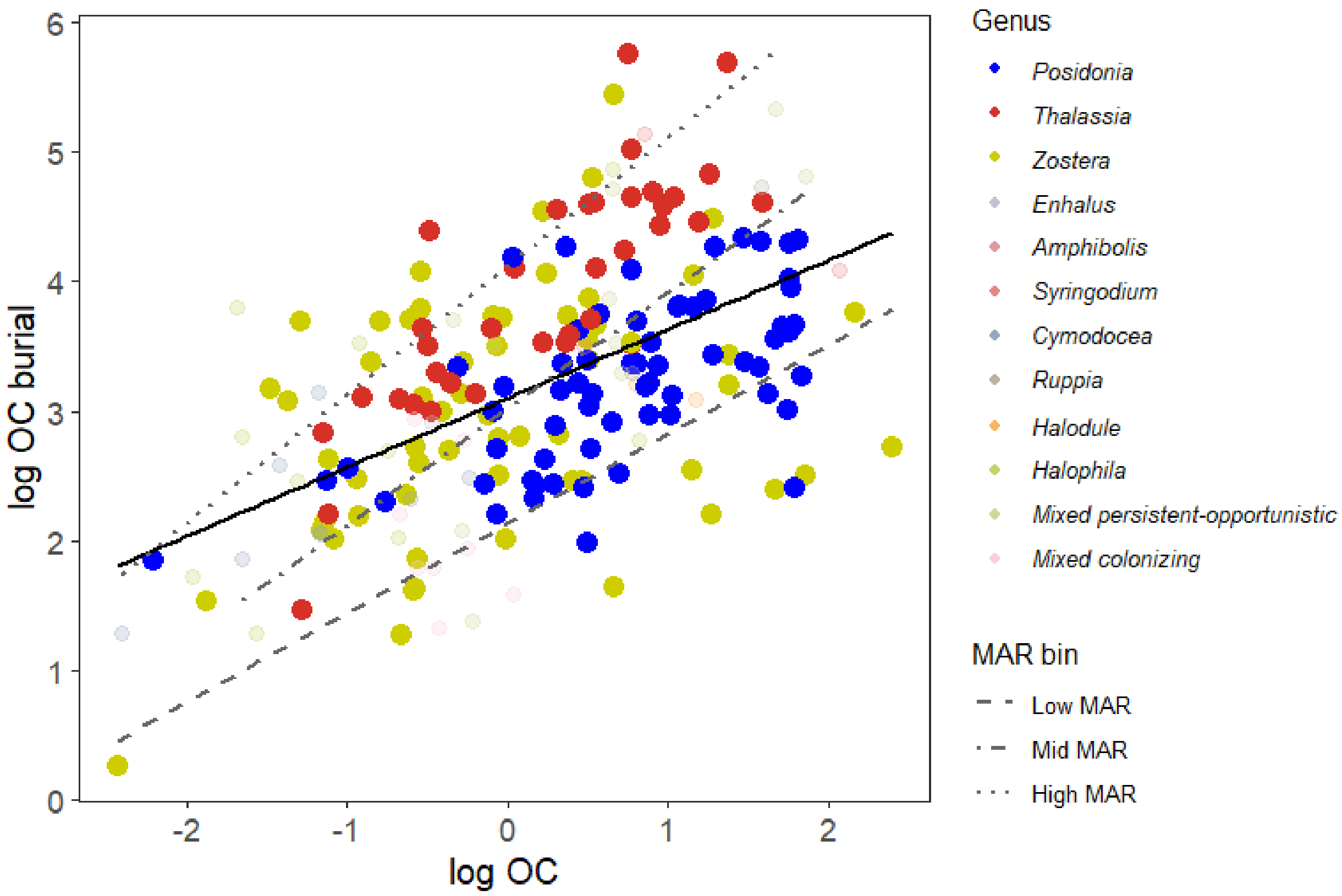

Figure S5. Log-log relationship of organic carbon (OC) burial and OC content in vegetated sediments. Individual observations are colored by seagrass genera, with *Posidonia, Thalassia* and *Zostera*, the dominant genus in the dataset colored bright. Mixed persistent-opportunistic records included combinations of species of the genera *Posidonia* and *Amphibolis, Thalassia* and *Syringodium*, or *Thalassia* and *Cymodocea*. Mixed colonizing records referred to cases where species from the genera *Halodule, Ruppia,* or *Halophila* were reported together. Dashed lines represent linear fits for each MAR quantile (low MAR: 0–25%, Mid: 25–75% and High: 75–100%) and the solid line represents the overall mean linear fit across all points (equation for mean fit shown in Fig. 4 in the main manuscript).

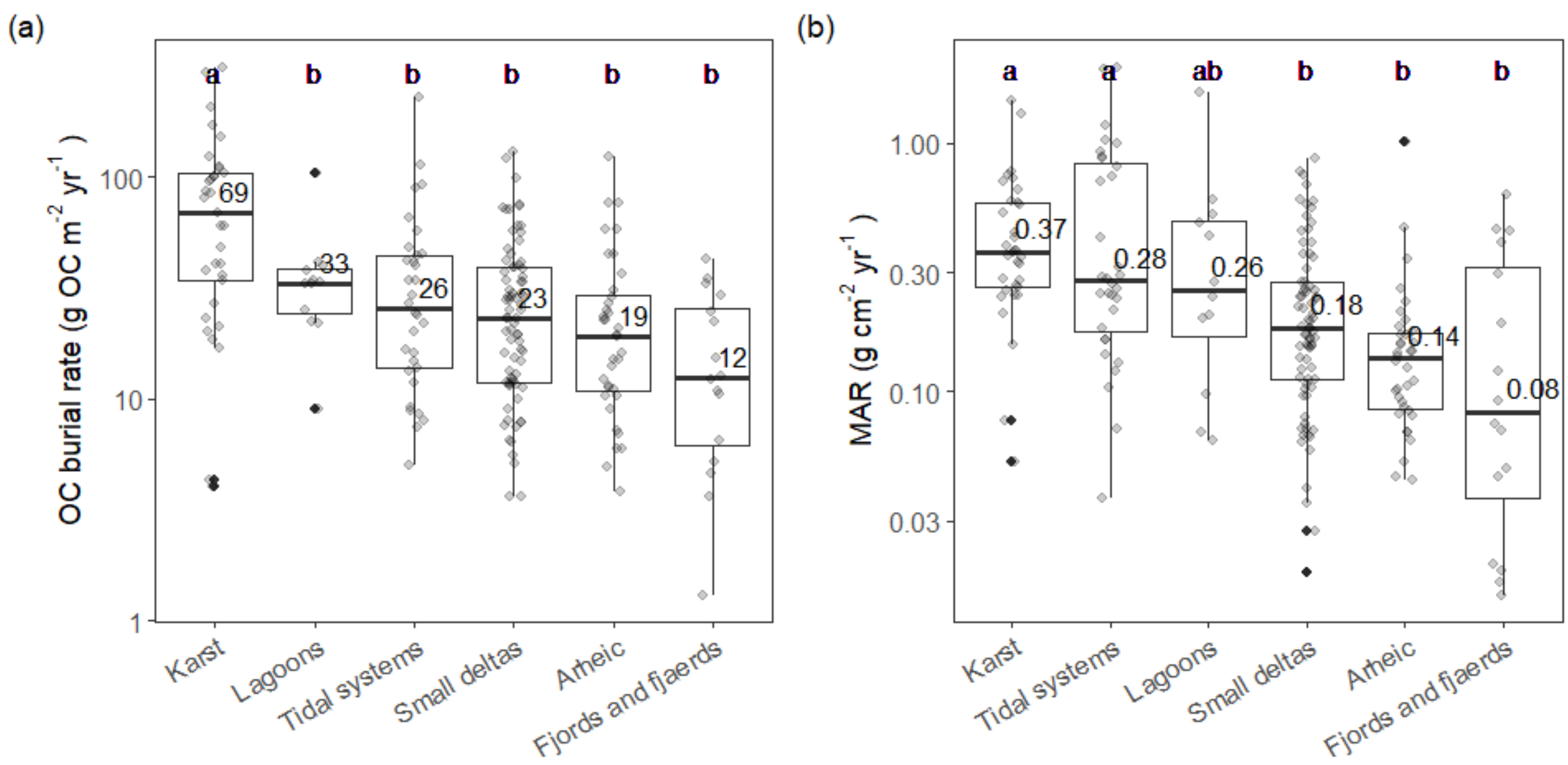

Figure S6. Organic carbon (OC) burial rates (a) and mass accumulation rates (b) in vegetated seagrass sediments with measurable accumulation grouped by coastal geomorphological categories in Dür et al. [1]. Different lower-case letters represent significant differences between coastal geomorphologies as a result of a Kruskal–Wallis Dunn's multiple comparison test with $p < .05$ adjusted using the Bonferroni procedure ($p < \alpha/2$). Values on the box plot represent median values.

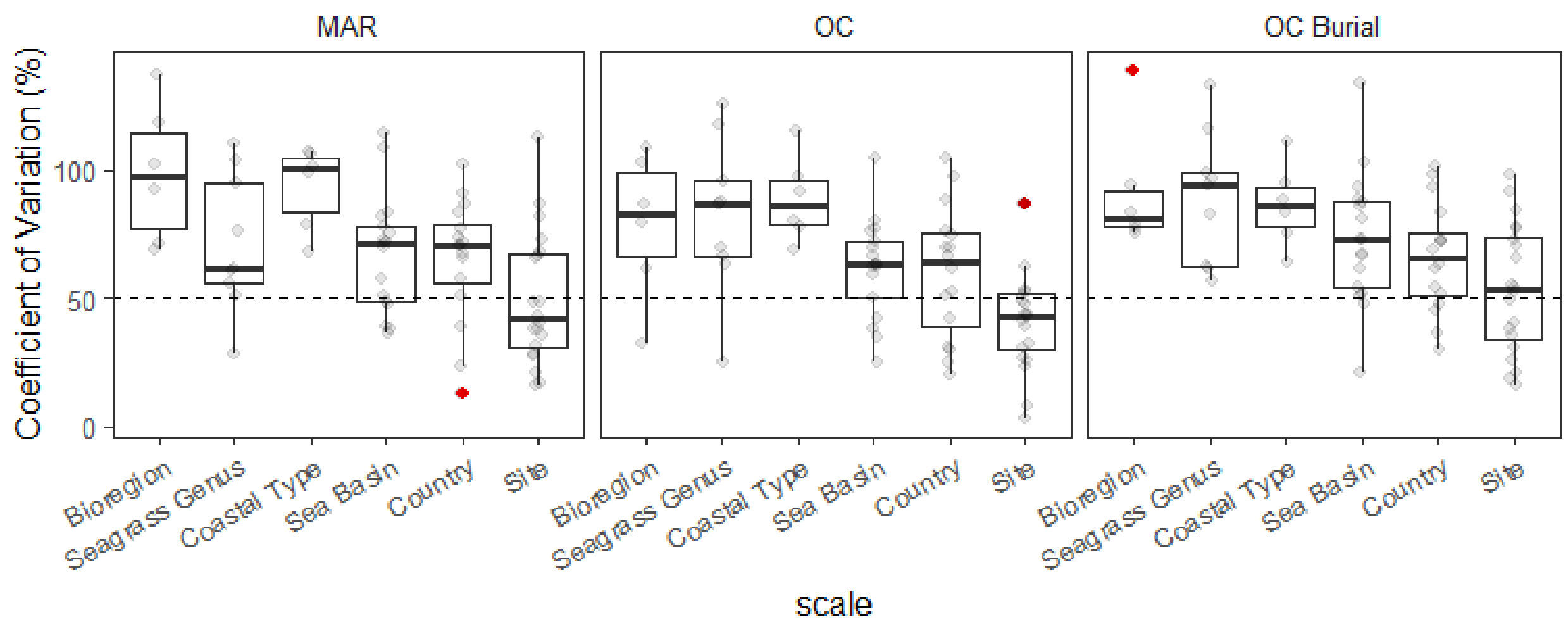

Figure S7. Coefficients of variation in organic carbon (OC) burial rates, mass accumulation rates, and OC content across different spatial scales. Boxes show the interquartile range, horizontal lines indicate the median, and whiskers extend to 1.5 times the IQR, individual data points are overlaid, red data points represent outliers.

Tables

Table S1. Summary statistics for organic carbon burial rates (g OC m^{-2} yr^{-1}) in vegetated seagrass sediments with net positive accumulation.

Dataset Subset	n	Distribution	min	max	mean	SD	median	geom. mean
All	207	log-normal	1.30	976	44	79	25	25
Excluding intense downcore mixing	127	log-normal	3.6	314	42	50	28	27

Table S2. Summary statistics for organic carbon burial rates (g OC m^{-2} yr^{-1}) in vegetated seagrass sediments with net positive accumulation grouped by bioregion. Includes all cores without filtering out those showing intense downcore mixing.

Bioregion	n	Distribution	min	max	mean	SD	median	geom mean	95% C.I.
North Atlantic	26	log-normal	1.30	230	35	48	16	19	13-30
North Pacific	32	non-normal	4.63	976	55	169	22	22	16-33
Tropical Atlantic	46	log-normal	4.00	314	72	68	44	47	36-63
Mediterranean	41	log-normal	3.62	75	28	21	23	21	16-26
Tropical Indo-Pacific	12	log-normal	3.80	45	15	12	13	12	8-18
Southern Oceans	50	log-normal	7.26	130	34	28	26	26	22-32

Table S3. Summary statistics for organic carbon (OC) content, mass accumulation rates (MAR), and OC burial rates in vegetated seagrass sediments with net positive accumulation binned by OC burial rate percentile ranges (0-25%, 25-75%,75-100%).

		**OC (%)**					**MAR (g cm^{-2} yr^{-1})**					**OC burial rate (g C m^{-2} yr^{-1})**				
**OC burial percentile bins**	**n**	**Min**	**Q1**	**Q2**	**Q3**	**Max**	**Min**	**Q1**	**Q2**	**Q3**	**Max**	**Min**	**Q1**	**Q2**	**Q3**	**Max**
Low (0-25%)	52	0.09	0.3	0.6	1.1	6.4	0.02	0.09	0.15	0.25	0.62	1.3	5.9	8.0	11.1	12.3
Intermediate (25-75%)	102	0.19	0.7	1.4	2.2	11.0	0.02	0.10	0.20	0.36	2.04	12.4	19.5	25.0	34.0	42.9
High (75-100%)	52	0.19	1.7	2.6	3.7	7.9	0.08	0.18	0.34	0.69	2.01	44.4	58.4	76.7	105.5	314.0

Table S4. Parameters and their standard errors and statistics for the fitted quantile regression equations between organic carbon (OC) content and sediment mass accumulation rates (MAR), and OC content and OC burial rate in Figure 4a and 4b, respectively.

Function	Percentile bin	Intercept ± SE (a)	Slope ± SE (b)	p-value
	0-25	6.2 ± 0.9	-22 ± 4	<0.001
OC% = a·exp(b·MAR)	25-75	9.8 ± 0.8	-12 ± 1	<0.0001
	75-100	5.8 ± 0.6	-1.9 ± 0.3	<0.0001
	0-25	2.14 ± 0.09	0.69 ± 0.07	<0.0001
logOC burial = a + b·logOC	25-75	3.02 ± 0.04	0.90 ± 0.05	<0.0001
	75-100	4.12 ± 0.06	0.99 ± 0.06	<0.0001

Table S5. Estimates of organic carbon (OC) burial rates in vegetated coastal habitats, and their relative contribution to OC burial in both the coastal and global ocean. Modified from Duarte et al.[2], incorporating updated mapped area estimates[3–5] and revised areal organic carbon burial rates for vegetated coastal ecosystem[6,7] and updated global rates for the continental margin[8], and the deep sea[9].

Compartment	Area extent mapped 10^3 km^2	OC Burial g OC m^{-2} yr^{-1}		Global OC burial Tg OC yr^{-1}	
Vegetated coastal habitats					
Mangroves	82[3]-146[4]	139	± 18	11 ± 2	20 ± 3
Tidal Marsh	32-60[5]	133[a]	± 8	4.3 ± 0.2	8.8 ± 0.5
Seagrass	248-366	33	± 9	8 ± 2	12 ± 3
Total vegetated habitats		305	± 22	23 ± 3	41 ± 4
**% Seagrass**				**35 ± 10**	**29 ± 8**
Depositional marine areas					
Continental margin				248[8]	
Total coastal OC burial				271 ± 3	289 ± 4
Deep Sea burial				20 ± 6[9]	
Total oceanic OC burial				291 ± 7	309 ± 7
**% Seagrass** (based on mapped area)				**2.7 ± 0.7**	**3.9 ± 1.0**

[a] Median OC burial rate from Wang et al. (2021) was used as the most appropriate measure of central tendency.

Supplementary references

1. Dürr, H. H. *et al.* Worldwide Typology of Nearshore Coastal Systems: Defining the Estuarine Filter of River Inputs to the Oceans. *Estuaries and Coasts* **34**, 441–458 (2011).
2. Duarte, C. M., Middelburg, J. J. & Caraco, N. Major role of marine vegetation on the oceanic carbon cycle. *Biogeosciences* **2**, 1–8 (2005).
3. Hamilton, S. E. & Casey, D. Creation of a high spatio-temporal resolution global database of continuous mangrove forest cover for the 21st century (CGMFC-21). *Global Ecology and Biogeography* **25**, 729–738 (2016).
4. Worthington, T. A. *et al.* A global biophysical typology of mangroves and its relevance for ecosystem structure and deforestation. *Sci. Rep.* **10**, 1–11 (2020).
5. Worthington, T. A. *et al.* The distribution of global tidal marshes from Earth observation data. *Global Ecology and Biogeography* **33**, 1–13 (2024).
6. Wang, F. *et al.* Global blue carbon accumulation in tidal wetlands increases with climate change. *Natl. Sci. Rev.* **8**, (2021).
7. Breithaupt, J. L. & Steinmuller, H. E. Refining the Global Estimate of Mangrove Carbon Burial Rates Using Sedimentary and Geomorphic Settings. *Geophys. Res. Lett.* **49**, (2022).
8. Burdige, D. J. Preservation of organic matter in marine sediments: Controls, mechanisms, and an imbalance in sediment organic carbon budgets? *Chem. Rev.* **107**, 467–485 (2007).
9. Hayes, C. T. *et al.* Global Ocean Sediment Composition and Burial Flux in the Deep Sea. *Global Biogeochem. Cycles* **35**, 1–25 (2021).
