# Supplementary material for: Seagrass sediment organic carbon burial rates are globally significant
Source: Nat Commun. 2026 Nov 4;17:11494. doi: 10.1038/s41467-026-79168-3 (PMC13743778; doi:10.1038/s41467-026-79168-3)
Supplement: Supplementary file 2 [file 41467_2026_79168_MOESM2_ESM.pdf]

Description Of Additional Supplementary Files

Supplementary dataset 1: Dataset containing reported seagrass area by country
