# Supplementary material for: Seagrass sediment organic carbon burial rates are globally significant
Source: Nat Commun. 2026 Nov 4;17:11494. doi: 10.1038/s41467-026-79168-3 (PMC13743778; doi:10.1038/s41467-026-79168-3)
Supplement: Supplementary file 3 [file 41467_2026_79168_MOESM4_ESM.pdf]

nature portfolio

Peer Review File

Seagrass sediment organic carbon burial rates are globally significant

Corresponding Author: Dr Ariane Arias-Ortiz

This file contains all reviewer reports in order by version, followed by all author rebuttals in order by version.

Version 0:

Reviewer comments:

Reviewer #1

(Remarks to the Author)
This manuscript presents a global meta-analysis of carbon sequestration rates across blue carbon ecosystems and proposes a revised estimate of global carbon burial capacity. While the compilation effort is substantial and the topic is of high relevance, I have several major concerns regarding the conceptual framing, methodological robustness, and interpretation of the results, particularly in relation to previous global syntheses produced by largely overlapping author groups. A central issue is that the manuscript repeatedly redefines global estimates without sufficiently clarifying whether these revisions represent methodological refinement, contextual re-interpretation, or genuine correction of earlier values. Given the strong influence of previous global blue carbon assessments—many authored by the same research group—greater transparency and caution are required when "updating" global numbers that have already been widely adopted in scientific literature and policy frameworks.
1. Conceptual novelty and scientific necessity
The manuscript claims to substantially revise global carbon sequestration rates compared to previous estimates. However, many of these earlier estimates were derived from datasets compiled or synthesized by overlapping author teams. The authors should more explicitly address: Whether the differences arise from new empirical data, revised inclusion/exclusion criteria, or alternative statistical treatments.Why earlier estimates—widely cited and used in policy contexts—should now be considered inadequate rather than context-dependent.Without such clarification, the repeated re-estimation of global values risks appearing incremental rather than conceptually transformative.
2. Data representativeness and regional bias
Although presented as a "global" synthesis, the underlying dataset remains heavily skewed toward a limited number of regions and ecosystems. Large portions of the tropics, Global South coastlines, and island-dominated systems remain underrepresented.The authors should:Provide a quantitative assessment of spatial bias (e.g., percentage of data from each biogeographic region).Explicitly discuss how such imbalance affects the reliability of global extrapolations.At present, the manuscript may overstate global applicability while relying on regionally clustered data.
3.Methodological circularity and consistency
Given that several previous global blue carbon syntheses by the same authors are now being revised, it is essential to clarify whether the current approach corrects methodological limitations of earlier work or simply applies alternative assumptions. Specifically:Were previous studies biased, incomplete, or methodologically inconsistent? If so, why were these limitations not acknowledged at the time? If not, why should the current estimates supersede earlier values? This issue is particularly important given the extensive citation and downstream use of prior estimates.
4.Uncertainty quantification and communication
While the manuscript reports confidence intervals, the broader structural uncertainties associated with ecosystem heterogeneity, methodological diversity, and data scarcity are not fully addressed.I strongly recommend a more explicit discussion distinguishing statistical uncertainty from epistemic uncertainty, avoiding definitive language (e.g., "true global rate") in favor of scenario- or assumption-dependent estimates.Overconfidence in global numbers may mislead both researchers and policymakers.
5.Policy relevance and responsible interpretation
The manuscript draws direct implications for global climate mitigation strategies and carbon accounting frameworks. However, given the substantial variability and uncertainty in the underlying data, such policy-oriented conclusions should be framed more cautiously. In particular: The authors should avoid implying that revised global estimates directly translate into improved policy accuracy. Regional management and governance implications may be more appropriate than global generalizations.

Reviewer #2

(Remarks to the Author)
Review of NCOMMS-25-103039: ‘Seagrass sediment organic carbon burial rates are globally significant,’ by Arias-Ortiz et al.

Summary

This paper presents a revised global estimate for organic carbon burial in seagrass meadows, based on 222 previously-published sediment cores from around the world. The authors summarize sediment accumulation rates, percent organic carbon and organic carbon burial rates by region and include both vegetated and unvegetated sediment. They use these data to determine important geochemical relationships on a global scale, including the relationship between % organic carbon and sediment accumulation rate. They conclude that the previous global estimate was too high and revise it downwards, by a factor of four, to 6-16 Tg C/yr. Furthermore, they have compiled the data into an open source data set.

Major comments

This paper represents a significant advance from earlier global seagrass carbon burial estimates, which were based on sediment cores from one region. The authors have directly addressed some of the other problems common to previous papers as well. In particular, they have addressed the difference between carbon stock and carbon burial, and determined burial rates for different genera of seagrass. The paper is very well-presented, with clear and useful figures, and the open-source data set will be valuable to other researchers.

However, two major problems from earlier estimates still remain.

1) The most significant is that their carbon burial rates include allochthonous carbon. Williamson et al. (2025. Global Change Biology 31(4):e70181) showed, based on a global data compilation, that the median contribution of allochthonous carbon to total carbon burial in seagrass meadows is 74%. About three-quarters of the carbon buried in seagrass meadows comes from elsewhere, mainly from land, and would likely have been buried in coastal sediments, regardless of the presence or absence of the seagrass meadow. Allochthonous material cannot be attributed to the seagrass meadow. Since the proportion of allochthonous carbon is not always reported, the authors probably do not have access to this information for their whole data set. In such cases, Williamson et al suggested that a default value be used, based on the global median.

I recommend that the authors reduce their global seagrass carbon burial rate by 74% to account for this.

2) The authors do not appear to have accounted for remineralization of organic carbon in surface sediment. (If they have, then they need to clarify this point.) They used previously-published organic carbon burial rates or recalculated the rates based on existing data. They calculated the burial rates as the product of the mass accumulation rate and the fraction of organic carbon, which is correct. However it appears that they used the fraction of organic carbon all the way from the surface to the bottom depth of excess Pb-210 (Line 545). The % organic carbon at the surface is higher than the % buried, because benthic animals and bacteria consume organic carbon in surface sediments, often down to a depth of 20 cm or more. A carbon burial rate calculated using the surface % organic carbon is too high. This error is not as large as that from the inclusion of the allochthonous material, but it does artificially inflate the carbon burial rate. This point is explained in detail in a recent methods paper (Johannessen, 2023. Carbon Footprints 2(21): DOI: 10.20517/cf.2023.37) The methods paper does not necessarily need to be cited in the revised manuscript; I only include it here for the authors’ information.

I suggest that the authors recalculate their carbon burial rates, using only the % organic carbon from the deepest sections of the cores, and excluding the surface. With such a well-organized data synthesis, it should not be too difficult to recalculate the rates. An alternative would be to calculate the ratio of the % organic C at 20 cm or greater to that at the surface for as many cores as possible and then reduce their global rate by that factor.

I recommend that the paper be accepted with major revisions.

Detailed comments

Line 55. Change “are” to “have been,” since this paper overturns the previous value.

Lines 115-116. Add a reference for the Tier 1 default value.

Figure 1. This is a useful figure. It illustrates the broad coverage of this data set, and the inset provides useful context about the latitudinal distribution of samples.

Lines 139-141. Although these values were published, given the widespread errors in carbon burial calculation, some (or many) of them might be incorrect, mainly too high. Did the authors assess the validity of the published rates where possible?

Figure 2. This is a useful figure that presents a lot of information clearly.

Figure 3a. Enlarge the main panel of this figure, so that the legend will fit inside. I missed the legend at first.

Lines 277-278 and Figure 4. First mention of seagrass-sediment isotopic offset. How was this calculated? What is the range of dC-13 values for seagrass? For sediment? Explain why this parameter was used, what it means and why it might vary with water depth. How do these values compare with those of particulate matter from nearby terrestrial runoff or phytoplankton? Why does there seem to be a larger seagrass-sediment difference in areas of low carbon accumulation? Include a reference about the use of stable isotopes to identify the origin of organic matter in a seagrass meadow.

Lines 339-345. How is “unvegetated sediment” in a seagrass meadow defined? How close did it have to be to living seagrass, or how large was the open patch? If these unvegetated patches were very small and located in the middle of a seagrass meadow, it is not surprising that they had similar organic carbon burial rates. A seagrass frond falling over sideways on a scale of tens of cm wouldn’t really qualify as being “exported” from the meadow. Clarify what these samples represent.

Line 355, line 358. The sentence includes both “although colonized by seagrasses” and “irrespective of seagrass cover.” Clarify. It would probably help to break this into two sentences.

Lines 359-361. Explain how the isotopic offset indicates the contribution of autochthonous and allochthonous sources.

Lines 362-363. The similarity among isotopic offsets in vegetated, non-vegetated, depositional and non-depositional environments might alternatively mean that seagrass carbon is not a significant contributor to the sediment in any of these settings… Did the authors determine the proportional contribution of seagrass carbon in the various environments?

Figure 5. How was the seagrass-sediment isotopic parameter determined for unvegetated sediment, where there was presumably not any seagrass to measure?

Figure caption. Spell out DBD on first use (line 391).

Lines 411-415. This sentence would be clearer with brackets around “which support extensive meadows.”

Line 417. This global rate appears to include allochthonous carbon. See major comment #1.

Line 446. Change “its” to “their” or make “seagrasses” singular.

Line 476. Define Tier 2 carbon accounting and provide a reference.

Figure 6. The dashed lines for “Tier 1” and “updated” OC burial rates are too similar. Make one dotted or solid to contrast with the dashed line.

Lines 505-507. 16% of vegetated sediments were non-depositional (compared with 30% of unvegetated sediment, Figure 5), so the presence of seagrass is not a reliable indicator of carbon burial.

Line 545. What does “the fraction of OC accumulated down to the excess 210Pb horizon” mean? Were the organic carbon values averaged from the surface to the bottom? Was only the deep value used? Clarify this point. If surface or shallow (< 15 cm) values were used, then the resulting C-burial rates are too high, because of the remineralization of organic carbon in surface sediment.

Line 558-559. High mixing rates only bias Pb-210-derived sedimentation rates high if Pb-210 data within the surface mixed layer are included. Those cores can give correct rates if researchers exclude the surface mixed layer and determine the sediment accumulation rate based on the rate of decay of Pb-210 below that. See Johannessen (2023) paper cited in major comment #2.

Sophia Johannessen
Research Scientist, Fisheries and Oceans Canada
February 6, 2026

Reviewer #3

(Remarks to the Author)
This manuscript represents a significant update to global organic carbon burial rates in seagrass habitats, constraining earlier estimates that relied heavily on production-based approaches which may not accurately reflect actual burial rates. It also improves previous estimates by updating global surface areas of seagrass habitats, a key but somewhat overlooked factor. The use of a global sediment core database to validate existing estimates is a substantial step forward in carbon accounting within the global coastal carbon budget. The comparison between rates of sediment accumulation and organic carbon burial, and organic carbon content provide insightful analysis of the temporal, hydrodynamic and sedimentary characteristics of different global regions on seagrass carbon dynamics. I particularly appreciate the acknowledgement of estuarine geomorphological influence on organic carbon retention by seagrass habitats and agree that more analysis is

warranted, albeit outside the current study scope. I found that the results were in-line with the researchers findings.

While the figures are dense and informative, they could benefit from finetuning the colors to enhance clarity. For example, the datapoints and plots in figures 4C, D, and E, and could benefit from greater contrast using different colors to improve distinction. The characteristics of presented data in Figure 2 could also be clearer if an x-axis break was added to spread out data around the Q1 and Q3 bounds to improve clarity; possibly also for figure 6. Figure 1 could also benefit from clearer marker shapes as they currently can appear as indistinct clusters of points in concentrated areas.

Comparisons to reported results in the literature could be clearer by explicitly quantifying the extent of differences mentioned throughout the discussion, for example in lines 104, 373, and 454. Additionally, regarding the complete dataset mentioned in line 552, a cross-reference would facilitate accessibility. The method section gave a good overview of the study's approach and i did not find any major flaws. However, a summary table documenting the different methods used on different cores might enhance clarity compared to just a text-only description. However, i acknowledge that this is a personal preference given that the current description is adequate. Lines 572-576 reference equations but they were not presented explicitly. While in-line references were provided to the referenced studies, including the equations directly would strengthen the manuscript as a standalone study.

Overall, this manuscript's findings are noteworthy for publication and represent a significant advancement in our understanding of the role of seagrass habitats in organic carbon burial as part of the global coastal organic carbon budget.

Version 1:

Reviewer comments:

Reviewer #1

(Remarks to the Author)
I would like to thank the authors for their efforts in revising the manuscript and addressing my previous concerns. The manuscript has certainly improved and the scientific narrative is much clearer. However, before I can fully recommend this manuscript for publication in Nature Communications, there are several critical issues that still need to be addressed.
general comments
1. Recently published in Nature paper of 'Global high-resolution mapping of seagrass to support conservation' should be added in this MS, because authors should consider whether your work need to update the global seagrass area.
2. The authors rely exclusively on ^{210}Pb geochronology, which integrates OC burial over the past ~100 years. Ecologically, this specific century coincides with the most severe, human-induced degradation of coastal ecosystems globally (eutrophication, coastal development, dredging).The authors should explicitly discuss the "shifting baseline syndrome." Is the geometric mean of 25-33 g OC m^{-2} yr^{-1} representative of a "pristine" global seagrass sink, or does it reflect the burial capacity of already stressed, fragmented, or recovering meadows during the Anthropocene? Acknowledging that modern burial rates might be compromised by recent ecological degradation would add profound depth to the climate mitigation discussion.
3. The finding that adjacent bare sediments exhibit comparable OC burial rates and 13C offsets to vegetated sediments (Fig. 5) is ecologically fascinating. It highlights the "spillover" or "halo effect" of seagrass canopies. However, "unvegetated" is a relative term that requires tighter spatial definition. Please clarify the spatial scale of these adjacent bare cores (e.g., were they within the meadow patch matrix (gaps), 10 meters away, or 1 kilometer away?). From a hydrodynamic perspective, the seagrass canopy creates a 3D hydrodynamic shadow that extends beyond the meadow edge, facilitating fine particle deposition in nearby bare patches. Explicitly discussing this benthic-pelagic spatial coupling will strengthen the argument that seagrasses drive landscape-scale OC burial, even if the carbon ultimately settles outside the meadow footprint.
4. From a geochronological standpoint, excluding or flagging cores with pervasive downcore mixing (Quality Flag 3, representing ~26% of cores) is completely justified to ensure accurate MAR calculations. However, from a benthic ecology perspective, this intense mixing (bioturbation by callianassid shrimp, polychaetes, etc.) is a fundamental driver of OC remineralization, as it introduces oxygen into deep sediments.The authors should briefly discuss the ecological implications of this exclusion. By mathematically sidestepping highly bioturbated sites, is there a risk of slightly overestimating or underestimating the global net preservation? Acknowledging bioturbation as a major biological filter for OC burial (not just a methodological hurdle) would integrate benthic ecology nicely with the geochemical narrative.
5. In the discussion (Lines 376-401), the authors attribute high OC stocks to persistent species (Posidonia, Thalassia), which is accurate. However, the explanation relies heavily on their sedimentary environment (MAR). I suggest adding a brief mechanistic explanation based on plant functional traits. Persistent species not only occupy specific geomorphic settings but also invest heavily in belowground biomass (extensive root/rhizome networks) and possess highly recalcitrant structural tissues (high lignin/cellulose, high C:N/C:P ratios) compared to colonizing species like Halophila. Emphasizing that the intrinsic chemical recalcitrance of the plant tissue actively resists microbial degradation will strengthen the biological rationale behind the observed taxonomic patterns.

minor comments
1. In the Abstract, simply reporting the global OC burial rate of seagrass meadows is not sufficient and lacks novelty. The manuscript also discusses the relationship between sediment OC content and OC burial rates, and this should be highlighted in the Abstract.
2. In Figure 2, adding the percentage values representing the proportional seagrass area of each bioregion to the pie chart.
3. Please provide the sample sizes for the boxplots in Figures and 5.
4. According to the manuscript, global seagrass extent is reported as a range (247,800–366,400 km^2). It is therefore unclear

why the relative area contribution of each seagrass bioregion was treated as a fixed value. Please provide a clearer explanation in the Methods section regarding how the bioregional area proportions were derived.
5. For Figure 4, although the authors describe the criteria used to group OC burial rates in the Statistical analysis section, it is suggested that the actual numerical range corresponding to each group be provided, which would make the figure more intuitive and easier to understand. In addition, MAR also appears to be grouped in this figure, and the criteria for grouping MAR should also be clearly described in the Statistical analysis section.
6. The sediment core dataset is stated to be available through the Smithsonian's Figshare data repository: https://doi.org/10.25573/serc.32592150. However, this database link cannot be accessed.

Reviewer #2

(Remarks to the Author)
I appreciate the authors' thorough, thoughtful and creative responses to my comments. I recommend that the revised paper be accepted.

Version 2:

Reviewer comments:

Reviewer #1

(Remarks to the Author)
I have no more comments.

Open Access This Peer Review File is licensed under a Creative Commons Attribution 4.0 International License, which permits use, sharing, adaptation, distribution and reproduction in any medium or format, as long as you give appropriate credit to the original author(s) and the source, provide a link to the Creative Commons license, and indicate if changes were made.
In cases where reviewers are anonymous, credit should be given to 'Anonymous Referee' and the source.
The images or other third party material in this Peer Review File are included in the article's Creative Commons license, unless indicated otherwise in a credit line to the material. If material is not included in the article's Creative Commons license and your intended use is not permitted by statutory regulation or exceeds the permitted use, you will need to obtain permission directly from the copyright holder.
To view a copy of this license, visit https://creativecommons.org/licenses/by/4.0/

Editor

As you will see from the reports copied below, the reviewers raise important concerns. We find that these concerns limit the strength of the study, and therefore we ask you to address them with additional work. Without substantial revisions, we will be unlikely to send the paper back to review. In particular, it is essential to verify and strengthen the robustness of the methodology and reliability of results. The advancement beyond the previous publications should be supported by clearer articulation of the conceptual framework, improved assessment of methodological robustness (particularly regarding the data bias), and more careful interpretation of the results. In addition, the carbon burial rates are suggested to be recalculated by re-evaluating the contributions of allochthonous carbon and the remineralization of organic carbon in surface sediments. Finally, improving the clarity of the figures and the overall narrative will be important for better conveying the study's scientific contributions.

If you feel that you are able to comprehensively address the reviewers' concerns, please provide a point-by-point response to these comments along with your revision. Please show all changes in the manuscript text file with track changes or colour highlighting. If you are unable to address specific reviewer requests or find any points invalid, please explain why in the point-by-point response.

Thanks for the opportunity to respond to the reviewers' comments and address the issues raised in the revised manuscript. Our point-by-point responses are provided below in blue.

All data have been made available via Figshare through the Coastal Carbon Network and will be accessible through the Coastal Carbon Atlas, thereby increasing the visibility of both the dataset and the manuscript. Along with the dataset containing all depth-series data we have also included a summary table with the summarized carbon accumulation rates with ancillary data necessary to replicate the figures. In addition to addressing the reviewers' comments, we have also taken the opportunity to include one additional study in the synthesis dataset.

A private link to the dataset is here: https://figshare.com/s/bda2144e873b0bf9983c

Reviewer #1

This manuscript presents a global meta-analysis of carbon sequestration rates across blue carbon ecosystems and proposes a revised estimate of global carbon burial capacity. While the compilation effort is substantial and the topic is of high relevance, I have several major concerns regarding the conceptual framing, methodological robustness, and interpretation of the results, particularly in relation to previous global syntheses produced by largely overlapping author groups. A central issue is that the manuscript repeatedly

redefines global estimates without sufficiently clarifying whether these revisions represent methodological refinement, contextual re-interpretation, or genuine correction of earlier values. Given the strong influence of previous global blue carbon assessments—many authored by the same research group—greater transparency and caution are required when “updating” global numbers that have already been widely adopted in scientific literature and policy frameworks.

We thank the reviewer for this comment. Several global syntheses of seagrass sediment OC stocks have been published (e.g., Fourqurean et al., 2012; Krause et al., 2025) and indeed include overlapping author teams. However, this manuscript presents a synthesis of OC burial rates, not OC stocks. In fact, global syntheses of OC burial rates are much more limited and often combine estimates derived using different methodologies and over different timescales. The most widely cited global synthesis of seagrass OC burial rates is that of McLeod et al. (2011), with only one author overlapping with the present study.

Our synthesis is based on a substantially expanded and more transparent dataset compared to previous global assessments, which relied on more limited and in some cases methodologically heterogeneous data sources. For example, IPCC Tier 1 global default factor (2014) was based on six carbon burial rates from a single species (*Posidonia oceanica*), resulting in spatial and taxonomic bias. McLeod et al. (2011) combined a small number of direct burial measurements from *Posidonia oceanica* (n = 7) with net community production estimates, sediment trap data, biomass accumulation, and carbon mass balances, thereby mixing approaches that integrate different temporal scales (short- and long-term) and methodological assumptions.

We therefore consider that these earlier syntheses are subject to important limitations that justify the updated estimates reported in this manuscript, which are based on a more consistent methodology (Pb-210 radiometric dating of sediments over the last century) and an expanded dataset (220 direct organic carbon burial rate estimates) that cover multiple seagrass species and a broader geographical area. In contrast, previous syntheses focused primarily on Posidonia oceanica meadows in the Mediterranean Sea or included net community production estimates derived largely from the Mediterranean Sea and the North Atlantic. All data used in this synthesis is made publicly available and accessible.

We have revised the manuscript to clarify how our estimates relate to previous global assessments. Please, see the response to the comment below for more details.

References

Fourqurean, J. W. et al. Seagrass ecosystems as a globally significant carbon stock. Nat. Geosci. 5, 505–509 (2012).

Krause, J. R. et al. Global seagrass carbon stock variability and emissions from seagrass loss. Nature Communications 16, 1–9 (2025).

1. Conceptual novelty and scientific necessity

The manuscript claims to substantially revise global carbon sequestration rates compared to previous estimates. However, many of these earlier estimates were derived from datasets compiled or synthesized by overlapping author teams. The authors should more explicitly address: Whether the differences arise from new empirical data, revised inclusion/exclusion criteria, or alternative statistical treatments. Why earlier estimates—widely cited and used in policy contexts—should now be considered inadequate rather than context-dependent. Without such clarification, the repeated re-estimation of global values risks appearing incremental rather than conceptually transformative.

While syntheses of seagrass sediment OC stocks have been published (e.g., Fourqurean et al., 2012; Krause et al., 2025) with some overlap in the author team, there aren't recent synthesis of seagrass OC burial rates. The most widely cited synthesis of seagrass OC burial rates (McLeod et al., 2011) shares only one author with the present study, was conducted more than a decade ago, and was based on a limited dataset based on indirect burial rate estimates. Our study uses an independent compilation containing approximately 50 times more direct OC burial rate estimates, substantially expands geographic coverage, and harmonizes all observations to common methods and timescales. As a result, the updated global estimate is fourfold lower than that reported by McLeod et al. (2011), representing a substantial reassessment rather than an incremental update of previous estimates.

Earlier syntheses on OC burial rates were constrained by the available data at the time they were produced, which introduced limitations including species and spatial biases, as well as methodological heterogeneity. We are not aiming to criticize the original work but rather reflect on the state of the science at that time.

Global OC burial rates being cited and used in policy contexts are biased toward the matte-forming *P. oceanica* (IPCC, 2014) or combine direct and indirect OC burial estimates from a mixture of methodological approaches representing processes operating over different temporal scales (carbon mass balances, long-term (millennial) radiocarbon OC burial, sediment traps, or net ecosystem uptake) (McLeod et al., 2011). The OC burial rate

estimates in this study are derived by applying a single methodology across a common temporal scale (rates derived from Pb-210 dating over the last decades/century) and multiple seagrass bioregions, and by including 50 times more direct estimates of sediment OC burial rates than previous global syntheses. Because earlier assessments were based on a heterogeneous mix of methods and timescales and predated the widespread application of ^{210}Pb-derived burial rates in seagrass ecosystems, there is no overlap between their datasets and ours. Consequently, this synthesis is independent from previous assessments while being substantially larger and methodologically more consistent. It includes OC burial rate estimates compiled from both our own published studies and those of many other research groups worldwide.

We have revised the manuscript to clarify the limitations of previous estimates and to explain how our approach differs from them.

In the Main introduction:

L70-81: "*Previous global estimates[2,10] were limited by the scarcity of direct burial rate measurements and the use of heterogeneous methodological approaches that quantified different components of OC burial. The most commonly used global value of 138 ± 38 g OC m^{-2} yr^{-1}[2] was derived from a small number of direct radiocarbon-dated Posidonia oceanica mattes (n = 7) [1,12] and a range of indirect approaches, including mass balance[12], biomass accretion[13], or sediment traps[14] along with seagrass net community production (NCP)[15] and burial of allochthonous OC[16], integrating processes over timescales spanning days to millennia. To reduce methodological heterogeneity for national greenhouse gas reporting, the IPCC adopted a Tier 1 default value (43 g OC m^{-2} yr^{-1}) [10] based on six directly measured radiocarbon-based P. oceanica burial rates, excluding indirect or process-inferred estimates, thereby ensuring internal consistency but at the expense of strong taxonomic and regional bias.*"

Results section "*Organic carbon burial rates in seagrass sediments*":

L215-228: *"The differences in sediment OC burial rates relative to previous syntheses stem from the inclusion of ~50 times more direct estimates than previous global assessments, a broader geographic coverage, and the systematic use of ^{210}Pb geochronology, which integrates OC burial over a consistent decadal-centennial timescale. The largest differences were observed relative to McLeod et al.[2], which were based on limited direct measurements and relied on indirect approaches representing short-term sequestration processes. Such methods can often result in higher values due to their focus on rapidly cycling carbon pools with a greater contribution of labile organic matter. In contrast, our*

analysis captures the mean in situ accumulation of OC in seagrass sediments over the last century, excluding export and burial beyond the meadows, sequestration in biomass, and short-term net ecosystem uptake. While our estimates include surface sediment OC that may not have undergone complete decomposition, this contribution was small, although variable. Over a centennial timescale, 51% of vegetated sediment profiles had stable OC concentrations with depth, 39% decreased, and 10% increased, yielding a median bottom-to-surface OC concentration ratio of 0.70."

2. Data representativeness and regional bias

Although presented as a "global" synthesis, the underlying dataset remains heavily skewed toward a limited number of regions and ecosystems. Large portions of the tropics, Global South coastlines, and island-dominated systems remain underrepresented.The authors should:Provide a quantitative assessment of spatial bias (e.g., percentage of data from each biogeographic region).Explicitly discuss how such imbalance affects the reliability of global extrapolations. At present, the manuscript may overstate global applicability while relying on regionally clustered data.

We acknowledge that some regions remain underrepresented due to data scarcity and that spatial imbalance introduces uncertainty, particularly in regional values. However, relative to previous syntheses, this study incorporates a broader biogeographic coverage and a much larger number of direct observations (220 vs. 6). Previous syntheses were either strongly biased toward *P. oceanica* meadows in the Mediterranean region or based on a smaller number of observations (e.g., McLeod et al. 2011, n = 123 combining direct and indirect approaches). Our synthesis includes 332 sediment cores, of which 220 provide quantifiable direct estimates of organic carbon burial rates.

Importantly, the consistency of mean and median estimates, as well as the reduced uncertainty with increasing sample size (Fig. 3a and new Fig S1 and S3), suggests diminishing sensitivity of the reported estimate to additional observations, indicating the current dataset captures the central tendency of global seagrass OC burial reasonably well. However, while the pooled dataset means may have reached a steady-state value, we acknowledge this is not necessarily true for all the bioregion means, particularly bioregions represented by few data points such as the Tropical-Indo Pacific (n = 12).

We have revised the manuscript text to provide a quantitative assessment of spatial bias and at the inset of Fig. 1, we show a bar chart of the core % by 5º latitudinal band:

L133-139: *"data were geographically concentrated in Australia, North America, and the Mediterranean Sea, while data were sparse for South America, Africa, South and Southeast Asia, despite the extensive seagrass distribution in the latter. By bioregion, 16% of the*

estimates originated from the Mediterranean, while 20 and 26% came from the Tropical Atlantic and Southern Oceans (primarily from the tropical Western Atlantic, and Australia, respectively), with the remaining regions contributing ~12-13% each."

Additionally, we have added text to highlight uncertainties associated to the new estimates and discuss data imbalances along a new supplementary figure (S1) with stabilization curves for each bioregion, to highlight that for the Tropical-Indo Pacific, saturation of the OC burial rate has not yet been achieved and there is the even more need for more studies in that region.

L159-167: *"Considering all depositional seagrass sediments, the global geometric mean OC burial rate was 25 ± 3 g OC m^{-2} yr^{-1} (95% CI: 22–29; n = 207), a value that remained similar even when excluding records affected by intense sediment mixing (27 ± 2 g OC m^{-2} yr^{-1}; 95% CI: 23–31; n = 127). Remarkably, this pooled, global estimate has stabilized with the rapid addition of new data since 2018, resulting in low sensitivity to additional measurements and reduced standard errors (Fig. 3a, Fig. S1). However, it remains influenced by the uneven geographical distribution of observations, with OC burial rates from Australia, the USA, and Spain disproportionally contributing to the dataset (Fig. S2)."*

L173-182: *"Considering the different seagrass extent across bioregions, we calculated an area-weighted average OC burial rate of 33 ± 9 g OC m^{-2} yr^{-1} (95% CI: 25–41), which was comparable to the geometric mean estimate (Fig. 2a). While this area-weighted estimate is less influenced by sampling density, it is driven by bioregions with the largest seagrass coverage. Consequently, it remains sensitive to uncertainties in mapped seagrass area and to the limited data available from the Tropical Indo-Pacific, where regional means are still dependent on sample size (Fig. S1). Nevertheless, the two values are statistically similar, despite the different sources of uncertainty, and provide robust and complementary estimates of global OC burial rates in seagrass sediments."*

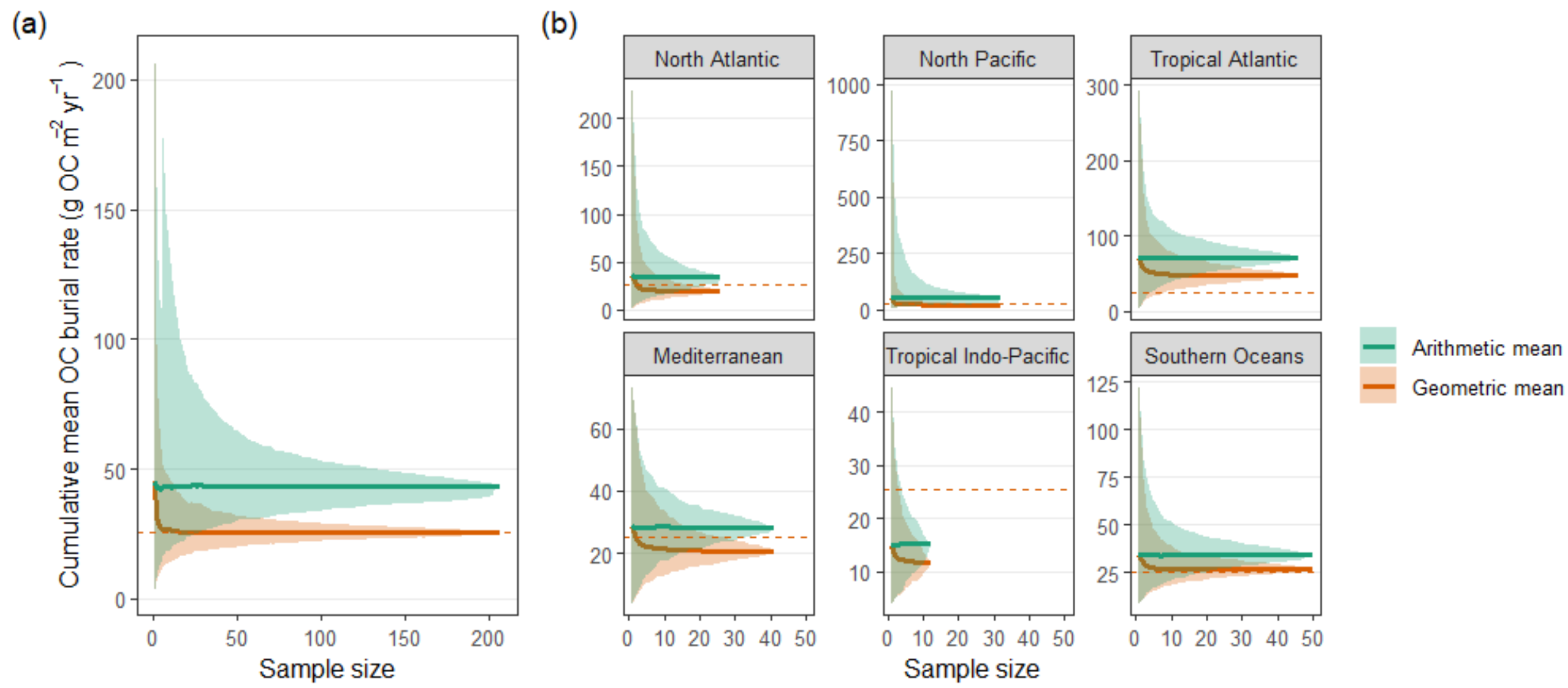

New: **Figure S1**. Stability of estimated organic carbon burial rates under increasing sample size. (a) Cumulative arithmetic and geometric means of OC burial rate as a function of sample size, based on 1,000 random permutations of all vegetated cores with net accumulation combined; (b) regional subsets. Shaded areas represent 95% confidence intervals. The dashed line indicates the geometric mean OC burial rate across the dataset (25 g OC m^{-2} yr^{-1}).

3.Methodological circularity and consistency

Given that several previous global blue carbon syntheses by the same authors are now being revised, it is essential to clarify whether the current approach corrects methodological limitations of earlier work or simply applies alternative assumptions. Specifically:Were previous studies biased, incomplete, or methodologically inconsistent? If so, why were these limitations not acknowledged at the time? If not, why should the current estimates supersede earlier values? This issue is particularly important given the extensive citation and downstream use of prior estimates.

As noted in Comment #1, previous syntheses of OC burial rates were limited in number and relied on datasets that were either taxonomically biased or based on indirect proxies that do not necessarily represent OC burial (e.g., net community production). These syntheses are now over a decade old, while our estimate compiles all available data derived using ^{210}Pb generated since the rise of blue carbon research. Our estimate is approximately fourfold lower, is based on consistent methods and timescales, and yields stable mean and geometric mean values as new data are added. Beyond providing a global value, the public nature of the dataset allows disaggregation by species, bioregions, taxa, and geomorphological settings, enabling region-, species-, and sediment-specific estimates, should these be of interest.

Differences between previous global OC burial rates and the current estimate are explained in detail in Comment #1, and data compilation, and statistical treatment are described in the Methods section (L558-614 and L706-725).

4.Uncertainty quantification and communication
While the manuscript reports confidence intervals, the broader structural uncertainties associated with ecosystem heterogeneity, methodological diversity, and data scarcity are not fully addressed.I strongly recommend a more explicit discussion distinguishing statistical uncertainty from epistemic uncertainty, avoiding definitive language (e.g., "true global rate") in favor of scenario- or assumption-dependent estimates.Overconfidence in global numbers may mislead both researchers and policymakers.

We have revised the manuscript and made sure there were no instances of definitive language like "true global rate". We have also replaced the term "global" with "across the dataset" or similar phrasing where it was appropriate.

Additionally, we have highlighted the uncertainties associated with the main areal OC burial rate estimates derived in this manuscript. In the revised version, we clarify that the pooled geometric mean is influenced by regions with high sampling density, particularly Australia, the USA, and Spain (L159–167; see response to Comment #2). In contrast, the area-weighted average is sensitive to uncertainties in mapped seagrass extent and to the limited availability of data from the Tropical Indo-Pacific, where seagrass cover is extensive, but burial rate estimates remain scarce (L173–182; see response to Comment #2).

Finally, we have substantially rewritten the last section of the manuscript to clarify how the different OC burial rate estimates should be interpreted according to their intended applications (L485–533).

We have not reproduced the full revised section titled "*Constraints on interpreting OC burial rates*" here because of its length. Instead, we include the first paragraphs, which is most relevant to the reviewer's comment.

L485-512*: "Challenges and pitfalls of utilizing OC burial rate estimates in blue carbon ecosystems for climate change mitigation have been described elsewhere[23,65], being long-term permanence and the attribution of OC source, particularly the treatment of allochthonous inputs, dominant topics of debate [66–68]. These discussions highlight the necessity of clarifying the interpretation and appropriate application of the global estimates presented here. The rates reported in this study represent data-constrained estimates of total OC burial in seagrass sediments integrated over a centennial timescale. They do not provide a direct measure of climate mitigation; rather, they are reference values describing contemporary OC burial within seagrass meadows. As such, they can serve as baseline*

conditions prior to habitat degradation, or as target conditions following restoration when site-specific measurements are unavailable. Consequently, these estimates are best suited for global carbon-cycle studies and IPCC Tier 1 default factors, rather than for representing processes at local scales, where the large spatial variability in OC burial rates remains insufficiently constrained (Fig. S7). Patterns across bioregions and geomorphic settings remain poorly resolved even within well sampled regions (Fig. 2), with site-level variability, expressed as the coefficient of variation, averaging ~50% of the mean (Fig. S7). This pronounced heterogeneity suggests the need for denser, stratified sampling designs that capture depth and regional gradients within countries to improve the accuracy of regional and national estimates.

If OC burial rates are used in the context of climate mitigation, the primary consideration is the timescale of interest. Within the contemporary carbon cycle, the climate-relevant metric is the net change in burial fluxes resulting from ecosystem degradation, conservation, or restoration, rather than the baseline absolute OC burial rates reported here."

5.Policy relevance and responsible interpretation

The manuscript draws direct implications for global climate mitigation strategies and carbon accounting frameworks. However, given the substantial variability and uncertainty in the underlying data, such policy-oriented conclusions should be framed more cautiously. In particular: The authors should avoid implying that revised global estimates directly translate into improved policy accuracy. Regional management and governance implications may be more appropriate than global generalizations.

We agree with the need for more cautious framing of policy implications. In response, we have revised the manuscript to clarify the appropriate scope and application of the OC burial rate estimates. We have removed the previous Figure 6, which presented country-specific OC burial rates, as our results show that Tier 2 applications remain premature given current data limitations.

Current IPCC Tier 1 values are already widely used in policy and carbon accounting frameworks with its known biases and uncertainties. In this context, our synthesis provides an improved estimate that incorporates a larger and more geographically and taxonomically diverse dataset than that underlying the currently adopted Tier 1 value (see response to Comment #1). Briefly, the previous estimate was based on only six radiocarbon burial rate measurements from *Posidonia oceanica*. By incorporating rates from a broader range of species, regions, and environmental settings, our synthesis could increase the representativeness of the Tier 1 default value if revised.

Nevertheless, local and regional estimates remain more appropriate than global Tier 1 values whenever sufficient site-specific data are available. We have revised the manuscript to avoid over-interpretation at policy-relevant scales. Please see the new manuscript section “Constraining the interpretation of OC burial rates” (**L485–533**), which aims to guide the interpretation of reported OC burial rates depending on context and intended application.

Reviewer #2 (Remarks to the Author):

This paper presents a revised global estimate for organic carbon burial in seagrass meadows, based on 222 previously-published sediment cores from around the world. The authors summarize sediment accumulation rates, percent organic carbon and organic carbon burial rates by region and include both vegetated and unvegetated sediment. They use these data to determine important geochemical relationships on a global scale, including the relationship between % organic carbon and sediment accumulation rate. They conclude that the previous global estimate was too high and revise it downwards, by a factor of four, to 6-16 Tg C/yr. Furthermore, they have compiled the data into an open source data set.

Major comments

This paper represents a significant advance from earlier global seagrass carbon burial estimates, which were based on 6 sediment cores from one region. The authors have directly addressed some of the other problems common to previous papers as well. In particular, they have addressed the difference between carbon stock and carbon burial, and determined burial rates for different genera of seagrass. The paper is very well-presented, with clear and useful figures, and the open-source data set will be valuable to other researchers.
However, two major problems from earlier estimates still remain.

Thank you for the careful reading and for highlighting the strengths of the manuscript and the effort involved in compiling this dataset.

The dataset has been uploaded to Figshare with the private link: https://figshare.com/s/bda2144e873b0bf9983c

#1) The most significant is that their carbon burial rates include allochthonous carbon. Williamson et al. (2025. Global Change Biology 31(4):e70181) showed, based on a global data compilation, that the median contribution of allochthonous carbon to total carbon burial in seagrass meadows is 74%. About three-quarters of the carbon buried in seagrass meadows comes from elsewhere, mainly from land, and would likely have been buried in coastal sediments, regardless of the presence or absence of the seagrass meadow. Allochthonous material cannot be attributed to the seagrass meadow. Since the proportion of allochthonous carbon is not always reported, the authors probably do not have access to this information for their whole data set. In such cases, Williamson et al suggested that a default value be used, based on the global median.

I recommend that the authors reduce their global seagrass carbon burial rate by 74% to account for this.

Our perspective is that the distinction between autochthonous and allochthonous OC is not always straightforward. Autochthonous primary production (organic matter generated within an ecosystem) in seagrass meadows is not limited to the seagrass plants themselves. Microalgae and macroalgae are part of the system and cannot be rejected as being autochthonous, as these are important components to NPP in seagrass meadows, sometimes even more than seagrass themselves. The only sources that can unambiguously be considered allochthonous are terrestrial and mangrove/salt marsh carbon.

Whether terrestrial allochthonous OC should or not be accounted for remains an active topic of debate (Houston et al. 2025; Williamson et al. 2025, or Lovelock et al. 2023). However, it is outside the scope of this manuscript to make prescriptions about accounting methodologies. Ultimately, decisions on whether blue carbon accounting frameworks should apply deductions to burial rates should be up to organizations formulating standards and methodologies. Regarding this topic, we note there are inconsistencies among methodologies and between methodologies and current IPCC guidelines. For example, current IPCC greenhouse gas inventory guidelines do not require the partitioning of autochthonous and allochthonous OC, and restoration assessments generally rely on total organic carbon accumulation rates. As such, we consider it important to report total OC burial rates, as it allows guideline developers to apply the reduction factors they consider appropriate based on their methodological approach and evolving knowledge of allochthonous OC burial.

Likewise, we believe it is valuable to quantify how much seagrass-derived OC is retained in sediments in order to better constrain seagrass carbon budgets (including export terms), therefore, in the revised version of the manuscript we provide an estimate of seagrass-derived OC burial based on δ^{13}C data for those who might be interested in this value either for carbon cycle research or accounting frameworks.

To estimate the potential contribution of seagrass-derived OC in sediments we used the seagrass-sediment Δ^{13}C offset (for details on definition see response to comment #9) which across the synthesized data in vegetated sediments was of +6.1± 1.0‰. This value is similar than that found in the global synthesis by Kennedy et al (2010) of +6.4 ± 3.4‰, which applying a one isotope mixing model with 3 potential organic matter sources (seagrass, phytoplankton, and mangroves/terrestrial organic matter) found that seagrass-derived organic matter contributed a median of 51% (IQR 33-62%) of total sediment OC.

We used this value rather than the 74% estimate or site-specific values because it was compatible with our $\Delta^{13}C_{seagrass\text{-}sediment}$ and because endmember values were not available for each core site. In addition, compiling estimates of seagrass contributions reported in individual studies, as was done to derive the 74% estimate, would likely yield non-comparable results unless endmembers were harmonized across studies. Mixing model outputs depend strongly on the endmembers used to define the iso-space, including the number and type of sources and tracers considered, which vary substantially among studies. For this reason, we relied on the estimate of Kennedy et al. (2010), which was derived by applying the same mixing model and a consistent set of endmembers across all studies.

In the revised version of the manuscript, we now report range and median values for $\delta^{13}C$ in seagrass and sediments across the synthesis and establish a comparison with the global value found in Kennedy et al. (2010) in section "*Interplay between organic carbon content and sedimentation rates*".

L268-273: *"Sedimentary OC exhibited $\delta^{13}C$ values ranging from -10.8 to -26.9‰, which, relative to seagrass isotopic signatures (-5.2 to -15‰), resulted in seagrass-sediment isotopic offsets ranging from 0.02 to 23.0‰ (median: +6.1 ± 1.0‰). The median isotopic offset is similar to the global offset of +6.4 ± 3.4‰ by Kennedy et al.[16] , who inferred that seagrass-derived organic matter contributed a median of 51% to sediment OC, with remaining contributions from phytoplankton and terrestrial organic matter."*

Then in section "*Global magnitude of organic carbon burial in seagrass sediments*" we provide a burial rate estimate of seagrass-derived OC"

L457-462: *"Assuming that roughly half of the total OC buried in seagrass sediments is from seagrass material[17], the corresponding burial rate of seagrass-derived OC would be 17 ± 4 g OC m-2 yr-1, suggesting that ~15% of the NCP is retained and buried in situ within meadows, while the remaining ~85% may be redistributed to adjacent shorelines, unvegetated sediments, and offshore areas[56,61–63,] where it could be buried, remain dissolved, or return to the atmosphere as CO_2."*

Additionally, we have revisited the terms allochthonous and autochthonous across the manuscript.

Finally, we have rewritten the last section of the manuscript to clarify how the different OC burial rate estimates should be interpreted according to their intended applications, where we include the treatment of allochthonous terrestrial OC. We also emphasize that the climate-relevant metric is the change in burial rates rather than their absolute magnitude, as what ultimately matters is the direction and magnitude of change relative to a baseline

condition. Therefore, under accounting frameworks based on net additional storage, considerations such as source attribution become secondary to the net change in carbon sequestration resulting from management actions.

We have not reproduced the full revised section here because of its length (L485-533). Instead, we include the paragraphs most relevant to this comment.

L486-496*: "Challenges and pitfalls of utilizing OC burial rate estimates in blue carbon ecosystems for climate change mitigation have been described elsewhere[23,65], being long-term permanence and the attribution of OC source, particularly the treatment of allochthonous inputs, dominant topics of debate [66–68]. These discussions highlight the necessity of clarifying the interpretation and appropriate application of the global estimates presented here. The rates reported in this study represent data-constrained estimates of total OC burial in seagrass sediments integrated over a centennial timescale. They do not provide a direct measure of climate mitigation; rather, they are reference values describing contemporary OC burial within seagrass meadows. As such, they can serve as baseline conditions prior to habitat degradation, or as target conditions following restoration when site-specific measurements are unavailable."*

L513-528*: "Sediment OC source attribution is commonly based on stable isotope mixing models, as seagrass $\delta^{13}C$ is typically enriched relative to terrestrial, mangrove, salt marsh, and plankton organic matter sources [70,71]. However, inferred contributions are highly dependent on endmember selection, and emerging approaches such as compound-specific isotopes and eDNA offer improved source resolution [72]. It is important to clarify that the distinction between autochthonous and allochthonous OC refers to within-ecosystem production versus external inputs, rather than a simple seagrass versus non-seagrass dichotomy. Accordingly, both seagrass and other in situ primary producers (e.g., micro- and macroalgae) are autochthonous, whereas terrestrial, mangrove, and salt-marsh inputs are allochthonous. The OC burial rates reported here represent total sediment OC burial, integrating all sources and represent the net outcome of OC gains and losses over a century scale. The partitioning of autochthonous versus allochthonous OC remains debated but is not required under major accounting frameworks. For instance, IPCC greenhouse gas inventory guidelines and wetland restoration assessments typically rely on total OC burial without requiring source partitioning. Consequently, under accounting frameworks based on net additional storage, explicit resolution of source attribution and long-term OC cycling processes may be secondary to quantifying the net change in OC burial resulting from management actions. Ultimately, the relevance of these OC burial rates depends on the specific biogeochemical, carbon cycle, or policy context in which they are applied."*

#2) The authors do not appear to have accounted for remineralization of organic carbon in surface sediment. (If they have, then they need to clarify this point.) They used previously-published organic carbon burial rates or recalculated the rates based on existing data. They calculated the burial rates as the product of the mass accumulation rate and the fraction of organic carbon, which is correct. However it appears that they used the fraction of organic carbon all the way from the surface to the bottom depth of excess Pb-210 (Line 545). The % organic carbon at the surface is higher than the % buried, because benthic animals and bacteria consume organic carbon in surface sediments, often down to a depth of 20 cm or more. A carbon burial rate calculated using the surface % organic carbon is too high. This error is not as large as that from the inclusion of the allochthonous material, but it does artificially inflate the carbon burial rate. This point is explained in detail in a recent methods paper (Johannessen, 2023. Carbon Footprints 2(21): DOI: 10.20517/cf.2023.37) The methods paper does not necessarily need to be cited in the revised manuscript; I only include it here for the authors' information.

I suggest that the authors recalculate their carbon burial rates, using only the % organic carbon from the deepest sections of the cores, and excluding the surface. With such a well-organized data synthesis, it should not be too difficult to recalculate the rates. An alternative would be to calculate the ratio of the % organic C at 20 cm or greater to that at the surface for as many cores as possible and then reduce their global rate by that factor.

Surface OC may be transient on millennial timescales, but in healthy seagrass ecosystems it can be considered part of a dynamic steady state, as it is continuously replenished through ongoing production and burial. The production and burial of potentially labile OC is relevant to the ~100-year timescale captured by our burial rate estimates, as it represents carbon transferred from ocean–atmosphere reservoirs into sediment, and could be understood as a buried standing stock like biomass is considered in many instances (Lovelock et al., 2022, Baker et al 2014, IPCC, 2014).

In our study, values of OC content from the sediment surface to the depth of the excess ^{210}Pb horizon were included in the calculations. Across the dataset, this horizon generally occurred around 25 cm depth, consistent with the mean sedimentation rate estimated in this study (2.4 mm yr^{-1}; 95% CI: 2.1–2.6 mm yr^{-1}), such that the upper ~25 cm of sediment broadly represents the last century of accumulation. Including OC concentrations below the excess ^{210}Pb horizon would extend beyond the temporal window of study and is not representative of the timescale integrated here. Restricting OC burial concentrations to the deepest sampled intervals would also be inappropriate, as it would combine contemporary sedimentation rates with OC values from sediment layers dating back several centuries to

millennia when depositional conditions were potentially different. We chose to focus on OC burial rates over centennial rather than a millennial timescale, as this timescale is highly relevant for management actions and restoration project life cycles.

But we understand the concern raised regarding early OC loss in surface sediments and its potential influence on OC burial rate estimates. To evaluate this across the dataset, we examined sediment OC profiles from all seagrass cores for which OC burial rates could be derived. For each core, and as a first approximation to illustrate the concept, we fitted a linear regression between OC concentration and depth over the ^{210}Pb-derived chronostratigraphic interval and classified profiles based on the sign of the slope and p-value ($\alpha = 0.05$). We found that 51% of profiles exhibited no significant trend with depth, 39% showed decreasing OC concentrations, and 10% displayed increasing OC content with depth (Fig. R1, below; not included in the manuscript). These results indicate that OC profiles over ^{210}Pb timescales do not uniformly show progressive degradation of OC, but represent various processes including apparent steady-state accumulation, net decreases in OC consistent with degradation, and increasing OC trends depending on environmental context. This is not new to our dataset and has been described in other works like Piñeiro-Juncal et al (2025).

To further quantify the magnitude of downcore OC change, we calculated the ratio between mean OC concentration in the bottom 3 cm and the uppermost 3 cm of each profile within the dated layers. Decreasing profiles exhibited a mean bottom-to-top OC ratio of 0.44. In contrast, increasing profiles exhibited a ratio of 1.82, while stable profiles showed a ratio close to unity (0.90). The median bottom-to-surface OC concentration ratio pooled across all vegetated sediment cores was 0.70 over a centennial timescale. This suggests that, on average, OC concentrations decrease by approximately 30% from the sediment surface to the deepest dated layers, although the magnitude of this decline varies strongly among sampled seagrass meadows. We report this factor in the Results section of the revised manuscript for transparency and for use by readers who wish to account for potential downcore variation in OC concentrations. In the Methods, we provide details of its derivation.

The Results Text now reads **L222-228**: *"In contrast, our analysis captures the mean in situ accumulation of OC in seagrass sediments over the last century, excluding export and burial beyond the meadows, sequestration in biomass, and short-term net ecosystem uptake. While our estimates include surface sediment OC that may not have undergone complete decomposition, this contribution was small, although variable. Over a centennial timescale, 51% of vegetated sediment profiles had stable OC concentrations with depth,*

39% decreased, and 10% increased, yielding a median bottom-to-surface OC concentration ratio of 0.70."

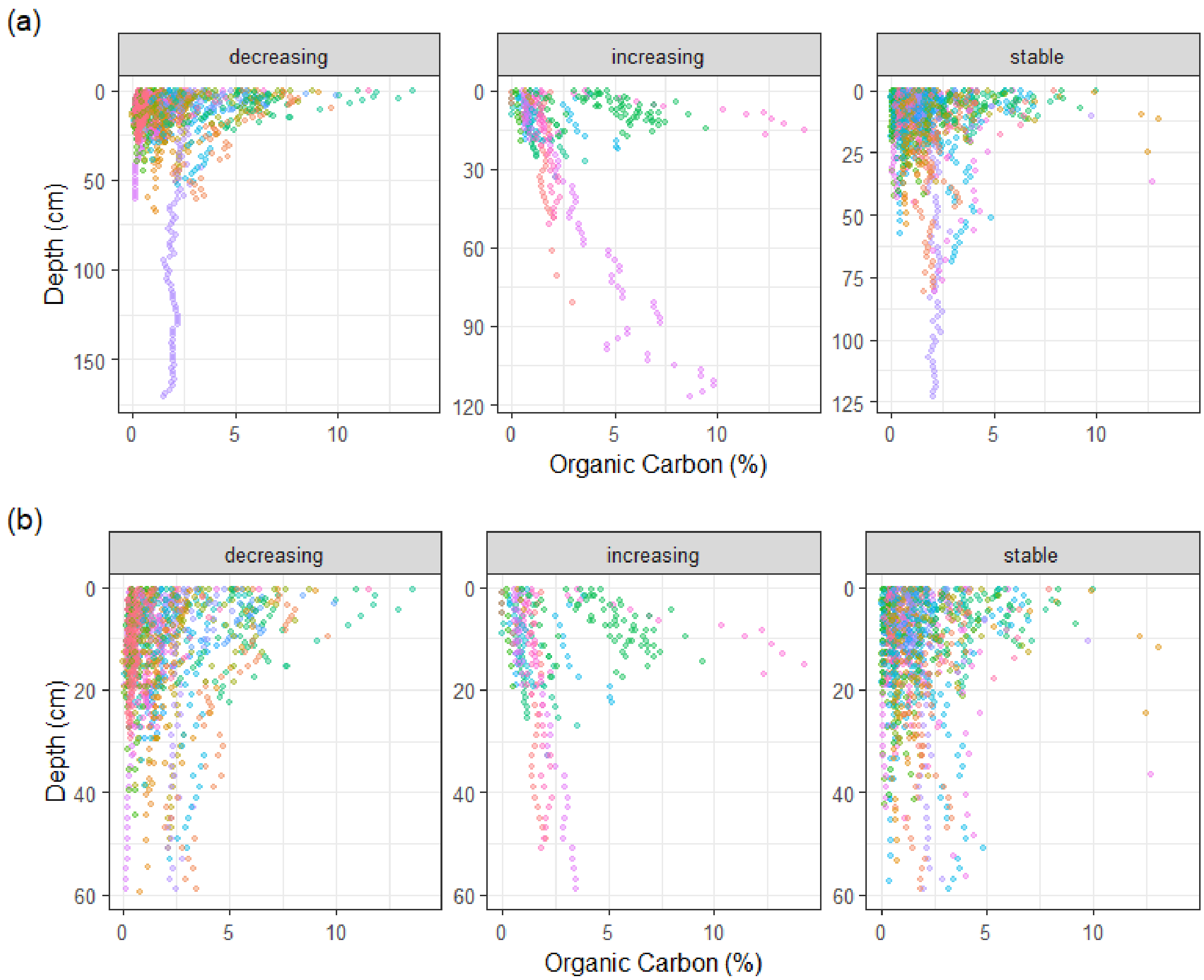

Figure R1. Organic carbon depth profiles.(a) Profiles shown over the excess ^{210}Pb horizon (i.e., the practical dating limit as per excess ^{210}Pb). Deepest age-depth limits are for cores in Florida Bay Banks and Swan Lagoon. (b) Profiles shown over the upper 60 cm to improve visualization of shorter cores.

New text In Methods **L615-632**: "*Recent studies have suggested using stabilized OC concentrations at depth to minimize the effect of labile OC that has not undergone decomposition in burial rate estimates [23,69]. To evaluate this effect across the dataset, we examined sediment OC profiles from all seagrass cores for which OC burial rates could be derived. For each core, we fitted a linear regression between OC concentration and depth over the ^{210}Pb-derived chronostratigraphic interval and classified profiles based on the sign*

of the slope and p-value ($\alpha < 0.05$). As a result, 51% of profiles exhibited stable OC concentrations with depth, 39% showed decreasing OC concentrations, and 10% displayed increasing OC content with depth. To quantify the magnitude of downcore OC change, we calculated the ratio between mean OC concentration in the bottom 3 cm and the uppermost 3 cm of each profile within the dated layer. Decreasing OC profiles exhibited a mean bottom-to-top OC ratio of 0.44. In contrast, increasing profiles exhibited a ratio of 1.82, while stable OC profiles showed a ratio close to unity (0.90). When all vegetated sediments were pooled together, the median bottom-to-top OC factor was 0.70. This indicates that although some decomposition occurs on centennial timescales, the magnitude of the resulting decline varies strongly among seagrass meadows, and therefore, we did not apply a remineralization factor to the global OC burial rate estimates. If applied, it would yield rates similar to our most conservative estimate, which includes sites with negligible recent accumulation (20 g OC m^{-2} yr^{-1}; 95% CI: 15–23).”

However, we have not applied this factor for a couple of reasons. First, evidence of OC decrease with depth did not occur in the majority of the cores across the dataset. Second, if applied, it yields rates similar of our most conservative estimate, which includes sites with negligible recent accumulation (20 g OC m^{-2} yr^{-1}; 95% CI: 15–23), thus all considered our main result would not vary considerably.

Centennial-scale seagrass OC burial should be interpreted within the context of contemporary carbon cycling, rather than as a mechanism for offsetting the large transfer of geologic carbon to the atmosphere through fossil fuel combustion. We have revised the final section of the manuscript, “Constraints on interpreting OC burial rates,” to clarify how these estimates should be interpreted. Please see the full section (L485–533). Pasting below the paragraph most relevant to this comment

L504-514: *“If OC burial rates are used in the context of climate mitigation, the primary consideration is the timescale of interest. Within the contemporary carbon cycle, the climate-relevant metric is the net change in burial fluxes resulting from ecosystem degradation, conservation, or restoration, rather than the baseline absolute OC burial rates reported here. If the objective is to quantify long-term carbon preservation over centuries to millennia, accounting for the remineralization of labile surface OC and long-term sediment accumulation rates become more relevant. These long-term dynamics can be constrained by assessing stabilized OC concentrations at depth[69] alongside with longer-term chronologies (e.g., ^{14}C). However, such approaches raise additional questions regarding historical environmental conditions, including whether seagrass ecosystems were present during that period.”*

References

Baker, K., Baumann, D.,Michaud, J., and van Mossel-Forrester, B. (2014). Technical specification: mixed species forest plantation. Registered Plan Vivo project: The CommuniTree Carbon Program (CommuniTree). https://www.clevel.co.uk/wp-content/uploads/2022/05/CommuniTree_PDD.pdf.

IPCC. 2013 Supplement to the 2006 IPCC Guidelines for National Greenhouse Gas Inventories: Wetlands Task Force on National Greenhouse Gas Inventories: Wetlands. (IPCC, Switzerland, 2014).

Lovelock, C.E., Adame, M.F., Bradley, J., Dittmann, S., Hagger, V., Hickey, S.M., Hutley, L.B., Jones, A., Kelleway, J.J., Lavery, P.S., et al. (2022). An Australian blue carbon method to estimate climate change mitigation benefits of coastal wetland restoration. Restor. Ecol. 31, 1–15. https://doi.org/10.1111/rec.13739.

Piñeiro-Juncal, N., Mateo, M.Á., Leiva-Dueñas, C. et al. Soil organic carbon depth profiles and centennial and millennial decay rates in tidal marsh, mangrove and seagrass blue carbon ecosystems. Commun Earth Environ 6, 504 (2025). https://doi.org/10.1038/s43247-025-02390-2

I recommend that the paper be accepted with major revisions.
Detailed comments

#3 Line 55. Change “are” to “have been,” since this paper overturns the previous value.

Changed as suggested

#4 Lines 115-116. Add a reference for the Tier 1 default value.

Reference added as suggested

#5 Figure 1. This is a useful figure. It illustrates the broad coverage of this data set, and the inset provides useful context about the latitudinal distribution of samples.
Thank you. In response to a comment from Reviewer 1, we have slightly modified the inset to report the percentage of cores per latitude bin rather than raw core counts, as it is more informative for spatial bias interpretation.

#6 Lines 139-141. Although these values were published, given the widespread errors in

carbon burial calculation, some (or many) of them might be incorrect, mainly too high. Did the authors assess the validity of the published rates where possible?

Our way to apply quality control to published OC burial rates was through classifying ^{210}Pb depth profiles using the following flags: (1) ideal, (2) surface mixing, (3) intense mixing (≥50% of the excess ^{210}Pb profile was mixed), or (4) negligible excess ^{210}Pb (column Pb_210_profile_flag in dataset) as the larger uncertainties in burial rate estimates come from the dating. For cores exhibiting negligible excess ^{210}Pb, OC burial rates were considered not different from zero and were assigned a value of zero regardless of the published value. Additionally, we compared published fluxes with those calculated using Eq. 1 in the manuscript, and when discrepancies were large without clear justification (e.g., estimates derived for specific time periods rather than the last century), we used the recalculated values instead of the published ones. The values that were used in each core can be tracked in the summary_results_table where we record published, calculated, and used values.

We then evaluated the influence of the ^{210}Pb profile quality on our results by calculating the global estimates both including and excluding cores flagged as exhibiting intense downcore mixing. We did not identified significant differences in the geometric mean OC burial rate when excluding rates derived from highly mixed profiles (Table S1), and this is in part due to the geometric mean being less sensitive to outliers and extreme values. The cores flagged as having intense downcore mixing could bias the OC burial rate high, as it is not reasonable to fit a model below the mixed layer if the mixed layer extends to >50% of the profile. However, the comparison showed that this was not biasing global estimates high and allowed us to evaluate the robustness of our estimates to potential biases associated with profile quality.

We also evaluated the global OC burial rate estimate including cores assigned a zero OC burial rate and reported the global median across the dataset providing a lower bound for the sedimentation and OC burial estimates.

In this sense we consider that our OC burial rate estimate is generally conservative, but this doesn't mean some published estimates might be incorrect or biased high. Data synthesis is a great method to evaluate the consistency of reported values across studies and to identify observations that fall at the extremes of the global distribution. In the example described in L139-141 the reported lower and upper bounds of 1.3 and 976 g OC m^{-2} yr^{-1} belong to cores with a Pb-210 profile flag of 3 (intense downcore mixing) and, as shown in Fig. 2, the upper bound represents and outlier within our dataset. In the revised version of the manuscript, we explicitly identify this case as an outlier and also provide a range of OC burial rates in vegetated sediments excluding cores showing intense downcore mixing.

L147-152: *"Estimates of OC burial rates within depositional vegetated sediments spanned three orders of magnitude, ranging from 1.3 ± 0.5 g OC m^{-2} yr^{-1} (Ameralik, Greenland [37]) to 976 ± 132 g OC m^{-2} yr^{-1} (Swan Lagoon, Shandong, China[20]), the latter being a high-end outlier in the global distribution (Fig. 2a). Excluding rates from sediments exhibiting intense downcore mixing, which are subject to greater uncertainty, did not substantially reduce the range, which still spanned two orders of magnitude (3.6–314 g OC m^{-2} yr^{-1}; Table S1)."*

Additionally, we have clarified the ^{210}Pb profile quality flag assignment in the Methods section; this information was previously included but was somewhat "buried" within the list of collated variables.

Now text reads

L589-592: *"We assigned ^{210}Pb profile quality flags to all cores in the database for quality control purposes classifying each profile as (1) ideal, (2) exhibiting surface mixing, (3) pervasive downcore mixing, or (4) negligible excess ^{210}Pb following Arias-Ortiz et al.[12]."*

L602-610: *"We compared published OC burial rates with first-order recalculated estimates and used standardized residuals (> |2|) to identify unusually large discrepancies. Where these discrepancies could not be explained by methodological differences (e.g., time-specific versus centennial-scale estimates), we retained the recalculated rates to ensure methodological consistency across the dataset. Published, calculated, and used OC burial rates can be found in the summary_results_table accompanying the dataset in Arias-Ortiz et al.,[31]. Methods documentation such as ^{210}Pb quality flags, compaction percentages, and OC analytical methods, are provided in the depth-series dataset table[31]."*

L642-646: *"Cores exhibiting intense downcore mixing (^{210}Pb profile quality flag 3) for which OC burial rates were reported (26% of cores) were included in the main analyses. However, global burial rates were computed with and without these values to assess the potential influence of ^{210}Pb profile quality on OC burial estimates, particularly the extent to which downcore mixing may bias rates high".*

#7 Figure 2. This is a useful figure that presents a lot of information clearly.

Thanks. In response to Reviewer 3's suggestion, we have slightly modified the figure by adding a break in the x-axis of the histogram to improve clarity. We have also swapped panels A and B to match their order of appearance in the text.

#8 Figure 3a. Enlarge the main panel of this figure, so that the legend will fit inside. I missed the legend at first.

Thanks, we have enlarged the main panel of this figure and increased the legend font size. We have kept the legend above the panel, as placing it inside the plot made the figure appear too cluttered.

#9 Lines 277-278 and Figure 4. First mention of seagrass-sediment isotopic offset. How was this calculated? What is the range of dC-13 values for seagrass? For sediment? Explain why this parameter was used, what it means and why it might vary with water depth. How do these values compare with those of particulate matter from nearby terrestrial runoff or phytoplankton? Why does there seem to be a larger seagrass-sediment difference in areas of low carbon accumulation? Include a reference about the use of stable isotopes to identify the origin of organic matter in a seagrass meadow.

It is known that the seagrass $\delta^{13}C$ signatures are isotopically heavier than those of other organic matter sources in the coastal zone, such as terrestrial OM sources, mangroves, tidal marshes, and plankton (Kennedy et al 2004, Fry et al 1977). From our dataset, the $\delta^{13}C$ seagrass ranged from -5.2 to -15‰ (mean ± SD: -10.5 ± 2.1‰), and the sediment was of -10.8 to -26.9‰ (mean ± SD: -17.0 ± 3.8‰) across cores showing net positive accumulation.

The comparison with values from particulate OM from nearby runoff or phytoplankton is not feasible within our synthesis because these endmembers were not consistently reported across the studies, and the endmembers considered in each study also varied case by case, which makes data standardization not possible. Additionally, the location of the sampled seagrasses and cores relative to potential allochthonous sources (e.g., mangroves, marshes) was also not consistently reported. Given this limitation, we instead used the difference between seagrass and sediment $\delta^{13}C$ values ($\Delta^{13}C = \delta^{13}C_{seagrass} - \delta^{13}C_{sediment}$) as a proxy of the degree to which sediment OC differs isotopically from seagrass-derived OC. Values of $\Delta^{13}C$ approaching 0‰ indicate that sediment and seagrass $\delta^{13}C$ signatures are similar, suggesting that most of the sediment OC may be derived from seagrass matter.

We did not observe a relationship between the seagrass–sediment isotopic offset and water depth across the dataset (Fig. R2a, not included in the manuscript). Therefore, we have not included such a discussion in either the original or revised manuscript. The larger seagrass-sediment difference (i.e., $\Delta^{13}C >> 0$) in areas of low OC accumulation may be associated to environments characterized by limited fine-sediment deposition (Fig. R2b, not included in the manuscript) low sedimentation rates, and a potentially low contribution of seagrass derived organic matter to sediment OC. Seagrass cover/biomass data were not available for most cores, and would not be directly interpretable at the centennial

timescale, since biomass is likely to have changed over time. However, these results combined suggest that sediment $\delta^{13}C$ values closer to those of seagrass are associated with sites where OC burial is intermediate to high.

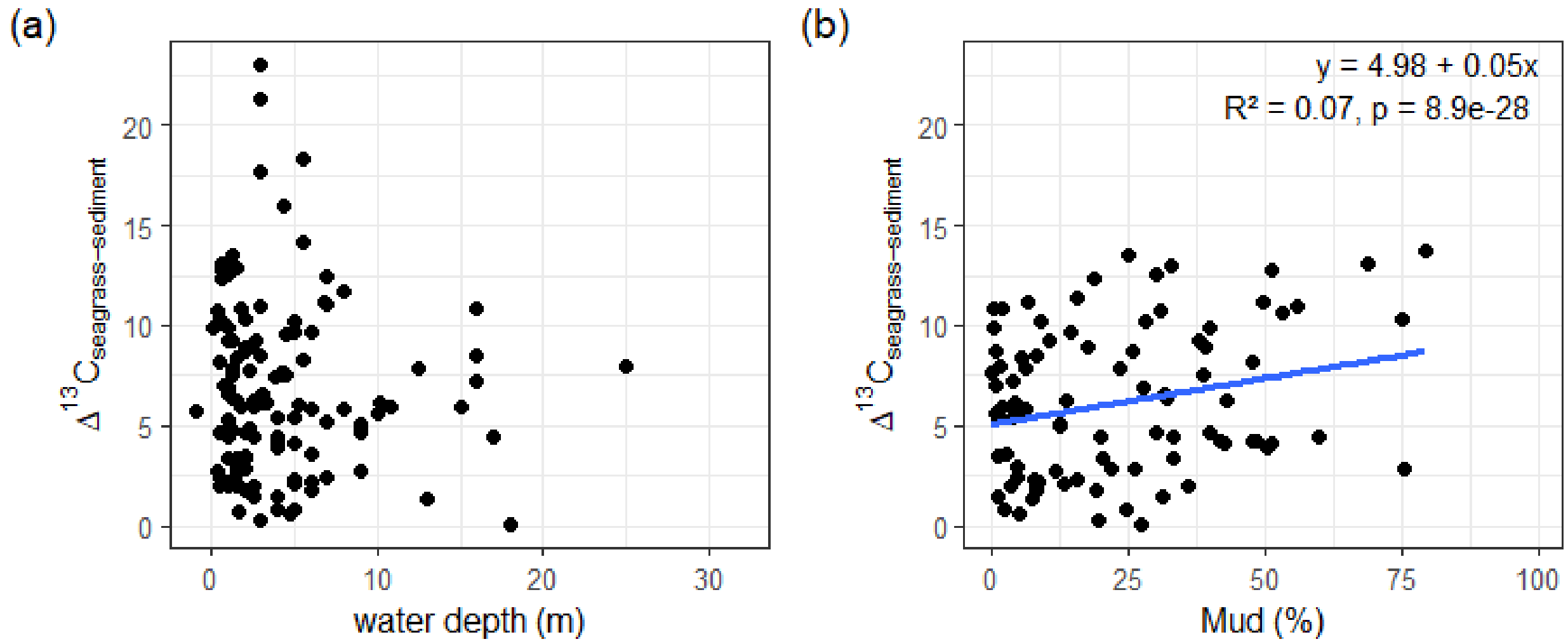

Figure R2. Seagrass–sediment isotopic offset as a function of water depth (a) and as a function of sediment mud content (b).

Based on this discussion, we made the following changes to the manuscript:

We have added text in the Main introduction to highlight that $\delta^{13}C$ signatures were also collated, so they are introduced earlier in the manuscript and do not appear as a new element later on.

L114-116: "*Our analysis integrates downcore sediment profiles of OC content, their d^{13}C signatures, and ^{210}Pb specific activities along with ancillary variables and relevant metadata, resulting in a comprehensive, open-source dataset* [31]."

Then in section "*Interplay between organic carbon content and sedimentation rates*" where the seagrass-sediment isotopic offset results are first presented, we now also report the $\delta^{13}C$ signature ranges for seagrasses and sediments and their interpretation.

L268-273: "*Sedimentary OC exhibited $\delta^{13}C$ values ranging from -10.5 to -26.9‰, which, relative to seagrass isotopic signatures (-5.2 to -15.3‰), resulted in seagrass-sediment isotopic offsets ranging from 0.02 to 23.0‰ (median: +6.1 ± 1.0‰). The median isotopic offset was similar to the global offset of +6.4 ± 3.4‰ by Kennedy et al.* [16] *, who inferred that seagrass-derived organic matter contributed a median of 51% to sediment OC, with remaining contributions from phytoplankton and terrestrial organic matter.*"

L303-306: *"In contrast, low burial rates were associated with environments characterized by low sedimentation and a potentially low contribution of seagrass organic matter to sediment OC, reflected in a larger offset between seagrass isotopic signals and that of sediment OC compared to high-burial settings (Fig 4c-e)."*

Finally, we have included a new paragraph in the Methods section to explain how $\delta^{13}C_{seagrass}$ were assigned to each core (in response to comment #14), as well as how the isotopic offset was calculated and why it was used:

Methods **L573-588**: *"Sediment $\delta^{13}C$ values were used in conjunction with seagrass $\delta^{13}C$ signatures to assess the influence of seagrass organic matter on sedimentary OC[17]. Seagrass $\delta^{13}C$ signatures were assigned to each core using the most site-specific information available. Where species-specific $\delta^{13}C$ values were reported at the site, these were matched to the seagrass species present at the core location, otherwise, site-level mean seagrass $\delta^{13}C$ values were used, which could include multiple species. Mean site-level seagrass $\delta^{13}C$ values were also assigned to unvegetated cores collected within the same site. Bare sediment cores that did not share a Site ID with vegetated cores were not assigned a seagrass $\delta^{13}C$ endmember (13 out of 37). Because seagrass $\delta^{13}C$ signatures are isotopically heavier than those of other coastal organic matter sources (e.g., terrestrial vegetation, mangroves, tidal marshes, and plankton)[70,71], the difference between seagrass and sediment $\delta^{13}C$ values ($\Delta^{13}C = \delta^{13}C_{seagrass} - \delta^{13}C_{sediment}$) was used as a proxy for the presence of seagrass versus non-seagrass sediment OM. This approach was chosen because non-seagrass endmembers and their classification as internal or external sources to the ecosystem were not consistently reported across studies, and terrestrial allochthonous inputs were not always sampled, precluding cross-study comparisons and motivating the $\Delta^{13}C$ harmonized approach".*

References included were:

Kennedy, H. et al. Seagrass sediments as a global carbon sink: Isotopic constraints. Global Biogeochem. Cycles 24, 1–9 (2010).

Kennedy, H., Gacia, E., Kennedy, D. P., Papadimitriou, S. & Duarte, C. M. Organic carbon sources to SE Asian coastal sediments. Estuar. Coast. Shelf Sci. 60, 59–68 (2004).

Fry, B., Scalan, R. S. & Parker, P. L. Stable carbon isotope evidence for two sources of organic matter in coastal sediments: seagrasses and plankton. Geochim. Cosmochim. Acta 41, 1875–1877 (1977).

Papadimitriou, S., Kennedy, H., Kennedy, D. P., Duarte, C. M. & Marbá, N. Sources of organic matter in seagrass-colonized sediments: A stable isotope study of the silt and clay fraction from Posidonia oceanica meadows in the western Mediterranean. Org. Geochem. 36, 949–961 (2005).

#10 Lines 339-345. How is "unvegetated sediment" in a seagrass meadow defined? How close did it have to be to living seagrass, or how large was the open patch? If these unvegetated patches were very small and located in the middle of a seagrass meadow, it is not surprising that they had similar organic carbon burial rates. A seagrass frond falling over sideways on a scale of tens of cm wouldn't really qualify as being "exported" from the meadow. Clarify what these samples represent.

Most of the bare sediment cores included in the synthesis come from studies that sampled both seagrass and unvegetated areas around the same study location.

We revisited the dataset and calculated straight-line distances between unvegetated core and its nearest seagrass core at paired sites to better characterize these pairings. For the subset of studies where coordinates were available at the core level, distances ranged from 6 m to 3 km (median = 810 m).

Nevertheless, for studies reporting coordinates only at the site level (i.e., where all cores within a site share the same coordinates) (Holmes et al., 2001; Lutz, 2018; Samper-Villareal et al., 2022; Greiner et al., 2013; Kaewsrikhaw et al., 2026), information on the original manuscripts suggests that cores were generally collected several meters away from a vegetated area. For example, Holmes et al. (2001) reports that bare core 6C was collected 10 m from vegetated core 6A, and Lutz (2018) reports that bare cores were collected at least 30 m from vegetated areas. The remaining studies do not provide information on the location of unvegetated sediments relative to vegetated sediment cores, but we expect them to fall within a similar range.

The synthesized studies do not report how large the bare areas sampled were, so unfortunately, we cannot track down that information.

We have clarified what unvegetated sediments represent in the introduction of the manuscript and have revised the term "export" as comparisons between seagrass and adjacent unvegetated sediments are probably better described as lateral redistribution rather than export off-ecosystem.

L123-126: *"The dataset comprised 332 sediment profiles from seagrass ecosystems across a latitudinal range of 64ºN to 35ºS (Fig. 1), including vegetated (89%) and unvegetated*

(11%) sediments. Unvegetated cores represented adjacent non-vegetated areas generally paired with nearby seagrass cores, separated by distances ranging from 6 m to 3 km".

L405-407: *"Local sedimentation dynamics, together with seagrass presence and lateral redistribution of seagrass OC across vegetated–unvegetated boundaries[16,54,] can therefore result in similar OC burial rates between seagrass and adjacent bare sediments[40]."*

#11 Line 355, line 358. The sentence includes both "although colonized by seagrasses" and "irrespective of seagrass cover." Clarify. It would probably help to break this into two sentences.

The sentence was split as suggested.

Text now reads **L381-385**: *"Across the dataset, sediments in non-depositional environments had significantly higher sediment dry bulk densities (DBD: 1.3–1.5 g cm^{-3}) and lower OC content (0.30–0.50%), even in areas colonized by seagrasses. In contrast, depositional environments, both vegetated and unvegetated, exhibited lower DBD and higher OC content (DBD: ~0.9–1.2 g cm^{-3}; OC: 0.8-1.3%) (Fig. 5b)."*

#12 Lines 359-361. Explain how the isotopic offset indicates the contribution of autochthonous and allochthonous sources.

The isotopic offset indicates how similar the sediment OC is to the seagrass-derived OC material, based on their isotopic $d^{13}C$ signatures, thus, similar offsets across sites may indicate a comparable degree of seagrass influence on sedimentary OC. However, the isotopic offset doesn't provide with proportional source contributions. We have rephrased the sentence and now it reads:

L385-389: "*Despite differences in sediment characteristics between depositional and non-depositional habitats, the isotopic offset between seagrass and sediment OC ($\Delta^{13}C_{seagrass-sediment}$) was similar across both habitats (Fig. 5b), pointing to a shared influence of seagrass-derived material on sedimentary OC, even if their capacity to accumulate and retain OC differs."*

#13 Lines 362-363. The similarity among isotopic offsets in vegetated, non-vegetated, depositional and non-depositional environments might alternatively mean that seagrass carbon is not a significant contributor to the sediment in any of these settings… Did the authors determine the proportional contribution of seagrass carbon in the various environments?

We could not determine the proportional contributions of seagrass versus allochthonous sources because, as noted in Responses #1 and #9, endmember information was not consistently reported across studies. However, these isotopic offsets were similar to those reported by Kennedy et al 2010 (+6.1± 3.4‰) which determined a likely contribution seagrass-derived organic matter to sediment OC of 51%.

We have revised the text to avoid any implication of quantitative source partitioning, which is not supported by the available data. The revised text now reads:

L390-404: *"At the local scale where seagrass meadows and adjacent bare sediments were sampled within the same sites, OC content and dry bulk density differed between habitats, whereas isotopic similarity to seagrass-derived organic matter, sediment accumulation and OC burial rates did not (Fig. 5c). Although limited in scope, these findings suggest that, within depositional environments, the presence or absence of seagrass at a given location and time may not be a strong determinant of in situ OC burial rates but may have a larger effect on OC stocks. Seagrasses may modify sediment properties locally and enhance OC preservation[53] but in depositional environments, they may not alter net OC burial fluxes at the same spatial scale. Rather, seagrasses may influence burial at the site level by contributing organic matter to nearby unvegetated sediments, as evidenced by their similar isotopic signatures of seagrass-derived OC[16] (Fig. 5c). This is consistent with findings from the Bahamas[41] or Bermuda [54], where seagrass-derived OC is redistributed across the depositional seascape regardless of seagrass abundance at a given location. In Bermuda, seagrass loss has been shown to reduce the regional pool of seagrass-derived OC across both vegetated and unvegetated areas[54]."*

#14 Figure 5. How was the seagrass-sediment isotopic parameter determined for unvegetated sediment, where there was presumably not any seagrass to measure?

The seagrass isotopic endmember for unvegetated sediment cores was determined based on the mean $\delta^{13}C$ of the seagrass available at the site-level. Where bare sediment cores shared a Site ID with vegetated cores (24 out of 37), the mean $\delta^{13}C$ of the seagrasses measured within the site was used as the seagrass endmember to compare against the $\delta^{13}C$ signal of bare sediments. Bare sediment cores that did not share a Site ID with vegetated cores were not assigned a seagrass $\delta^{13}C$ endmember. We have clarified this in the methods section:

L575-581: *Seagrass $d^{13}C$ signatures were assigned to each core using the most site-specific information available. Where species-specific $d^{13}C$ values were reported at the site, these were matched to the seagrass species present at the core location, otherwise,*

site-level mean seagrass $\delta^{13}C$ values were used, which could include multiple species. Mean site-level seagrass $\delta^{13}C$ values were also assigned to unvegetated sediment cores collected within the same site. Bare sediment cores that did not share a Site ID with vegetated cores were not assigned a seagrass $\delta^{13}C$ endmember (13 out of 37)."

Figure 5 caption. Spell out DBD on first use (line 391).

Suggestion added

Lines 411-415. This sentence would be clearer with brackets around "which support extensive meadows."

Brackets added as suggested

Line 417. This global rate appears to include allochthonous carbon. See major comment #1.

The reviewer is correct, we addressed this in our response to #1.

Line 446. Change "its" to "their" or make "seagrasses" singular.

Changed "its" to "their" as suggested

Line 476. Define Tier 2 carbon accounting and provide a reference.

This line is no longer included in the current version of the manuscript, in the revised version, there is no mention of the IPCC Tier 2 approach.

Figure 6. The dashed lines for "Tier 1" and "updated" OC burial rates are too similar. Make one dotted or solid to contrast with the dashed line.

We have removed Figure 6 in response to Reviewer 1 and due to the fact that application of Tier 2 values remains premature given data limitations.

Lines 505-507. 16% of vegetated sediments were non-depositional (compared with 30% of unvegetated sediment, Figure 5), so the presence of seagrass is not a reliable indicator of carbon burial.

We agree that the presence of seagrass alone is not a sufficient predictor of carbon burial. However, our results indicate a higher likelihood of net sediment accumulation in vegetated compared with unvegetated sediments, with 16% of vegetated sites classified as non-depositional versus 30% in unvegetated settings. This difference suggests that,

although burial is not guaranteed in vegetated habitats, seagrass presence is associated with a higher likelihood of OC deposition and preservation relative to unvegetated sediments.

We have modified the text to add additional nuance

L550-552 now reads: *"Overall, the presence of seagrass is associated with more frequent depositional conditions conducive to OC burial, whereas non-depositional settings are more commonly observed in bare, unvegetated sediments"*

Line 545. What does "the fraction of OC accumulated down to the excess 210Pb horizon" mean? Were the organic carbon values averaged from the surface to the bottom? Was only the deep value used? Clarify this point. If surface or shallow (< 15 cm) values were used, then the resulting C-burial rates are too high, because of the remineralization of organic carbon in surface sediment.

We included OC concentrations from the surface down to the depth at which excess ^{210}Pb reached background levels, as this defines the dated portion of the core containing the most recent sediments (approximately the last 100 years). This is the timescale on which our manuscript and burial rate estimates are focused.

We agree that "the fraction of OC accumulated down to the excess ^{210}Pb horizon" is a bit ambiguous since it could mean the integrated OC stock, the average OC fraction, or the OC fraction at the horizon itself. We have added a clarification in the Methods section:

L612-614: *"where OC_t is the mean OC concentration of the sediments accumulated within the excess ^{210}Pb-dated interval (i.e., from the sediment surface to the depth where excess ^{210}Pb reaches background levels), and MARt is the mean mass accumulation rate over the same region."*

Line 558-559. High mixing rates only bias Pb-210-derived sedimentation rates high if Pb-210 data within the surface mixed layer are included. Those cores can give correct rates if researchers exclude the surface mixed layer and determine the sediment accumulation rate based on the rate of decay of Pb-210 below that. See Johannessen (2023) paper cited in major comment #2.

We agree, and this is why cores showing only surface mixing were retained in the calculations. In Lines L558–559 of the original manuscript (now L642-646), we describe a sensitivity analysis testing whether global OC burial estimates are biased high by the inclusion of cores exhibiting pervasive downcore mixing, rather than surface mixing. All ^{210}Pb profiles in the synthesis were assigned quality flags, two of which allow distinguishing

cores showing surface mixing (flag 2) from intense downcore mixing (flag 3). For this analysis, we excluded only cores classified as exhibiting intense downcore mixing (flag 3), as in these cases a large fraction (>=50%) of the excess ^{210}Pb profile is affected, making it difficult to isolate an undisturbed section from which to derive accurate sediment accumulation rates.

We have clarified this in the Methods section

text in **L642-646** now reads: *"Cores exhibiting intense downcore mixing (^{210}Pb profile quality flag 3) for which OC burial rates were reported (26% of cores) were included in the main analyses. However, global burial rates were computed with and without these values to assess the potential influence of ^{210}Pb profile quality on OC burial estimates, particularly the extent to which downcore mixing may bias rates high."*

Sophia Johannessen
Research Scientist, Fisheries and Oceans Canada
February 6, 2026

Reviewer #3 (Remarks to the Author):

This manuscript represents a significant update to global organic carbon burial rates in seagrass habitats, constraining earlier estimates that relied heavily on production-based approaches which may not accurately reflect actual burial rates. It also improves previous estimates by updating global surface areas of seagrass habitats, a key but somewhat overlooked factor. The use of a global sediment core database to validate existing estimates is a substantial step forward in carbon accounting within the global coastal carbon budget. The comparison between rates of sediment accumulation and organic carbon burial, and organic carbon content provide insightful analysis of the temporal, hydrodynamic and sedimentary characteristics of different global regions on seagrass carbon dynamics. I particularly appreciate the acknowledgement of estuarine geomorphological influence on organic carbon retention by seagrass habitats and agree that more analysis is warranted, albeit outside the current study scope. I found that the results were in-line with the researchers findings.

While the figures are dense and informative, they could benefit from finetuning the colors to enhance clarity. For example, the datapoints and plots in figures 4C, D, and E, and 5 could benefit from greater contrast using different colors to improve distinction. The

characteristics of presented data in Figure 2 could also be clearer if an x-axis break was added to spread out data around the Q1 and Q3 bounds to improve clarity; possibly also for figure 6. Figure 1 could also benefit from clearer marker shapes as they currently can appear as indistinct clusters of points in concentrated areas.

Thanks for the feedback, we have followed some of your suggestions and below we list the changes made to each figure, where relevant we also mention some challenges.

Figure 1: we applied transparency to the marker symbols, allowing overlapping points to remain visible while preserving their geographic position. However, given the global scale of the map and the high sampling density in some bays and coastal regions, it was not possible to completely avoid overlapping points regardless of the shape. We tested marker dispersion approaches, but the resulting displacement occupied disproportionately large areas and distorted the true spatial distribution of sampling locations.

Figure 2: An axis break was introduced between 400–850 g OC m^{-2} yr^{-1}, improving the visualization of the data distribution below 400 g OC m^{-2} yr^{-1}.

Figure 4: we have tested the use of different colors for the boxplots in panels c-e. However, adding the logCAR palette or just a discrete palette to each box resulted in a visually overloaded figure. Therefore, we opted to keep the boxplots and their data points in black to preserve clarity.

Figure 5: We consider grey and blue in panel a and b provide good contrast. So we tested the use of colors in the boxplots in panel c, but the resulting figure was confusing to our point of view, as the colors would not represent a category (e.g., bare vs. vegetated) nor a continuous scale. Therefore, we have kept the same initial colors in the figure.

Figure 6: We removed Figure 6 in the revised version of the manuscript due to the fact application of Tier 2 values remains premature given data limitations and in response to reviewer 1.

Comparisons to reported results in the literature could be clearer by explicitly quantifying the extent of differences mentioned throughout the discussion, for example in lines 104, 373, and 454.

Thanks for this comment, we agree and have added a quantitative comparison.

L104, now 109: "*These newer studies have generally reported lower burial rates that are 3–7 times lower than previously estimated for seagrasses globally*."

L454, now L478-483: *Based on this approximation, we estimate that the OC burial within vegetated coastal sediments, including mangroves, tidal marshes, and seagrasses,*

accounts for ~8 to 13% of the total oceanic OC burial, with burial in seagrass sediments representing between 3-4% using the mapped seagrass area, or 3-6% assuming a 300-600·10^3 km^2 area range; a sizeable but more modest contribution than previously assessed (~10-18%)[1] based on limited data more than a decade ago."

Line 373 in the original version has changed substantially in the revised manuscript, however we have reviewed other instances where quantitative comparisons could be made and updated them accordingly.

Additionally, regarding the complete dataset mentioned in line 552, a cross-reference would facilitate accessibility.

We have now added cross-references to the dataset in the Methods section (L608) and in the Introduction (L116), where the open-source dataset is first mentioned. As the repository is currently under private link (https://figshare.com/s/bda2144e873b0bf9983c), the DOI is not yet active but will become fully accessible upon publication.

Reference added:

Arias-Ortiz, Ariane; Lafratta, Anna; Colarusso, Phil; Fourqurean, James W; Fu, Chuancheng; Kennedy, Hilary; et al. (2026). Global dataset of seagrass sediment organic carbon burial rates and associated sediment depth-series data. Smithsonian Environmental Research Center. Dataset. https://doi.org/10.25573/serc.32592150

The method section gave a good overview of the study's approach and i did not find any major flaws. However, a summary table documenting the different methods used on different cores might enhance clarity compared to just a text-only description. However, i acknowledge that this is a personal preference given that the current description is adequate.

Together with the depth-series data, we provide a summary results table organized by Study ID, Site ID, and Core ID. This table shows the published, recalculated, and used OC burial rates for each core included in the study. More detailed methodological information, including whether sediment accumulation rates were corrected for compaction, the estimated degree of compaction, and the OC analysis method employed by original authors, is provided in the depth-series dataset available through the Coastal Carbon Network.

We have added text in the Methods section directing readers to the summary_results_table, which is unfortunately too large to be included in the main document. We hope that this addition makes it easier for readers to trace the methodological choices associated with each core.

The text added reads **L606-610**: "*Published, calculated, and used OC burial rates can be found in the summary_results_table accompanying the dataset in Arias-Ortiz et al.,[31]. Methods documentation such as ^{210}Pb quality flags, compaction percentages, and OC analytical methods, are provided in the depth-series dataset table[31].".*

Lines 572-576 reference equations but they were not presented explicitly. While in-line references were provided to the referenced studies, including the equations directly would strengthen the manuscript as a standalone study.

We have added the general equation from Fourqurean et al. (2012) for global seagrass sediments, as well as the specific equation for Florida Bay used to estimate OC content in cores from Holmes et al 2001, from where only depth series OM data was available.

L659-665: *Ten studies (56 cores) estimated OC from organic matter (OM) content using local site-specific regressions, and four studies used the general equation developed by Fourqurean et al.[19](Eq. 2). In nine cores sampled from the banks of Florida Bay[43], OC content was neither measured nor estimated, although OM content was provided. A site-specific equation (Eq. 3) published for Florida Bay[76] was applied to these sites.*

$$OC(\%) = 0.43 \cdot OM(\%) - 0.33 \quad \textit{(Eq. 2)}$$

$$OC(\%) = 0.54 \cdot OM(\%) - 0.99 \quad \textit{(Eq. 3)}$$

Other local regressions from individual studies were not applied in this work; these are reported in the original publications and were only noted as modeled estimates in our dataset. We have indicated which cores OC content is modeled vs. measured in the Methods table accompanying the dataset, but the corresponding local equations are not reported in the manuscript, as they were not directly applied in this study.

Overall, this manuscript's findings are noteworthy for publication and represent a significant advancement in our understanding of the role of seagrass habitats in organic carbon burial as part of the global coastal organic carbon budget.

Thanks so much for the positive feedback, this review was very encouraging.

REVIEWER COMMENTS

Reviewer #1 (Remarks to the Author):

I would like to thank the authors for their efforts in revising the manuscript and addressing my previous concerns. The manuscript has certainly improved and the scientific narrative is much clearer. However, before I can fully recommend this manuscript for publication in Nature Communications, there are several critical issues that still need to be addressed. general comments

Thanks for the additional feedback provided to the revised version of the manuscript. Please, see below for detailed point-by-point responses to all comments.

1. Recently published in Nature paper of 'Global high-resolution mapping of seagrass to support conservation' should be added in this MS, because authors should consider whether your work need to update the global seagrass area.

We thank the reviewer for drawing our attention to the very recently published global mapping study by Peng et al. This work has been added to the manuscript as an additional estimation of global seagrass area along the already estimates cited such as McKenzie et al 2020, Green and Short, 2003, and Jayathilake et al. 2018.

We evaluated whether its reported area should be used to update the global seagrass extent and the associated global organic carbon burial estimate presented in our manuscript. We consider our country-level areal extent still holds novelty and here are the reasons why: Peng et al. provide a valuable spatially consistent Earth Observation product describing seagrass detectable in clear, shallow coastal waters. However, the spatial domain of the analysis was restricted globally to waters shallower than 20 m, with the threshold extended to approximately 25–30 m only in selected Mediterranean regions and within 5 km of the shoreline. Seagrasses occurring beyond these thresholds were therefore excluded by design, independently of its known or potential occurrence. Moreover, seagrass in turbid waters may remain undetectable at substantially shallower depths. The resulting 148,506 km^2 consequently represents seagrass mapped within a predefined Sentinel-2-observable domain rather than a comprehensive estimate of global seagrass extent. Peng et al. themselves describe the product as a map of seagrass in clear, shallow coastal waters.

Our analysis instead uses an updated synthesis of documented country- and regional-level seagrass areas derived from multiple survey and mapping approaches and intended to

represent the broader ecological distribution of seagrasses. This synthesis produces a conservative range of 247,700–366,400 km^2 while acknowledging remaining geographical and mapping gaps. Applying a globally representative carbon-burial rate to an area restricted by the observation limits of a single satellite sensor would introduce a systematic mismatch between the ecological domain represented by the burial-rate dataset and the spatial domain represented by the area estimate.

We have therefore added Peng et al. to the manuscript Discussion as an important complementary map of shallow, optically detectable seagrass, but we have not used its reported area to replace our global extent range or to recalculate global organic carbon burial.

L432-445: *"estimates of global seagrass distribution vary widely, ranging from 148,506 to 600,000 km^2 [45,56,57], while model-based projections suggest the potential extent could be an order of magnitude higher[58]. While the lower bound is likely to be conservative[57,59], McKenzie et al.[56] synthesized expert-held occurrence and spatial polygon data to establish a revised mapped seagrass extent of 160,387–266,562 km^2, with high confidence in the lower bound and low confidence in the upper bound. We updated this compilation by reviewing country-level coverage and integrating newly published distribution data since McKenzie et al.[56] (Table S5). Based on these country- and regional-level surveys, which capture meadows over a broader ecological domain, we present an alternative global mapped area ranging between 247,700 and 366,400 km^2. This updated estimate narrows the range of uncertainty while acknowledging gaps in coverage, particularly in parts of South and East Asia (which in turn support extensive meadows), the Arabian Peninsula, the Horn of Africa, North and West Africa, and the Baltic region. Except for the Arabian Peninsula, these gaps also extend to sediment OC burial rates. "*

In the Methods:

***L713-722**: "Over a 3-fold bracket exists in the global area reported for seagrass meadows (148,506 - 600,000 km^2)[45,56,57]. The lower estimate is based on the recent pioneering work by Peng et al.[57] using a deep-learning workflow across a Sentinel-2 observable domain in clear, shallow waters (<20–30 m) within 5 km of the shore. While this represents the first comprehensive, 10m-resolution global seagrass map, it remains a conservative estimate due to optical depth and turbidity constraints. For this study, we revised the mapped seagrass area in McKenzie et al.[56] based on available polygon data and expert held occurrences, by incorporating recent country- and regional-level seagrass areas estimates derived from multiple survey and mapping approaches and intended to represent the broader ecological distribution of seagrasses (Table S5)"*

2. The authors rely exclusively on ^{210}Pb geochronology, which integrates OC burial over the past ~100 years. Ecologically, this specific century coincides with the most severe, human-induced degradation of coastal ecosystems globally (eutrophication, coastal development, dredging). The authors should explicitly discuss the "shifting baseline syndrome." Is the geometric mean of 25-33 g OC m^{-2} yr^{-1} representative of a "pristine" global seagrass sink, or does it reflect the burial capacity of already stressed, fragmented, or recovering meadows during the Anthropocene? Acknowledging that modern burial rates might be compromised by recent ecological degradation would add profound depth to the climate mitigation discussion.

We chose to focus on OC burial rates over centennial rather than a millennial timescale, as this timescale is highly relevant for management actions and restoration project life cycles.

To ensure our estimates reflect "typical" or mature contemporary seagrass sinks rather than actively degraded systems, during data compilation we excluded any sites identified as degraded, disturbed or restored in the primary literature.

But we agree with the reviewer that subtle historical shifts in seemingly "pristine" meadows cannot be entirely excluded. As detailed in the Methods (Lines XX–XX), we examined down-core OC profiles for all cores with derived burial rates and categorized them based on their depth trends into three distinct groups: stable, decreasing, or increasing OC concentrations.

We found that 51% of profiles exhibited no significant trend with depth consistent with steady-state accumulation, 39% showed decreasing OC concentrations consistent with expected diagenetic decomposition of labile OC over time, and 10% displayed increasing OC content with depth which may reflect recent ecosystem shifts, changing inputs, or subtle degradation (Fig. R1, below; not included in the manuscript).

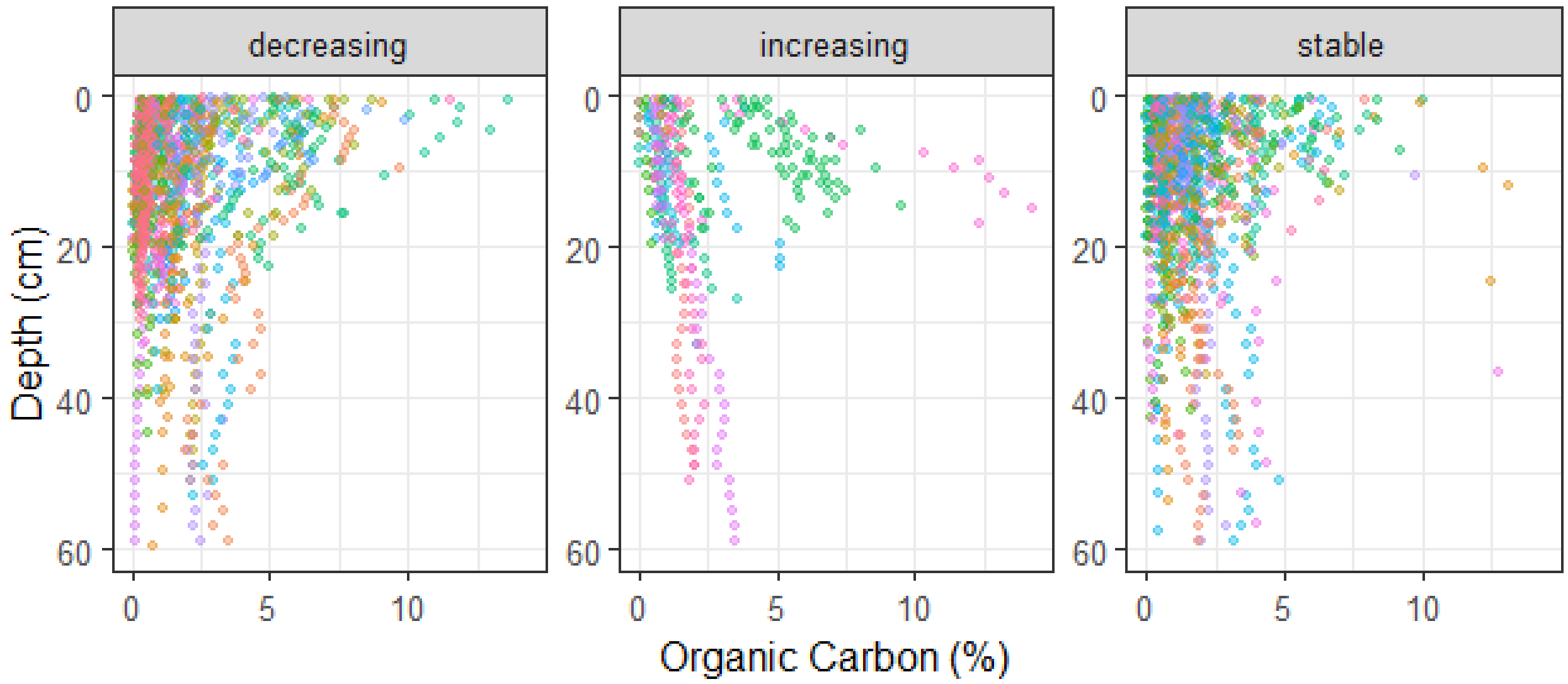

Figure R1. Organic carbon depth profiles in dated seagrass cores grouped by OC content trend with depth.

These empirical profile trends demonstrate that the vast majority of our compiled cores (~90%) display classic steady-state accumulation or natural diagenetic decay patterns rather than systematic post-disturbance OC loss or collapse. While a minority (10%) shows increasing trends of OC with depth that could hint at recent environmental change, our dataset as a whole reflects stable, long-term accumulation dynamics. Because the extent to which modern burial rates may be impacted by subtle, unmonitored ecological degradation remains unquantifiable across this global compilation, we refrain from extrapolating beyond these empirical profile trends. Additionally, humani impacts can increase or decrease OC burial and also the recalcitrance of OC, but future studies are required to estimate the global change impact on OC burial and to decipher the fate/permanence of seagrass OC under global change.

We have rearranged the methods section to clarify this aspect. Now we clarify upfront that during data compilation only natural sites were retained while those flagged as disturbed, degraded or restored in original publications were not included in the analysis.

L564-569: *"During data compilation and analysis, we excluded any sites identified as degraded, disturbed or restored in the primary literature as their OC burial rates might be affected by a shifting baseline or the time since planting, which varies between sites and does not align with the temporal resolution over which OC burial rates were assessed in the rest of the dataset's cores."*

Additionally, we have added a brief text in the Methods acknowledging that while our dataset targets non-degraded meadows, subtle long-term ecological shifts cannot be

completely ruled out through the global data compilation, though the vast majority of profile trajectories conform to steady-state accumulation models.

L675-681: *"Based on the downcore OC profile distributions, the vast majority of compiled cores (~90%) displayed classic steady-state accumulation or natural diagenetic decay rather than systematic seagrass disturbance. However, because establishing pre-industrial reference baselines requires longer term historical records, we did not analyze the potential for a shifting baseline in OC burial rates driven by increased human impact during the last century. Future studies are needed to quantify global change impacts on OC burial and decipher the fate and permanence of seagrass sediment carbon."*

3. The finding that adjacent bare sediments exhibit comparable OC burial rates and 13C offsets to vegetated sediments (Fig. 5) is ecologically fascinating. It highlights the "spillover" or "halo effect" of seagrass canopies. However, "unvegetated" is a relative term that requires tighter spatial definition. Please clarify the spatial scale of these adjacent bare cores (e.g., were they within the meadow patch matrix (gaps), 10 meters away, or 1 kilometer away?). From a hydrodynamic perspective, the seagrass canopy creates a 3D hydrodynamic shadow that extends beyond the meadow edge, facilitating fine particle deposition in nearby bare patches. Explicitly discussing this benthic-pelagic spatial coupling will strengthen the argument that seagrasses drive landscape-scale OC burial, even if the carbon ultimately settles outside the meadow footprint.

Thank you for this comment. Most of the bare sediment cores included in the synthesis come from studies that sampled both seagrass and unvegetated areas around the same site location. We calculated straight-line distances between the unvegetated cores and their nearest seagrass core at paired sites to better characterize these pairings. For the subset of studies where coordinates were available at the core level, distances ranged from 6 m to 3 km (median = 810 m).

We have clarified what unvegetated sediments represent in the introduction and in the discussion of the manuscript and additionally have mentioned the spillover effect in the Discussion section.

L124-127: *"The dataset comprised 332 sediment profiles from seagrass ecosystems across a latitudinal range of 64ºN to 35ºS (Fig. 1), including vegetated (89%) and unvegetated (11%) sediments. Unvegetated cores represented adjacent non-vegetated areas generally paired with nearby seagrass cores, separated by distances ranging from 6 m to 3 km".*

***L396-403**: "At the local scale where seagrass meadows and adjacent bare sediments were sampled within the same sites (separated by distances ranging from 6 m to 3 km), OC content and dry bulk density differed between habitats, whereas isotopic similarity to*

seagrass-derived organic matter, sediment accumulation and OC burial rates did not (Fig. 5c). Although limited in scope, these findings highlight the spillover effect of seagrass meadows suggesting that seagrasses may influence burial at the site level by contributing organic matter to nearby unvegetated sediments, as evidenced by their similar isotopic signatures of seagrass-derived OC[16] (Fig. 5c)."

4. From a geochronological standpoint, excluding or flagging cores with pervasive downcore mixing (Quality Flag 3, representing ~26% of cores) is completely justified to ensure accurate MAR calculations. However, from a benthic ecology perspective, this intense mixing (bioturbation by callianassid shrimp, polychaetes, etc.) is a fundamental driver of OC remineralization, as it introduces oxygen into deep sediments. The authors should briefly discuss the ecological implications of this exclusion. By mathematically sidestepping highly bioturbated sites, is there a risk of slightly overestimating or underestimating the global net preservation? Acknowledging bioturbation as a major biological filter for OC burial (not just a methodological hurdle) would integrate benthic ecology nicely with the geochemical narrative.

We would like to clarify an important distinction regarding our dataset handling: cores flagged for intense downcore mixing (Quality Flag 3) were not excluded from our primary analyses, provided that mass accumulation rates (MAR) or OC burial rates were reported in the original source publications (these represented ~26% of the total dataset). However, we conducted a sensitivity analysis comparing global OC burial rates calculated with and without these cores showing intense downcore mixing to assess if rates would be biased high. We observed no statistically significant difference between burial rate estimates calculated with and without these cores ($p > 0.05$). Consequently, all cores with reported rates were retained, yielding a global burial rate estimate of 25-33 g OC m^{-2} yr^{-1}. Mechanistically, while physical downcore mixing can inflate calculated MAR, sediment mixing may enhance oxygen exposure and accelerate OC remineralization. Across this global compilation, these opposing processes appear to counter-balance, resulting in similar geometric mean burial rates regardless of whether mixed profiles are included.

We have highlighted this in the Results:

L160-163: *Considering all depositional seagrass sediments, the global geometric mean OC burial rate was 25 ± 3 g OC m-2 yr-1 (95% CI: 22–29; n = 207), a value that remained similar even when excluding records affected by intense sediment mixing (27 ± 2 g OC m-2 yr-1; 95% CI: 23–31; n = 127).*

And Methods:

L601-605: *"Cores exhibiting intense downcore mixing (^{210}Pb profile quality flag 3) for which OC burial rates were reported (26% of cores) were included in the main analyses. However, global burial rates were computed with and without these values to assess the potential influence of ^{210}Pb profile quality on OC burial estimates, particularly the extent to which downcore mixing may bias rates high".*

5. In the discussion (Lines 376-401), the authors attribute high OC stocks to persistent species (Posidonia, Thalassia), which is accurate. However, the explanation relies heavily on their sedimentary environment (MAR). I suggest adding a brief mechanistic explanation based on plant functional traits. Persistent species not only occupy specific geomorphic settings but also invest heavily in belowground biomass (extensive root/rhizome networks) and possess highly recalcitrant structural tissues (high lignin/cellulose, high C:N/C:P ratios) compared to colonizing species like Halophila. Emphasizing that the intrinsic chemical recalcitrance of the plant tissue actively resists microbial degradation will strengthen the biological rationale behind the observed taxonomic patterns.

We agree with the reviewer and have added text to the manuscript addressing this suggestion

L330-336: *"Sediments colonized by persistent seagrass species of the genera Posidonia and Thalassia, or by mixed meadows of persistent and opportunistic taxa (e.g., Posidonia with Amphibolis, or Thalassia with Syringodium), exhibited the highest sediment OC content (medians between 2.2%, 1.4% and 1.9%, respectively; $p < 0.05$; Fig. S4a), consistent with previous findings that persistent species support the largest sediment OC stocks globally[17]. Mechanistically, persistent genera feature large belowground biomass investments and highly recalcitrant structural tissues that resist microbial mineralization[35,39]."*

L350-353: *"Sediments colonized by Halophila spp. in China[20], Australia[43], and the eastern Medtirerranean[44], showed some of the highest MAR, yet yielded comparatively low OC burial rates due to low sediment OC content. This might be related with the low belowground biomass and rapid tissue turnover characteristic of colonizing taxa[35]."*

minor comments

1. In the Abstract, simply reporting the global OC burial rate of seagrass meadows is not sufficient and lacks novelty. The manuscript also discusses the relationship between sediment OC content and OC burial rates, and this should be highlighted in the Abstract.

Done as suggested

2. In Figure 2, adding the percentage values representing the proportional seagrass area of each bioregion to the pie chart.

Done as suggested

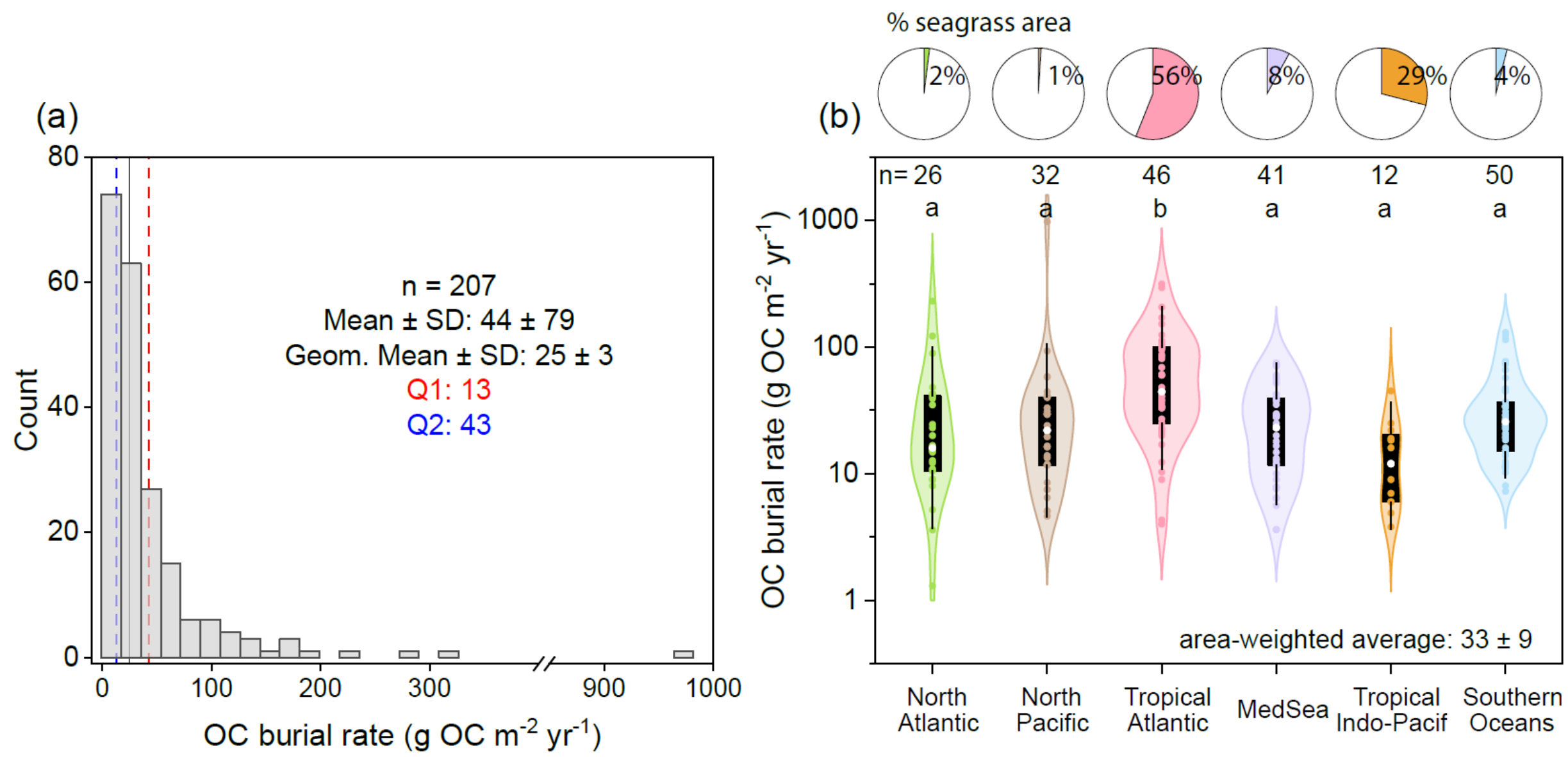

3. Please provide the sample sizes for the boxplots in Figures 4 and 5.

Done as suggested

4. According to the manuscript, global seagrass extent is reported as a range (247,800–366,400 km^2). It is therefore unclear why the relative area contribution of each seagrass bioregion was treated as a fixed value. Please provide a clearer explanation in the Methods section regarding how the bioregional area proportions were derived.

The relative area contributions of each bioregion are reported as fixed percentages because the underlying bioregional extent estimates scale proportionally between the lower and upper bounds of the global range. Specifically, because the lower boundary (247,800 km^2) and upper boundary (366,400 km^2) are derived by summing the corresponding lower and upper extent estimates across all individual bioregions, the relative proportion contributed by any given bioregion remains similar.

We have updated the Methods section to explain how bioregional area proportions were calculated.

L698-703: "*The relative area contribution for each bioregion was calculated by dividing the reported spatial extent of that bioregion by the total global seagrass extent (Table S5).*

Because global lower and upper seagrass area bounds were calculated by summing the respective lower and upper area estimates across all bioregions, these regional extent proportions remain similar and were applied as constant percentage weights in the global area-weighted average".

5. For Figure 4, although the authors describe the criteria used to group OC burial rates in the Statistical analysis section, it is suggested that the actual numerical range corresponding to each group be provided, which would make the figure more intuitive and easier to understand. In addition, MAR also appears to be grouped in this figure, and the criteria for grouping MAR should also be clearly described in the Statistical analysis section.

The range corresponding to each OC burial group has been added to panel a) and it is also provided in Table S3.

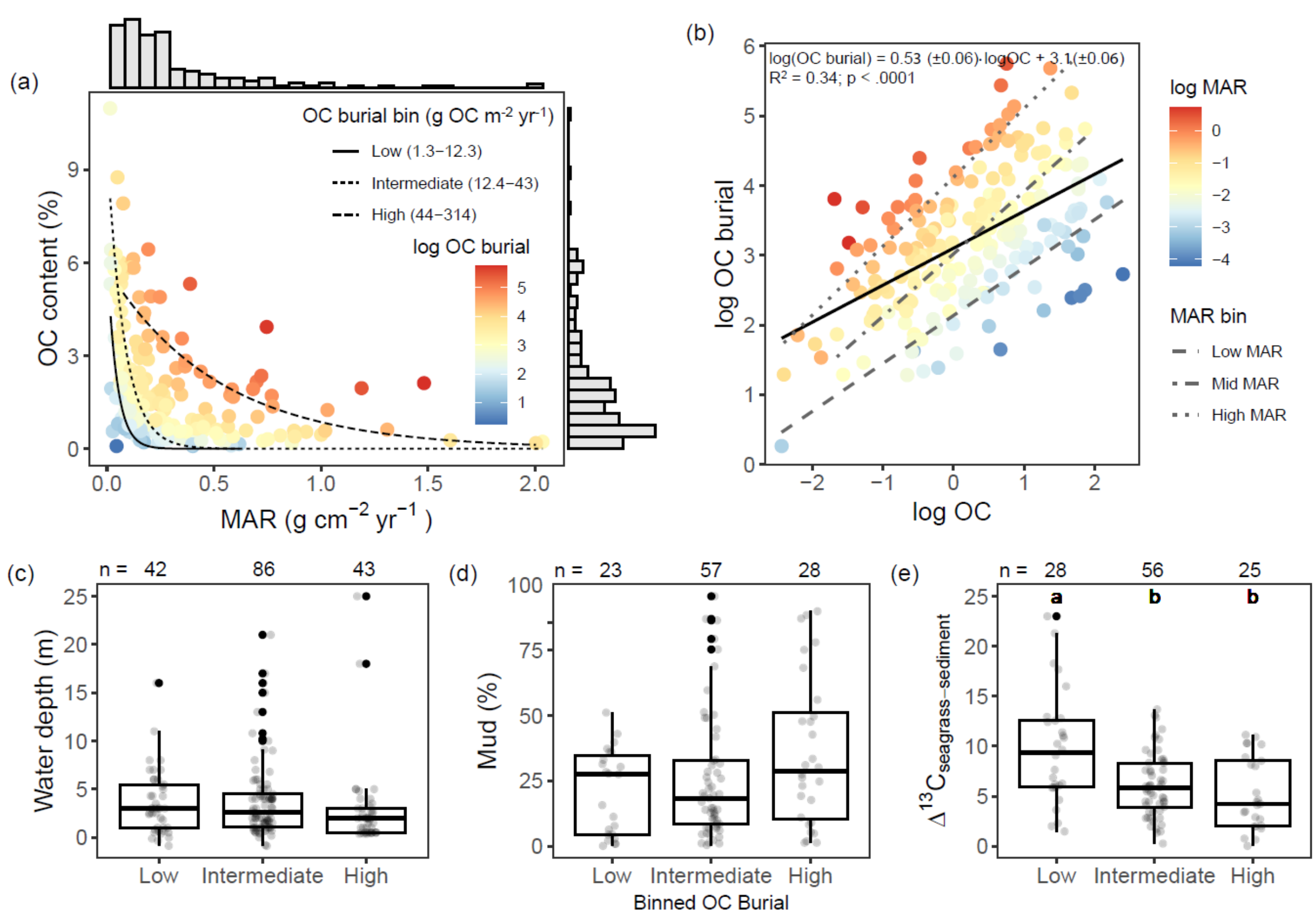

We clarified in the Methods how MAR rates were grouped. Revised text now reads **L747-752**: *"Burial rates were grouped into quantile intervals (low: 0–25%, intermediate: 25–75%, high: 75–100%) to identify thresholds associated with contrasting depositional settings. Similarly, MAR were grouped into the same quantile intervals. Within each interval log–log regressions were used to assess how OC burial scales with OC content, and the resulting slopes were compared to interpret sediment OC preservation under varying sedimentation rates.*

6. The sediment core dataset is stated to be available through the Smithsonian's Figshare data repository: https://doi.org/10.25573/serc.32592150. However, this database link cannot be accessed.

The DOI link was reserved prior to publication and will become active once the manuscript is published. In the meantime, the dataset is accessible for review via the following private view/download link: https://figshare.com/s/bda2144e873b0bf9983c. We apologize for not including this private link in our original response to reviewer 1 comments.
